# Therapeutic potential of procathepsin L-inhibiting and progesterone-entrapping dimethyl-β-cyclodextrin nanoparticles in treating experimental sepsis

**DOI:** 10.3389/fimmu.2024.1368448

**Published:** 2024-03-14

**Authors:** Xiaoling Qiang, Weiqiang Chen, Cassie Shu Zhu, Jianhua Li, Timothy Qi, Li Lou, Ping Wang, Kevin J. Tracey, Haichao Wang

**Affiliations:** ^1^ The Feinstein Institutes for Medical Research, Northwell Health, Manhasset, NY, United States; ^2^ Donald and Barbara Zucker School of Medicine at Hofstra/Northwell, Hempstead, NY, United States

**Keywords:** innate immune cells, procathepsin-L, progesterone, 2,6-dimethyl-β-cyclodextrin, sepsis

## Abstract

The pathogenic mechanisms of bacterial infections and resultant sepsis are partly attributed to dysregulated inflammatory responses sustained by some late-acting mediators including the procathepsin-L (pCTS-L). It was entirely unknown whether any compounds of the U.S. Drug Collection could suppress pCTS-L-induced inflammation, and pharmacologically be exploited into possible therapies. Here, we demonstrated that a macrophage cell-based screening of a U.S. Drug Collection of 1360 compounds resulted in the identification of progesterone (PRO) as an inhibitor of pCTS-L-mediated production of several chemokines [e.g., Epithelial Neutrophil-Activating Peptide (ENA-78), Monocyte Chemoattractant Protein-1 (MCP-1) or MCP-3] and cytokines [e.g., Interleukin-10 (IL-10) or Tumor Necrosis Factor (TNF)] in primary human peripheral blood mononuclear cells (PBMCs). *In vivo*, these PRO-entrapping 2,6-dimethal-β-cyclodextrin (DM-β-CD) nanoparticles (containing 1.35 mg/kg PRO and 14.65 mg/kg DM-β-CD) significantly increased animal survival in both male (from 30% to 70%, n = 20, *P* = 0.041) and female (from 50% to 80%, n = 30, *P* = 0.026) mice even when they were initially administered at 24 h post the onset of sepsis. This protective effect was associated with a reduction of sepsis-triggered accumulation of three surrogate biomarkers [e.g., Granulocyte Colony Stimulating Factor (G-CSF) by 40%; Macrophage Inflammatory Protein-2 (MIP-2) by 45%; and Soluble Tumor Necrosis Factor Receptor I (sTNFRI) by 80%]. Surface Plasmon Resonance (SPR) analysis revealed a strong interaction between PRO and pCTS-L (K_D_ = 78.2 ± 33.7 nM), which was paralleled with a positive correlation between serum PRO concentration and serum pCTS-L level (ρ = 0.56, *P* = 0.0009) or disease severity (Sequential Organ Failure Assessment, SOFA; ρ = 0.64, *P* = 0.0001) score in septic patients. Our observations support a promising opportunity to explore DM-β-CD nanoparticles entrapping lipophilic drugs as possible therapies for clinical sepsis.

## Introduction

Bacterial infections and associated sepsis are probably the most prominent causes of death in hospitals, accounting for almost 20% of total deaths globally (1). Its pathogenic mechanisms partially attribute to dysregulated inflammatory responses to microbial infections that are initiated by early cytokines [e.g., interleukin-1 (IL-1) and tumor necrosis factor (TNF)] but sustained by late-acting mediators including high mobility group box 1 (HMGB1) (2, 3) and procathepsin-L (pCTS-L) (4, 5). For example, upon initial innate recognition of many “pathogen-associated molecular patterns molecules” (PAMPs, e.g., bacterial lipopolysaccharide, LPS) by corresponding pattern recognition receptors (PRRs) such as the toll-like receptor 4 (TLR4) (6), monocytes and macrophages sequentially produce “early” cytokines [e.g., TNF and interleukin-1β (IL-1β)] (7–9), toxic chemicals (e.g., lactate) (10–12), and late-acting mediators such as HMGB1 (2) and pCTS-L (4). In comparison with early cytokines, these late-acting mediators can be therapeutically targeted in delayed regimens (4, 13), thereby offering relatively wider therapeutic windows (5). It is therefore necessary to find small molecule drugs capable of suppressing pCTS-L-mediated dysregulated inflammation to develop potential therapeutic strategies for inflammatory diseases.

The US Drug Collection contains 1360 FDA-approved small molecule drugs that have reached clinical trials as evidenced by their assignment of the United States Adopted Names (USAN) and inclusion in the United States Pharmacopeia (USP) Dictionary, the authorized list of established names for drugs in the United States. These small molecule drugs can interact with specific protein targets in the body to confer a therapeutic effect, but possess distinct advantages such as oral administration convenience, target specificity, cell penetration, and cost-effectiveness. They serve as a basis for high throughput screening of established drugs for potential new activities (14). In this study, we developed a 96-well-based assay to screen for small molecule drugs that could suppress the pCTS-L-mediated dysregulated inflammation and confer protection against experimental sepsis. Here, we presented substantial evidence to suggest: i) a hormone, progesterone (PRO), as an inhibitor of pCTS-L-mediated dysregulated inflammation; and ii) a PRO-entrapping 2,6-dimethyl-β-cyclodextrin (DM-β-CD) nanoparticles as a potential therapy in a preclinical setting.

## Materials and methods

### Materials

Murine macrophage RAW 264.7 cell line was purchased from the American Type Culture Collection (ATCC). Human blood samples were obtained from the New York Blood Center (Long Island City, NY, USA) to harvest primary human peripheral blood mononuclear cells (PBMCs) by density gradient centrifugation as previously described (4, 15, 16). Both macrophage cultures and PBMCs were routinely incubated in DMEM media containing 1% streptomycin/penicillin and 10% fecal bovine serum or 10% human serum. When cell densities reached 80-90% confluence, adherent macrophages or PBMCs were stimulated with bacterial endotoxins (lipopolysaccharides, LPS, *E. coli* 0111:B4, #L4130, Sigma-Aldrich) or recombinant human or murine pCTS-L protein in the absence or in the presence of each of the 1360 compounds in the U.S. Collection of Drug (10 mM in DMSO), as well as progesterone (Cat. # P0130, Sigma-Aldrich) solubilized in ethanol (5 mg/ml) or entrapped into 2,6-dimethyl-β-cyclodextrin (DM-β-CD, Cat. #H0513, Sigma-Aldrich) nanoparticles. The progesterone-carrying DM-β-CD nanoparticles (containing 84.4 mg progesterone per 1000 mg of the PRO/DM-β-CD complex) were also purchased from Sigma-Aldrich (Cat. # P7556). The extracellular levels of various cytokines and chemokines were respectively measured by using ELISA kits or Cytokine Antibody Arrays as previously described (4, 15, 16).

### Generation and purification of recombinant murine and human pCTS-L proteins

Recombinant murine and human pCTS-L proteins containing an N-terminal 6×Histidine tag were respectively expressed in *E. coli* BL21 (DE3) pLysS cells and purified to homogeneity as previously described (4, 16). Briefly, upon sonication to disrupt the bacterial cell wall, the inclusion bodies containing pCTS-L proteins were harvested via differential centrifugation technique following sequential washings in 1% Triton X-100 dissolved in 1 × PBS buffer The purified inclusion bodies were subsequently dissolved in high concentration of urea solution (8.0 M), and then refolded by dialysis in Tris buffer (10 mM, pH 8.0). Afterward, recombinant pCTS-L proteins were purified by histidine-affinity chromatography technique and Triton X-114 extractions. The endotoxin content of recombinant pCTS-L proteins was estimated to be < 0.01 U per µg of pCTS-L protein.

### High-throughput screening of U.S. collection of drugs for pCTS-L inhibitors

We obtained the U.S. Collection of 1360 drugs (**Supplementary Table 1**, 10 mM in DMSO) from the MicroSource Discovery System Inc., and used this chemical library to search for potential pCTS-L inhibitors as previously described (16). Briefly, murine macrophage-like RAW 264.7 cells were challenged with murine pCTS-L protein in the absence or in the presence of each drug at several concentrations for 16 h, and levels of TNF in macrophage-conditioned medium was measured using specific TNF DuoSet ELISA kit (Cat# DY410, R&D Systems).

### Murine or Human Cytokine Antibody Arrays

Murine Cytokine Antibody Array Kits (Cat.# AAM-CYT-3-8, RayBiotech Inc., Norcross, GA, USA) were employed to measure the relative levels of 62 cytokines/chemokines in macrophage cell culture-conditioned medium or murine serum as previously described (4, 15, 16). Similarly, human Cytokine Antibody C3 Array Kits (Cat.# AAH-CYT-3-8) were employed to measure the relative levels of 42 cytokines/chemokines in human PBMC-conditioned culture medium as previously described (4, 15, 16).

### Animal model of experimental sepsis

Adult male and female Balb/C mice (7-8 weeks old, 20-25 g body weight) were purchased from Charles River Laboratories (Wilmington, MA), housed in a temperature-controlled room on a 12-h light-dark cycle, and acclimated for at least 5-7 days before usage. Every attempt was made to limit the number of animals used in the present study as per the ARRIVE guidelines for reducing the number of animals in scientific research developed by the British National Centre for the Replacement, Refinement and Reduction of Animals in Research (NC3Rs). Additionally, all experiments were performed in accordance with the International Expert Consensus Initiative for Improvement of Animal Modeling in Sepsis - Minimum Quality Threshold in Pre-Clinical Sepsis Studies (MQTiPSS) (17), which includes practices such as randomization of animals in each experimental group, delayed therapeutic interventions with therapeutic agents (e.g., PRO-entrapping DM-β-CD nanoparticles) (18), establishment of specific criteria for euthanasia of moribund septic animals (e.g., labored breathing, minimized response to human touch, and immobility), as well as the administration of fluid resuscitation and antibiotics (19). This study was administratively approved by the IACUC of the Feinstein Institutes for Medical Research (FIMR, Protocol # 2017-003 Term II; Date of Approval, April 28th, 2020).

Adult male or female Balb/C mice aged 7-8 weeks and weighing 20-25 g underwent a surgical procedure referred to as “cecal ligation and puncture” (CLP) to induce experimental sepsis as previously outlined (4, 15, 16). Briefly, the cecum of Balb/C mice was surgically exposed, ligated approximately 5.0 mm from the cecal tip, and punctured once with a 22-gauge syringe needle. Prior to CLP surgery, all experimental animals received a buprenorphine injection (0.05 mg/kg, s.c.) to alleviate immediate surgical pain, because repetitive use of buprenorphine in the CLP model could paradoxically elevate sepsis surrogate markers and animal lethality (20, 21), leading to unnecessary use of more animals per experimental group. Additionally, a small dose of bupivacaine and lidocaine was locally injected around the incision site immediately after CLP surgery. Approximately 30 min post CLP surgery, all experimental animals were subcutaneously injected with imipenem/cilastatin (0.5 mg/mouse) (Primaxin, Merck & Co., Inc.), followed by resuscitation with sterile saline solution (20 ml/kg). Septic animals were only given a single dose of antibiotics before pharmacological administration of PRO-entrapping DM-β-CD nanoparticles at 24 h post CLP, worrying that subsequent antibiotics treatment may adversely affect the therapeutic efficacy of PRO-entrapping DM-β-CD nanoparticles. Before treatment, animals were randomly assigned to control vehicle and experimental groups, and PRO dissolved in sesame oil (Cat. #S3547, Sigma-Aldrich; 2.0 mg/ml) or water after complexation with DM-β-CD to form nanoparticles (containing 84.4 mg PRO per 1000 mg of PRO/DM-β-CD complex) was intraperitoneally injected to septic mice at various time points post CLP surgery. Animal survival was observed for two weeks to ensure no late death occurred. To elucidate the potential protective mechanisms of PRO-entrapping DM-β-CD nanoparticles, a separate group of Balb/C mice were subjected to CLP, and DM-β-CD vehicle (14.65 mg/kg) or PRO-entrapping DM-β-CD nanoparticles (containing 1.35 mg/kg PRO and 14.65 mg/kg DM-β-CD) were administered at 2 h and 20 h post CLP. At 24 h post CLP, animals were euthanized to collect blood and measure serum levels of various cytokines and chemokines using murine Cytokine Antibody Arrays or markers of tissue injury using specific colorimetric enzymatic assays.

### Measurement of tissue injury markers

Blood samples were harvested at 24 h post CLP following intraperitoneal administrations of DM-β-CD vehicle (14.65 mg/kg) or PRO-entrapping DM-β-CD nanoparticles (containing 1.35 mg/kg PRO and 14.65 mg/kg DM-β-CD) at 2 h and 20 h post CLP, and centrifuged at 3000 x g for 10 min to collect serum. Serum levels of liver injury markers such as aspartate aminotransferase (AST, Cat. No. 7561), and lactate dehydrogenase (LDH, Cat. No. 7572) were determined using specific colorimetric enzymatic assays (Pointe Scientific, Canton, MI) according to manufacturer’s instructions as previously described (15).

### Open Surface Plasmon Resonance (SPR)

We employed the Nicoya Lifesciences’ gold-nanoparticle-based Open Surface Plasmon Resonance (OpenSPR) technology (Kitchener, ON, Canada) to characterize protein-drug interaction following the manufacturer’s instructions. Briefly, recombinant pCTS-L was immobilized on NTA sensor chip (Cat. # SEN-Au-100-10-NTA) as previously described (4, 22), and DMSO solution of PRO was applied as an analyte at different concentrations. The sensorgrams of the dynamic ligand-analyte interaction were recorded over time to estimate the equilibrium dissociation constant (K_D_) (Nicoya Lifesciences).

### Systemic accumulation of PRO and pCTS-L in septic patients

This study was administratively approved by the institutional review board (IRB) of the FIMR (IRB protocol #18-0184) and consented by all patients participants who were diagnosed with sepsis or septic shock based on the Sepsis-3 criteria (23). Small volume of blood samples (5.0 ml) was obtained from eleven septic patients recruited to the Long Island Jewish Medical Center or North Shore University Hospital between 2018-2019 at three time points: Time 0 (within 24 h of the initial diagnosis); Time 24 h (24 h post the initial diagnosis); and Time 72 h (72 h post the initial diagnosis). The demographics of these eleven septic patients have been reported previously (4), but briefly described in the **Supplementary Table 2**. These clinical samples were assayed for pCTS-L levels using human pCTS-L ELISA kit (Cat.# MBS7254442, MyBioSource.com) with reference to standard cure generated from using recombinant human pCTS-L. In parallel, the serum concentrations of progesterone were measured by using highly sensitive ELISA kit (Cat. # ADI-901-011, ENZO Life Sciences, Inc., Farmingdale, NY, USA).

### Statistical analysis

All data were initially evaluated for normality by the Shapiro-Wilk test before conducting appropriate statistical tests. The Student’s t test was used to compare two independent experimental groups. For comparison among multiple groups with non-normal (skewed) distribution (as assessed by the Shapiro-Wilk test), the statistical difference was evaluated with the non-parametric Kruskal-Wallis ANOVA test followed by the Dunn’s test. The Kaplan-Meier method was employed to compare the differences in mortality rates between two different groups along with the nonparametric log-rank *post hoc* test. Finally, the Spearman rank correlation coefficient test was used to evaluate associations between two quantitative variables that exhibited non-normal distribution. Statistical significance was defined as a *P* value less than 0.05.

## Results

### Identification of PRO as an inhibitor of pCTS-L-mediated TNF secretion

**Figure 1:**
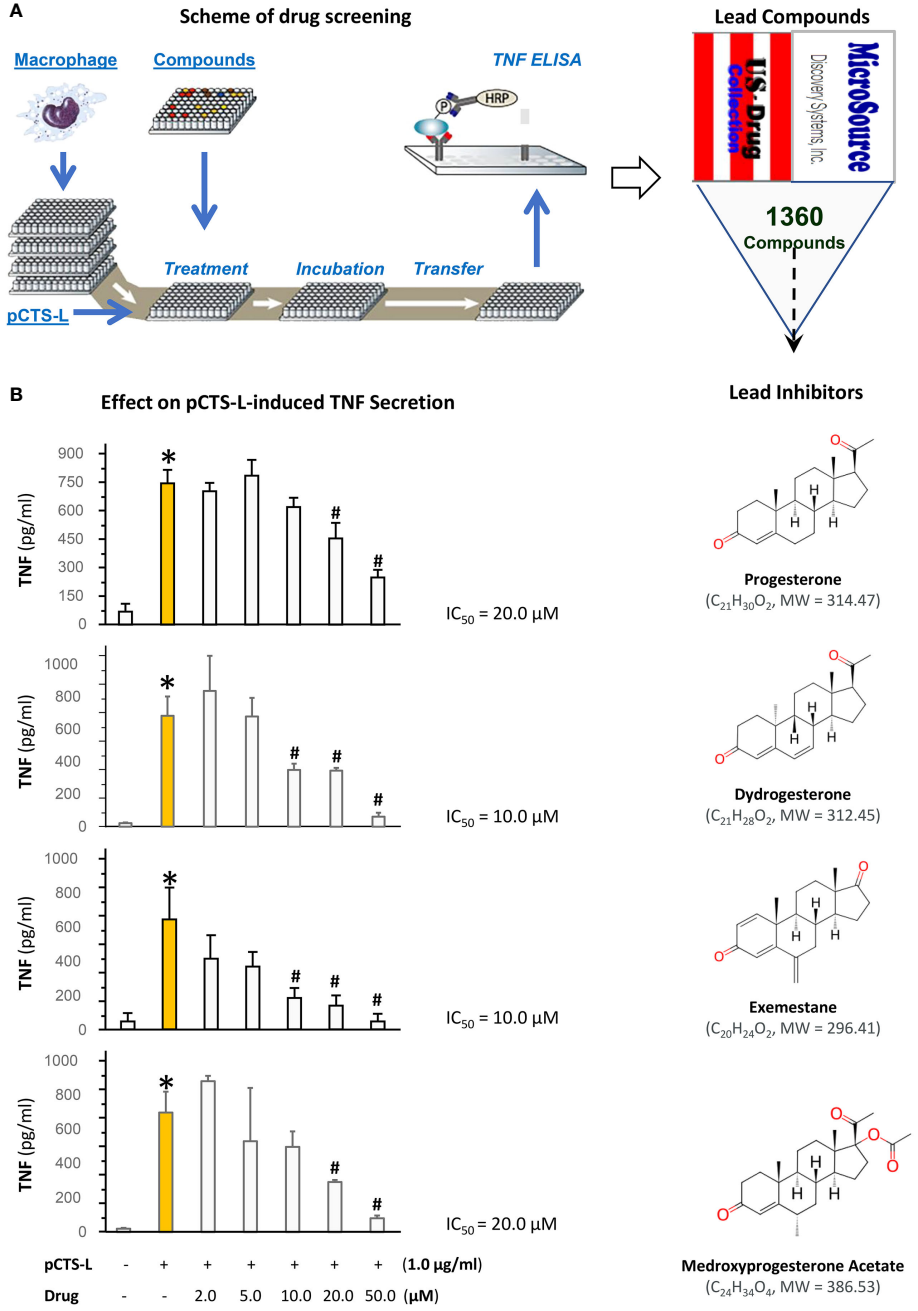
High-throughput screening of a U.S. Collection of 1360 Drugs for potential inhibitors of pCTS-L-mediated TNF production. **(A)** The scheme for the 96-well plate-based high-throughput screening of potential pCTS-L inhibitors was adapted from our recent publication (16). Murine macrophage-like RAW 264.7 cells were cultured until 80-90% confluence and challenged with recombinant murine pCTS-L in the absence or in the presence of each of the 1360 compounds at different concentrations for 16 h. The levels of TNF in the macrophage-conditioned medium were measured by using ELISA. **(B)** Effects of progesterone and three analogs on pCTS-L-induced TNF secretion by murine macrophage cultures. Note that all four lead compounds dose-dependently inhibited pCTS-L-induced TNF secretion by murine macrophage cultures. *, *P* < 0.05 versus “- pCTS-L”; #, *P* < 0.05 versus “+ pCTS-L”, non-parametric Kruskal-Wallis ANOVA test.

To explore novel pCTS-L inhibitors, we adapted a macrophage cell-based bioassay that we recently developed (16) to screen a U.S. Collection of 1360 drugs supplied as 10.0 mM Dimethyl Sulfoxide (DMSO) solutions in seventeen 96-well microplates for possible activities to inhibit pCTS-L-stimulated TNF secretion (**Figure 1A**). We optimized the experimental conditions by respectively titrating the concentration of pCTS-L protein (to 1.0 μg/ml) and the confluence of macrophage cultures (to 80-90%). A complete screening of 1360 drugs of the U.S. Collection resulted in the identification of progesterone (PRO) and three analogs (i.e., dydrogesterone, exemestane, and medroxyprogesterone acetate; **Figure 1B**) as inhibitors of pCTS-L-stimulated TNF production. When dissolved in DMSO, PRO dose-dependently attenuated pCTS-L-mediated TNF secretion with an estimated IC around 20.0 µM (**Figure 1B**) without affecting mitochondrial metabolic activity (MTT assay) or cell viability (Trypan blue uptake**, Supplementary Figure 1**).

### PRO inhibited pCTS-L-mediated secretion of TNF and several chemokines in primary human peripheral blood mononuclear cells (PBMCs)

**Figure 2:**
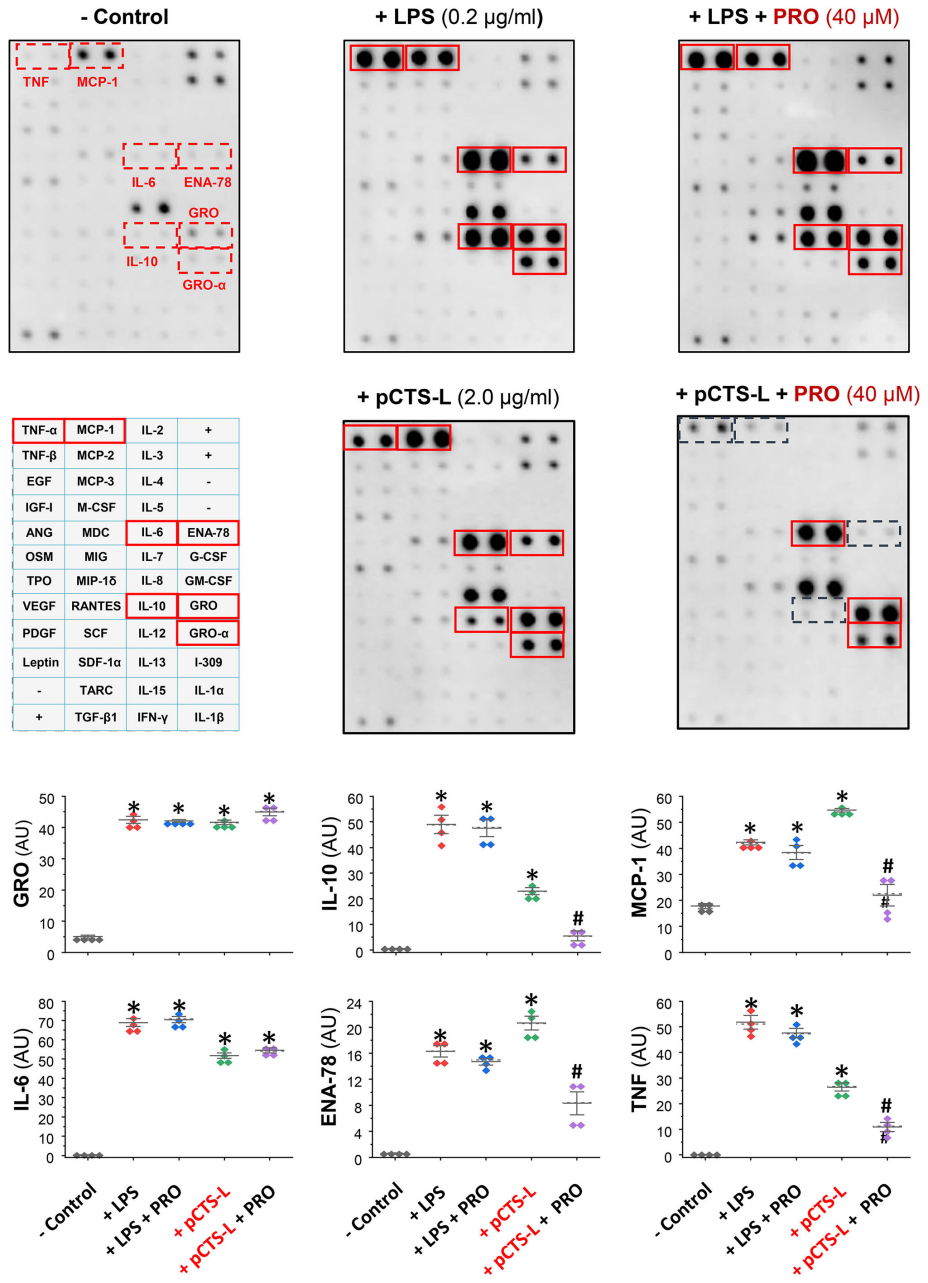
Progesterone selectively suppressed the pCTS-L-mediated productions of several cytokines/chemokines in primary human peripheral blood mononuclear cells (PBMCs). Human PBMCs were challenged with human pCTS-L (2.0 μg/ml) or LPS (0.2 μg/ml) in the absence or in the presence of PRO (40 μM in ethanol) for 16 h. The levels of various cytokines and chemokines in human PBMC-conditioned medium were measured by using Cytokine Antibody Array kits (in arbitrary units, AU). *, P < 0.05 versus “- pCTS-L”; #, P < 0.05 versus “+ pCTS-L”, non-parametric Kruskal-Wallis ANOVA test.

To confirm PRO’s pCTS-L-inhibitory activities, we first dissolved it in ethanol before testing its effects on pCTS-L-stimulated production of 42 cytokines and chemokines in primary human PBMCs. In agreement with our earlier report (4), pCTS-L markedly stimulated the secretion of a few chemokines (e.g., ENA-78, GRO, and MCP-1) and cytokines (e.g., TNF, IL-6 and IL-10) (**Figure 2**). However, the pCTS-L-mediated TNF production was markedly inhibited by the co-administration of PRO at a relatively high dose (40 μM, **Figure 2**), an almost most effective dose that suppressed pCTS-L-induced TNF secretion in dose-response studies using murine macrophages (**Figure 1B**) as well as primary PBMCs from several different donors. Likewise, the pCTS-L-triggered secretion of several other cytokines (e.g., IL-10) and chemokines (e.g., ENA-78 and MCP-1) was similarly suppressed by PRO (**Figure 2**). Consistent with our earlier reports (16, 24), endotoxins effectively stimulated the secretion of several chemokines (MCP-1, ENA-78 and GRO) and cytokines (e.g., TNF, IL-6 and IL-10) in primary human PBMCs (**Figure 2**). However, PRO was unable to affect LPS-induced production of any cytokines/chemokines (**Figure 2**) even at the concentration that markedly inhibited pCTS-L-mediated cytokine/chemokine production, suggesting that PRO selectively inhibited pCTS-L-mediated inflammation.

### PRO-carrying 2,6-dimethyl-β-cyclodextrin (DM-β-CD) nanoparticles similarly inhibited pCTS-L-induced inflammation

**Figure 3:**
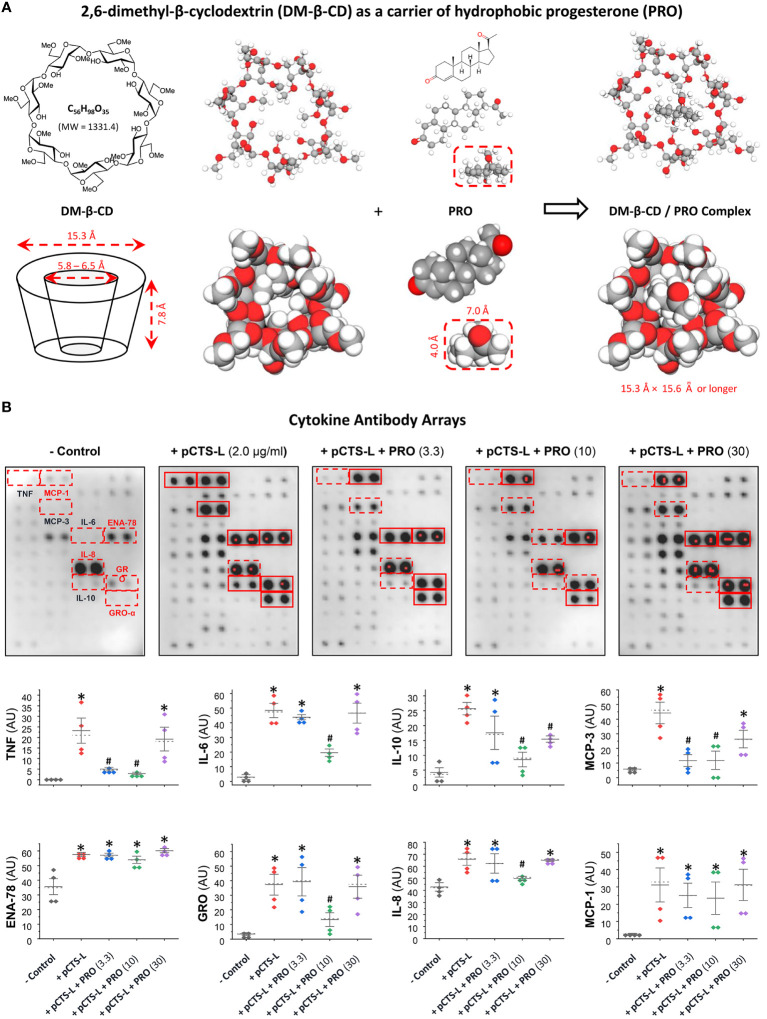
PRO-carrying DM-β-CD nanoparticles significantly inhibited pCTS-L-stimulated production of several cytokines and chemokines in human PBMCs. **(A)** Structures of DM-β-CD and PRO as well as the inclusion complex of these two molecules. Given its lipophilic nature, PRO can easily be intercalated and embedded into the hydrophobic inner cavity of the truncated cone structure of two DM-β-CD molecules. **(B)** PRO significantly inhibited pCTS-L-stimulated production of several cytokines and chemokines. Human PBMCs were challenged with recombinant human pCTS-L in the absence or in the presence of DM-β-CD/PRO complexes at different concentrations (µM) for 16 h, and extracellular concentrations of cytokines and chemokines were measured by using Cytokine Antibody Arrays. *, P < 0.05 versus “- pCTS-L”; #, P < 0.05 versus “+ pCTS-L”, non-parametric Kruskal-Wallis ANOVA test.

The β-cyclodextrin (β-CD) is defined as cyclic oligosaccharides of seven glucopyranoses in its β-chair conformation (**Figure 3A**), thereby displaying the shape of truncated cone with an outer hydrophilic surface that render it water-soluble and an inner hydrophobic core (25) that can entrap hydrophobic molecules (e.g., PRO). To increase the water-solubility of β-CD, the hydroxyl groups of the inner hydrophobic cavity (position 2) and the outer hydrophilic surface (position 6) of β-CD can be substituted with hydrophobic methyl moieties to produce the 2,6-dimethyl-β-cyclodextrin (DM-β-CD, **Figure 3A**), which still displayed the shape of cone with slightly less symmetry and smaller inner cavity (**Figure 3A**). As a lipophilic sterol, PRO could be inserted into the hydrophobic inner cavity of two DM-β-CD molecules at 1:2 molar ratio (**Figure 3A**). These PRO-carrying DM-β-CD nanoparticles dose-dependently suppressed pCTS-L-mediated production of a few cytokines (e.g., TNF, IL-6 and IL-10) and chemokines (GRO, IL-8 and MCP-3) in human PBMCs (**Figure 3B**), confirming that PRO-carrying DM-β-CD nanoparticles maintained pCTS-L-inhibiting properties of PRO under pharmacological conditions.

### PRO-carrying DM-β-CD nanoparticles protected both male and female mice from microbial infections

**Figure 4:**
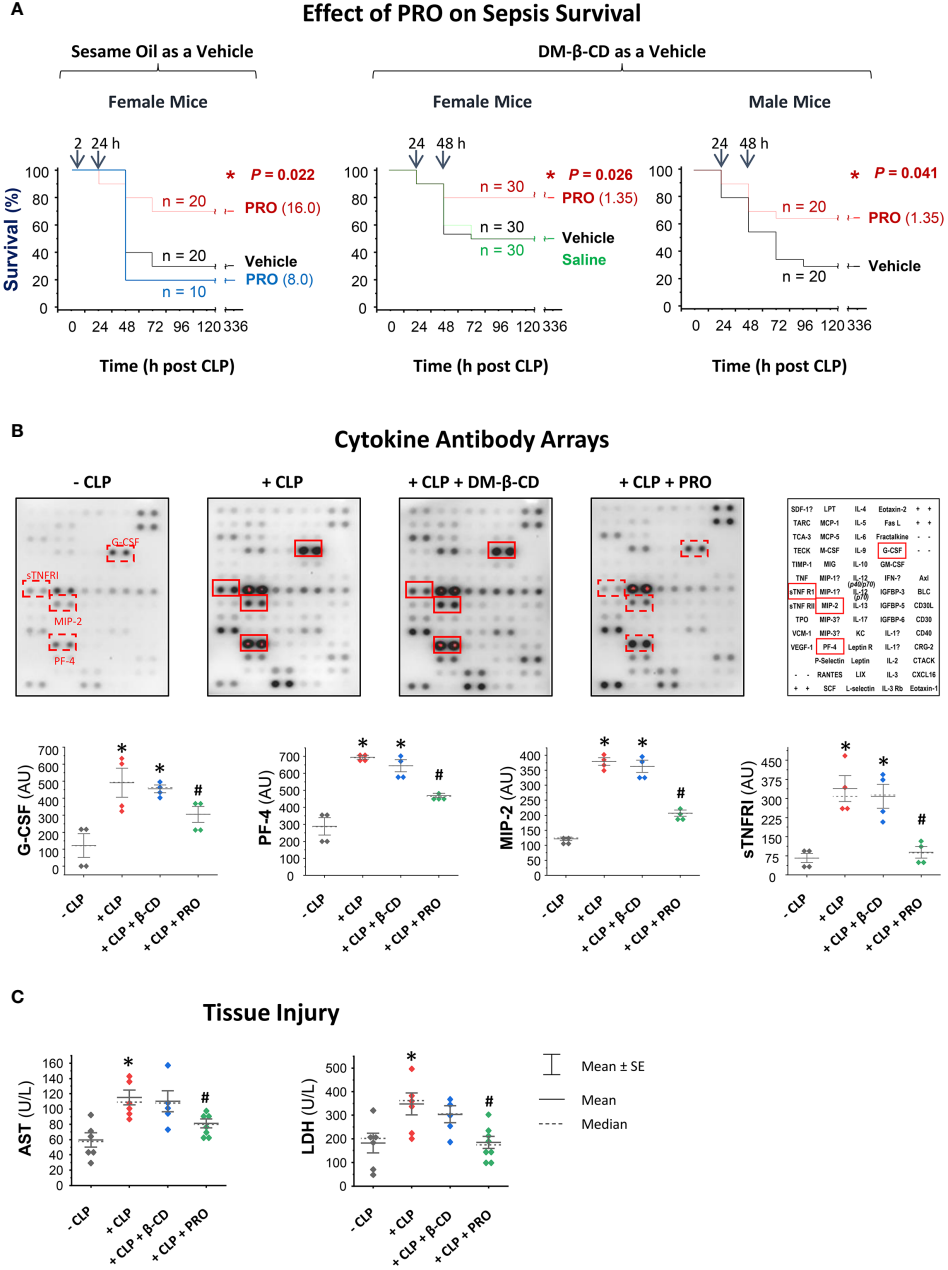
PRO-carrying DM-β-CD nanoparticles rescued mice from sepsis partially by attenuating sepsis-triggered inflammation. **(A)** PRO-containing sesame oil or PRO-entrapping DM-β-CD nanoparticles protected or rescued mice from experimental sepsis. Male (M) and/or female (F) Balb/C mice were subjected to CLP, and PRO-containing sesame oil or PRO/DM-β-CD nanoparticles were intraperitoneally administered at indicated doses and time points. *, P < 0.05 versus saline or vehicle (sesame oil or DM-β-CD) controls. **(B, C)** PRO-carrying DM-β-CD nanoparticles attenuated CLP-triggered systemic inflammation and tissue injury. Balb/C mice were subjected to CLP, and PRO/DM-β-CD nanoparticles (containing 1.35 mg/kg PRO and 14.65 mg/kg DM-β-CD) or DM-β-CD vehicle (14.65 mg/kg) were administered at 2 h and 20 h post CLP, and then euthanized at 24 h post CLP to collect blood and measure serum levels of various cytokines and chemokines (in arbitrary units, AU) as well as markers of tissue injury (AST and LDH) using specific colorimetric enzymatic assays. *, P < 0.05 versus “ - CLP”; #, P < 0.05 versus “+ CLP”, non-parametric Kruskal-Wallis ANOVA test.

To assess the PRO’s therapeutic efficacy, we first dissolved PRO in Sesame oil containing various unsaturated fatty acids that could emulsify and dissolve lipophilic PRO in the form of micelles (26–30). When dissolved in sesame oil, PRO did not show any protection against sepsis within a dose range of 1.0 mg/kg to 8.0 mg/kg (**Figure 4A**, Left Panel). When given intraperitoneally at a relatively high dose (16 mg/kg) at 2 h and 24 h post CLP, PRO promoted a marked protection against sepsis, significantly increasing animal survival rates from 30% to 70% (**Figure 4A**). In an effort to enhance its pharmacological efficacy, PRO was entrapped into DM-β-CD nanoparticles to enhance its water-solubility and bioavailability, and further tested in an animal model of experimental sepsis induced by cecal ligation and puncture (CLP). The PRO-carrying DM-β-CD nanoparticles was given at 24 h after CLP, which was a time point when circulating pCTS-L plateaued and some animals started to succumb to death (4). As a vehicle control, DM-β-CD (14.65 mg/kg) itself did not affect animal survival rate when it was given at 24 h and 48 h post CLP surgery (**Figure 4A**). In contrast, the PRO-carrying DM-β-CD nanoparticles significantly rescued both male and female mice from infections even when the 1st dose was intraperitoneally administered at 24 h post CLP (**Figure 4A**). This effective protective dose of PRO entrapped in the DM-β-CD nanoparticles (even when initially given at 24 h post CLP) was almost 10-fold lower than the dosage of progesterone dissolved in sesame oil (when first given at 2 h post CLP.) The calculated molar concentration of PRO (MW = 314.47 Daltons; 1.4 mg/kg) intraperitoneally injected into septic animals was estimated to be > 4.5 μM and comparable to the effective concentrations (10 µM) of PRO in inhibiting pCTS-L-induced dysregulated inflammation *in vitro* (**Figure 3B**).

To elucidate the underlying protective mechanisms of PRO, we assessed its impact on CLP sepsis-triggered inflammation and tissue injury. In agreement with our previous report (4), experimental sepsis markedly elevated blood levels of G-CSF, MIP-2 and sTNFR1 (**Figure 4B**). In contrast to DM-β-CD vehicle, which did not affect sepsis-triggered systemic accumulation of any cytokines or chemokines, PRO-carrying DM-β-CD nanoparticles significantly reduced sepsis-triggered elevation of MIP-2 and sTNFRI (**Figure 4B**). Furthermore, in contrast to DM-β-CD vehicle, which did not affect sepsis-triggered release of liver enzymes (such as AST) or other general tissue injury markers such as LDH, PRO-carrying DM-β-CD nanoparticles significantly reduced the levels of AST and LDH in septic animals (**Figure 4C**), suggesting that PRO-entrapping DM-β-CD nanoparticles protect mice against infections partly by suppressing sepsis-mediated dysregulated inflammation and tissue injury.

### PRO interacted and positively correlated with pCTS-L in clinical sepsis

**Figure 5:**
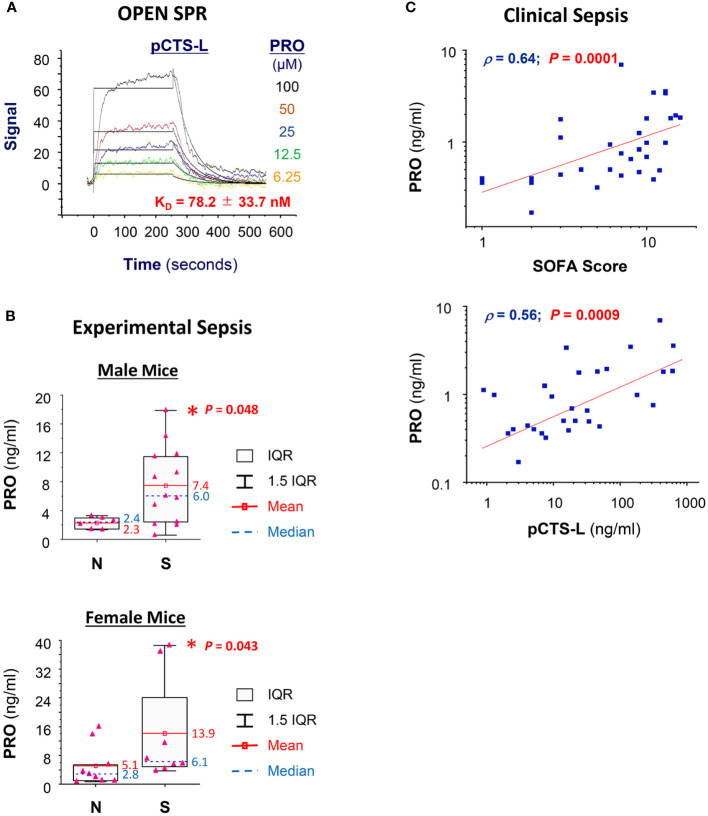
PRO interacted and positively correlated with pCTS-L in clinical sepsis. **(A)** SPR analysis of PRO/pCTS-L interaction. Recombinant pCTS-L with an N-terminal 6 × His tag was immobilized onto nitrilotriacetic acid (NTA)-conjugated chip, and an analyte of PRO solution (in DMSO) was injected at several increasing concentrations (6.25, 12.5, 25.0, 50.0 and 100.0 µM) to estimate the KD of PRO-pCTS-L interaction. PRO was injected for a contact time of 250 seconds at increasing concentrations, and dissociation was monitored for 300 seconds. The 1:1 model fit to the raw data is shown as solid black lines. The K_D_ was shown as the mean ± SEM of three independent experiments. **(B)** Elevation of serum PRO levels in septic mice at 24 h post CLP surgery. Male Balb/C mice were subjected to CLP surgery, and animals were sacrificed at 24 h post CLP to harvest blood and to compare serum PRO levels between normal healthy (“N”) and septic (“S”) mice. *, *P* < 0.05 versus normal healthy mice (N), non-parametric Kruskal-Wallis ANOVA test. **(C)** Correlation between serum PRO concentrations and sequential organ failure assessment (SOFA) score as well as serum pCTS-L levels in septic patients.

To gain further insight into PRO’s protective mechanisms, we examined the possible interaction and relationship between systemic accumulation PRO and pCTS-L in clinical sepsis. SPR analysis revealed a strong interaction between PRO and pCTS-L, as evidenced by the relatively low equilibrium dissociation constant (KD, **Figure 5A**) for PRO-pCTS-L interaction, suggesting that PRO might bind pCTS-L to inhibit its proinflammatory properties under pharmacological conditions. In agreement with previous findings of a marked (1 - 5 folds) elevation of blood PRO levels in septic animals (31) or patients with severe sepsis (32) or septic shock (33), we found a significant increase of serum PRO levels in septic mice (**Figure 5B**), as well as a parallel positive correlation with Sequential Organ Failure Assessment (SOFA, **Supplementary Table 1**) score and serum pCTS-L concentrations in patients with clinical sepsis (**Figure 5C**), confirming a possible progesterone upregulation as a potential protective mechanism against infections.

## Discussion

Currently, there is no effective therapies for clinical sepsis other than some adjunctive care such as administration of antibiotics and resuscitation of fluid (34–36). Therefore, it might still be important to explore small-molecule inhibitors to attenuate late-acting pathogenic mediators that may have relatively wider therapeutic windows (5). Here, we showed that a lipophilic hormone, PRO, selectively impaired pCTS-L-triggered production of several chemokines (e.g., ENA-78, MCP-1 or MCP-3) and cytokines (e.g., TNF, IL-6 or IL-10) without affecting LPS-induced TNF production. Our findings were in agreement with previous reports that PRO failed to inhibit LPS-stimulated TNF production in human PBMCs (37, 38). Nevertheless, it has been shown that PRO could inhibit LPS-stimulated production and secretion of TNF, IL-6, IL-12 or nitric oxide in other types of immune cells such as macrophages (39–43) and microglial cells (44, 45) potentially via PRO receptor-independent mechanisms (42). The discrepancies between divergent effects of progesterone on LPS-induced responses in different cell types remain a subject of future investigation. Nevertheless, as a steroid hormone produced primarily by the ovaries in women and, to a lesser extent, by the adrenal glands in both men and women, PRO occupies a crucial role in regulating menstrual cycles of the female reproductive system as well as innate immune responses against microbial infections.

Consequently, PRO-carrying DM-β-CD nanoparticles rescued both male and female mice from microbial infections even when they were initially given at 24 hours post the disease onset. This finding mirrored previous observations that systemic administration of PRO attenuated endotoxin-induced hypotension (46), CLP-induced systemic inflammation (47), as well as dysregulated inflammatory responses elicited by viral infections with SARS-CoV-2 (48, 49) and hepatitis C virus (50). When given locally, however, subcutaneous implanting PRO adversely thinned the cervicovaginal epithelium and adversely enhanced vaginal transmission of Simian immunodeficiency virus (SIV) (51), genital herpes infection (52) or HIV infection (53). It will thus be important to determine whether systemic administration of PRO at pharmacological doses will confer universal protection against all bacterial and viral infections in future preclinical and clinical investigations.

Because the lipophilic PRO is not soluble in water, we employed either sesame oil to emulsify it into micelles or DM-β-CD to complex with it to produce water-soluble PRO-carrying DM-β-CD-based nanoparticles. In the water solution, DM-β-CD displays the shape of truncated cone with a hydrophilic outer surface that renders it water-soluble and a hydrophobic inner cavity that entraps small hydrophobic molecules such as PRO (54, 55). This combined internal hydrophobic cavity along with external hydrophilicity enables DM-β-CD to form “host-guest” complexes with hydrophobic PRO via van der Waals forces and hydrophobic interactions (56). Although both a partial and a complete inclusion may occur, free-energy calculations favored the partial inclusion event for DM-β-CD and PRO (56), in which the complexation was initiated by inclusion of PRO into the first DM-β-CD to produce the 1:1 complex, but continued with the engagement of the second DM-β-CD to generate the more stable 1:2 complex (55, 57). Therefore, the hydrophobic part of PRO can be hidden within the inner hydrophobic cores of two DM-β-CD molecules, thereby potentially improving the solubility, stability, as well as bioavailability of the PRO *in vivo* (58). However, the entrapment of this hydrophobic PRO by DM-β-CD is reversible, enabling rapid dissociation of PRO from the complexes upon their dilution in biological fluids (58, 59). PRO was released from DM-β-CD within hours (59), and the dissociated PRO could potentially bind pCTS-L to interrupt some of its proinflammatory properties. The free DM-β-CDs could be gradually removed from the circulation within 36 h, destining to organs with increased blood flow velocities such as the spleen, liver, and kidney (60), where it can be metabolized (60) or excreted via glomerular filtration (61).

Regardless of whether PRO was solubilized in organic solvents (e.g., DMSO or ethanol) or entrapped in DM-β-CD-based nanoparticles, it similarly suppressed pCTS-L-induced secretion of several chemokines (e.g., ENA-78, MCP-1, GRO, or MCP-3) and cytokines (e.g., TNF or IL-10) in human PBMCs. The intricate mechanisms of PRO-mediated suppression of pCTS-L-stimulated inflammation will be an interesting subject for future investigations. In light of the essential involvement of TLR4 and RAGE in pCTS-L-induced inflammation (4), it will be interesting to determine whether PRO interacts with pCTS-L to interfere with its interaction with TLR4 or RAGE receptors. This is plausible, because pCTS-L-neutralizing antibodies inhibited pCTS-L-stimulated inflammation by disrupting pCTS-L interaction with PRRs such as TLR4 and RAGE (4).

To our best knowledge, the DM-β-CD-based nanoparticle technology has not yet been used to explore the therapeutic potential of PRO in any animal models of microbial infections. Consistent with its inhibitory activity in inhibiting late-acting mediator pCTS-L-induced cytokine/chemokine production in human PBMCs, we found that delayed administration of PRO-carrying DM-β-CD nanoparticles effectively rescued both male and female mice from microbial infections even when they were initially given at 24 h post onset of infections. Currently, the mechanism for PRO-mediated protection remains elusive, but appeared to attribute to its attenuation of sepsis- or pCTS-L-induced dysregulated inflammation and tissue injury. Indeed, PRO-carrying DM-β-CD nanoparticles significantly attenuated sepsis-triggered accumulation of G-CSF, sTNFRI and MIP-2/GRO-β, three pCTS-L-inducible surrogate markers of experimental sepsis (20, 62, 63). Our findings were in agreement with an earlier report (47), and support the possibility that PRO protects mice against sepsis partially by suppressing sepsis-triggered dysregulated inflammation. In light of the pathogenic involvement of pCTS-L in other inflammatory diseases such as pancreatitis (64), atherosclerosis (65), renal disease (66), vascular intimal hyperplasia (67), arthritis (68) and colitis (69), it will be interesting to explore the therapeutic potential of PRO-carrying DM-β-CD nanoparticles in other inflammatory diseases.

There are a few limitations in the current study: (i) We did not assess systemic inflammatory cytokine profiles at later stages of sepsis, because many septic animals in the control group might have succumbed to sepsis between 24 - 48 h post CLP (as depicted in **Figure 4A**), rendering blood sampling at later time points after the death of some septic animals in the control vehicle group practically infeasible. Even if post-mortem blood collection was still feasible, post-mortem tissue decomposition could lead to the release of cellular contents (including cytokines) into the surrounding environment, resulting in artificially elevated cytokine levels in post-mortem samples. Thus, sampling at later time points particularly after the death of septic animals in the control group might introduce confounding variables that hinder the interpretability of the systemic inflammatory profile, rendering them ineligible for the inclusion of comprehensive assessment of systemic inflammation at later points (e.g., 48 h post CLP). (ii) We do not know if PRO-entrapping DM-β-CD nanoparticles are orally active and protective against sepsis and other bacterial or viral infections. (iii) We do not know why progesterone selectively inhibits pCTS-L-mediated inflammation, although the robust interaction between progesterone and pCTS-L may contribute to the observed selectivity, allowing progesterone to inhibit pCTS-L-mediated inflammation without impacting LPS-mediated cytokine/chemokine production. (iv) It remains elusive why PRO-entrapping DM-β-CD nanoparticles exhibited a bell-shaped dose-response curve in inhibiting pCTS-L-mediated inflammatory response, although this type of bell-shaped dose-response curve has also been observed in the context of PRO-mediated protection against cerebral ischemic injury (70, 71), wherein a higher dose (32 mg/kg) exhibited reduced efficacy compared to lower doses (e.g., 8 mg/kg or 16 mg/kg). Bell-shaped concentration-response curves typically indicate more complex biological effects, such as receptor saturation or dual mechanisms of action with multiple binding sites or targets of DM-β-CD complexes. For instance, at extremely higher concentrations (5 - 50 mM), β-CD and derivatives (e.g., DM-β-CD) can extract lipophilic molecules such as cholesterol from cytoplasmic membranes (72, 73), thereby disrupting lipid rafts to interfere with innate immune responses to microbial infections (74, 75). Similarly, at extremely high concentrations (in the ranges of mM), another β-CD derivative, 2-Hydroxypropyl-β-cyclodextrin, also exhibited some pro-inflammatory properties as evidenced by the increased expression and release of cytokines (such as TNF and CCL2/MCP-1) by murine macrophages (75). At present, it is not yet known whether DM-β-CD possesses similar weak proinflammatory properties in complexation with PRO that could be attributed to the bell-shaped dose-response cure of PRO/DM-β-CD complexes. Despite these limitations of this study, our findings of PRO as a selective inhibitor of pCTS-L-mediated dysregulated inflammation have supported a promising opportunity of developing novel DM-β-CD-based nanoparticles to treat microbial infections. Therefore, it will be critical to further develop PRO-entrapping DM-β-CD-based nanoparticles and further translate our pre-clinical research into clinical treatment of bacterial infections.

## Data availability statement

The original contributions presented in the study are included in the article/**Supplementary Materials**, further inquiries can be directed to the corresponding author/s.

## Ethics statement

This study was administratively approved by the institutional review board (IRB) of the FIMR (IRB protocol #18-0184). The studies were conducted in accordance with the local legislation and institutional requirements. The participants provided their written informed consent to participate in this study. Our animal study was approved by the Institutional Animal Care and Use Committee (IACUC) of the FIMR (Protocol # 2017-003 Term II, approved on April 28th, 2020). The study was conducted in accordance with the local legislation and institutional requirements.

## Author contributions

XQ: Writing – review & editing, Methodology, Investigation, Formal analysis, Data curation. WC: Writing – review & editing, Methodology, Investigation, Data curation. CZ: Writing – review & editing, Methodology, Investigation, Data curation. JL: Writing – review & editing, Resources. TQ: Writing – review & editing, Investigation, Data curation. LL: Writing – review & editing, Investigation, Formal analysis. PW: Writing – review & editing, Conceptualization. KT: Writing – review & editing, Resources. HW: Writing – review & editing, Writing – original draft, Supervision, Project administration, Funding acquisition, Formal analysis, Conceptualization.
