# Supplementary material for: Therapeutic potential of procathepsin L-inhibiting and progesterone-entrapping dimethyl-β-cyclodextrin nanoparticles in treating experimental sepsis
Source: Front Immunol. 2024 Mar 14;15:1368448. doi: 10.3389/fimmu.2024.1368448 (PMC10972846; doi:10.3389/fimmu.2024.1368448)
Supplement: Supplementary file 1 [file DataSheet_1.docx]

Supplemental Information

Therapeutic potential of procathepsin L (pCTS-L)-hindering and progesterone-entrapping dimethyl-β-cyclodextrin nanoparticles in treating experimental sepsis.

Xiaoling Qiang ^1, 2#^, Weiqiang Chen ^1, 2#^, Cassie Shu Zhu ^1, 2#^, Jianhua Li ^1^, Timothy Qi ^1^, Li Lou ^1^, Ping Wang ^1, 2^, Kevin J. Tracey ^1, 2^, and Haichao Wang ^1, 2*^

#, Authors contributed equally

*, Correspondence: hwang@northwell.edu; Tel: +1-(516)562-2823

**Materials and Methods**

***Cell Mitochondrial Metabolisms Assay***

Cell mitochondrial metabolisms was assessed using MTT Assay (Cat. # 11465007001, Sigma-Aldrich) as per the manufacturer’s guidelines. This assay gauged cellular metabolic activity as an indicator of cell viability, proliferation and cytotoxicity by assessing the reduction of a yellow tetrazolium salt to purple formazan crystals in metabolically active cells. Following solubilization of the formazan crystals, the resulting-colored solution was quantified by measuring absorbance at 500-600 nanometers. Cell viability was expressed as a percentage of controls (in the presence of pCTS-L) based on the results of four independent experiments.

***Cell Viability Assay***

Cell viability was evaluated by the trypan blue exclusion method, which distinguished the unstained viable cells from nonviable cells that taken up the dye to exhibit a distinctive blue color. Phase contrast images of multiple fields were randomly captured, and the percentage of trypan blue-stained cells was calculated.  **Supplemental Figure 1**

**Figure 1S. Effect of progesterone on cell mitochondrial metabolism or cell viability in macrophage cultures.** Murine macrophage-like RAW 264.7 cells were cultured until 80-90% confluence and challenged with recombinant murine pCTS-L (1.0 µg/ml) in the absence or presence of progesterone (PRO) dissolved in DMSO at different concentrations for 16 h. The cell mitochondrial metabolism and cell viability were assessed by MTT and Trypan Blue Uptake Assay, respectively.

**Table 1. pCTS-L-inhibitory activities of 1360 Compounds in the U.S. Collection of Drugs**

| **NAME** | **Formula** | **Mol Weight** | **Known**  **Bioactivity** | **pCTS-L inhibitory activity** |
| --- | --- | --- | --- | --- |
| GRISEOFULVIN | C17H17ClO6 | 352.77444 | antifungal, inhibits mitosis in metaphase |  |
| SALSALATE | C14H10O5 | 258.2328 | analgesic |  |
| DANTHRON | C14H8O4 | 240.21746 | cathartic |  |
| MEQUINOL | C7H8O2 | 124.14061 | skin depigmentor |  |
| HYDROCORTISONE | C21H30O5 | 362.47025 | glucocorticoid, antiinflammatory |  |
| DESOXYCORTICOSTERONE ACETATE | C23H32O4 | 372.50909 | mineralocorticoid |  |
| TESTOSTERONE PROPIONATE | C22H32O3 | 344.49854 | androgen, antineoplastic |  |
| SPARTEINE SULFATE | C15H28N2O4S | 332.46541 | oxytocic |  |
| DELAPRIL HYDROCHLORIDE | C26H33ClN2O5 | 489.01631 | antihypertensive, ACE inhibitor |  |
| PHENYL AMINOSALICYLATE | C13H11NO3 | 229.23752 | antibacterial (tuberculostatic) |  |
| TESTOSTERONE | C19H28O2 | 288.43381 | androgen, antineoplastic |  |
| SANGUINARIUM CHLORIDE | C20H14ClNO4 | 367.79188 | antineoplastic, antiplaque agent |  |
| MITOMYCIN | C15H18N4O5 | 334.33451 | antineoplastic |  |
| SODIUM NITROPRUSSIDE DIHYDRATE | C5H4FeN6Na2O3 | 297.95263 | antihypertensive |  |
| SODIUM OXYBATE | C4H7NaO3 | 126.08839 | anesthetic |  |
| MANNITOL | C6H14O6 | 182.17488 | diuretic, sweetener, diagnostic aid |  |
| ACETAMINOPHEN | C8H9NO2 | 151.16643 | analgesic, antipyretic |  |
| ACETYLCHOLINE CHLORIDE | C7H16ClNO2 | 181.66407 | cholinergic, antiarrhythmic, miotic, vasodilator (peripheral) |  |
| ACETYLCYSTEINE | C5H9NO3S | 163.19638 | mucolytic |  |
| ADENOSINE | C10H13N5O4 | 267.24621 | antiarrhythmic, cardiac depressant |  |
| ALLOPURINOL | C5H4N4O | 136.11383 | antihyperuricemia, antigout, antiurolithic |  |
| ALVERINE CITRATE | C26H35NO7 | 473.57135 | anticholinergic |  |
| AMANTADINE HYDROCHLORIDE | C10H18ClN | 187.71466 | antiviral, antiparkinsonian; treatment of drug-induced extrapyrimidal reactions |  |
| LEVOMILNACIPRAN HYDROCHLORIDE | C15H23ClN2O | 282.81636 | antidepressant, seritonin & norepinephrine reuptake inhibitor |  |
| AMILORIDE HYDROCHLORIDE | C6H9Cl2N7O | 266.09093 | Na+ channel inhibitor, diuretic |  |
| POTASSIUM p-AMINOBENZOATE | C7H6KNO2 | 175.23337 | ultraviolet screen |  |
| AMINOCAPROIC ACID HYDROCHLORIDE | C6H14ClNO2 | 167.63698 | hemostatic |  |
| PRASUGREL | C20H20FNO3S | 373.4497 | platelet aggregation inhibitor |  |
| AMINOSALICYLATE SODIUM | C7H6NNaO3 | 175.12057 | antibacterial, tuberculostatic |  |
| AMITRIPTYLINE HYDROCHLORIDE | C20H24ClN | 313.87398 | antidepressant |  |
| AMODIAQUINE DIHYDROCHLORIDE | C20H24Cl3N3O | 428.79278 | antimalarial |  |
| AMOXICILLIN | C16H19N3O5S | 365.41093 | antibacterial |  |
| AMPHOTERICIN B | C47H73NO17 | 924.10236 | antifungal |  |
| AMPICILLIN SODIUM | C16H18N3NaO4S | 371.39336 | antibacterial |  |
| ANTHRALIN | C14H10O3 | 226.234 | antipsoriatic |  |
| ANTIPYRINE | C11H12N2O | 188.23109 | analgesic |  |
| APOMORPHINE HYDROCHLORIDE | C17H18ClNO2 | 303.79151 | dopamine agonist, antiparkinsonian, erextile dysfunction therapy |  |
| ASPIRIN | C9H8O4 | 180.16171 | analgesic, antipyretic, antiinflammatory |  |
| ATROPINE SULFATE | C17H25NO7S | 387.4553 | anticholinergic, mydriatic |  |
| AZATHIOPRINE | C9H7N7O2S | 277.26584 | immunosuppressant, antineoplastic, antirheumatic |  |
| BACITRACIN | C66H103N17O16S | 1422.72511 | antibacterial |  |
| BECLOMETHASONE DIPROPIONATE | C28H37ClO7 | 521.05589 | antiasthmatic, topical antiinflammatory |  |
| BENSERAZIDE HYDROCHLORIDE | C10H16ClN3O5 | 293.70912 | decarboxylase inhibitor |  |
| BENZETHONIUM CHLORIDE | C27H42ClNO2 | 448.09429 | antiinfective (topical) |  |
| BENZOCAINE | C9H11NO2 | 165.19352 | anesthetic (topical) |  |
| BENZTHIAZIDE | C15H14ClN3O4S3 | 431.94153 | diuretic, antihypertensive |  |
| CARBOPLATIN | C6H12N2O4Pt | 371.26354 | antineoplastic, convulsant |  |
| HEPARIN SODIUM | C19H30NO22S2 | 688.57245 | anticoagulant |  |
| BETHANECHOL CHLORIDE | C7H17ClN2O2 | 196.67874 | cholinergic |  |
| BISACODYL | C22H19NO4 | 361.40103 | cathartic |  |
| BITHIONOL | C12H6Cl4O2S | 356.05642 | anthelmintic, antiseptic |  |
| BROMOCRIPTINE MESYLATE | C33H44BrN5O8S | 750.71533 | prolactin inhibitor, antiparkinsonian |  |
| BUSULFAN | C6H14O6S2 | 246.30288 | antineoplastic, alkylating agent |  |
| CAFFEINE | C8H10N4O2 | 194.1945 | CNS stimulant |  |
| CAMPHOR | C10H16O | 152.23842 | analgesic, antiinfective, antipruritic |  |
| CARBACHOL | C6H15ClN2O2 | 182.65165 | cholinergic, miotic |  |
| CARBAMAZEPINE | C15H12N2O | 236.27569 | analgesic, anticonvulsant |  |
| CARBENICILLIN DISODIUM | C17H16N2Na2O6S | 422.37047 | antibacterial |  |
| CARBINOXAMINE MALEATE | C20H23ClN2O5 | 406.86971 | antihistaminic |  |
| CARISOPRODOL | C12H24N2O4 | 260.33608 | muscle relaxant (skeletal) |  |
| CEFADROXIL | C16H17N3O5S | 363.39499 | antibacterial |  |
| CEFAZOLIN SODIUM | C14H13N8NaO4S3 | 476.49271 | antibacterial |  |
| CEFOTAXIME SODIUM | C16H16N5NaO7S2 | 477.45302 | antibacterial |  |
| FIROCOXIB | C17H20O5S | 336.40995 | analgesic, antiinflammatory, antipyretic, COX-II inhibitor |  |
| CEPHAPIRIN SODIUM | C17H16N3NaO6S2 | 445.45137 | antibacterial |  |
| ACETAZOLAMIDE | C4H6N4O3S2 | 222.24542 | carbonic anhydrase inhibitor, diuretic, antiglaucoma |  |
| CEPHRADINE | C16H19N3O4S | 349.41153 | antibacterial |  |
| CETYLPYRIDINIUM CHLORIDE | C21H38ClN | 339.99671 | antiinfective (topical) |  |
| CHLORAMBUCIL | C14H19Cl2NO2 | 304.21903 | antineoplastic, alkylating agent |  |
| CHLORAMPHENICOL PALMITATE | C27H42Cl2N2O6 | 561.55159 | antibacterial, antirickettsial |  |
| CHLORAMPHENICOL SODIUM SUCCINATE | C15H15Cl2N2NaO8 | 445.1912 | antibacterial, antirickettsial, inhibits protein synthesis |  |
| CHLORAMPHENICOL | C11H12Cl2N2O5 | 323.13469 | antibacterial, antirickettsial, inhibits protein synthesis |  |
| NORGESTIMATE | C23H31NO3 | 369.50842 | progestin |  |
| CHLORHEXIDINE DIHYDROCHLORIDE | C22H32Cl4N10 | 578.37934 | antibacterial (topical), disinfectant |  |
| CHLOROCRESOL | C7H7ClO | 142.58624 | antiinfectant |  |
| CHLOROTHIAZIDE | C7H6ClN3O4S2 | 295.72457 | diuretic, antihypertensive |  |
| CHLOROXYLENOL | C8H9ClO | 156.61333 | antibacterial, topical and urinary antiseptic |  |
| DEXCHLORPHENIRAMINE MALEATE | C20H23ClN2O4 | 390.87031 | antihistamine |  |
| CHLORPROMAZINE HYDROCHLORIDE | C17H20Cl2N2S | 355.33235 | antiemetic, antipsychotic |  |
| CHLORPROPAMIDE | C10H13ClN2O3S | 276.74371 | antidiabetic |  |
| CHLORTETRACYCLINE HYDROCHLORIDE | C22H24Cl2N2O8 | 515.35118 | antibacterial, antiamebic, Ca chelator, hepatotoxic; inhibits protein synthesis |  |
| CHLORTHALIDONE | C14H11ClN2O4S | 338.77177 | diuretic, antihypertensive |  |
| CHLORZOXAZONE | C7H4ClNO2 | 169.56843 | muscle relaxant (skeletal) |  |
| CICLOPIROX OLAMINE | C14H24N2O3 | 268.35898 | antifungal |  |
| CINOXACIN | C12H10N2O5 | 262.2239 | antibacterial |  |
| CLEMASTINE FUMARATE | C25H30ClNO5 | 459.97455 | antihistaminic |  |
| CLIDINIUM BROMIDE | C22H26BrNO3 | 432.36142 | anticholinergic |  |
| CLINDAMYCIN HYDROCHLORIDE | C18H34Cl2N2O5S | 461.45208 | antibacterial, inhibits protein synthesis |  |
| CLOMIPHENE CITRATE | C32H36ClNO8 | 598.09862 | gonad stimulating principle |  |
| CLONIDINE HYDROCHLORIDE | C9H10Cl3N3 | 266.55915 | antihypertensive |  |
| CLOTRIMAZOLE | C22H17ClN2 | 344.84719 | antifungal |  |
| CLOXACILLIN SODIUM | C19H17ClN3NaO5S | 457.87124 | antibacterial |  |
| CLOXYQUIN | C9H6ClNO | 179.60727 | antibacterial, antifungal |  |
| COLCHICINE | C22H25NO6 | 399.44765 | antimitotic, antigout agent |  |
| COLISTIMETHATE SODIUM | C57H103N16Na5O28S5 | 1735.81586 | antibacterial |  |
| CORTISONE ACETATE | C23H30O6 | 402.49195 | glucocorticoid |  |
| BAZEDOXIFENE ACETATE | C32H38N2O5 | 530.67006 | antiosteoporotic |  |
| CRESOL | C7H8O | 108.14121 | antiinfectant |  |
| CROMOLYN SODIUM | C23H14Na2O11 | 512.34103 | antiasthmatic, antiallergy |  |
| CYCLIZINE | C18H22N2 | 266.38944 | H1 antihistamine |  |
| CYCLOPENTOLATE HYDROCHLORIDE | C17H26ClNO3 | 327.85467 | mydriatic |  |
| CYCLOPHOSPHAMIDE | C7H17Cl2N2O3P | 279.10494 | antineoplastic, alkylating agent |  |
| CYCLOSERINE (D) | C3H6N2O2 | 102.09347 | antibacterial (tuberculostatic) |  |
| CYPROTERONE ACETATE | C24H29ClO4 | 416.94933 | antiandrogen |  |
| CYTARABINE | C9H13N3O5 | 243.22106 | antineoplastic, antiviral, antimetabolite |  |
| DACARBAZINE | C6H10N6O | 182.1862 | antineoplastic |  |
| DANAZOL | C22H27NO2 | 337.46599 | anterior pituitary suppressant |  |
| DAPSONE | C12H12N2O2S | 248.30564 | antibacterial, leprostatic, dermatitis herpetiformis suppressant |  |
| DAUNORUBICIN HYDROCHLORIDE | C27H30ClNO10 | 563.99385 | antineoplastic |  |
| DEFEROXAMINE MESYLATE | C26H52N6O11S | 656.80194 | chelating agent (Fe & Al) |  |
| DEHYDROCHOLATE SODIUM | C24H33NaO5 | 424.51741 | choleretic |  |
| DEMECLOCYCLINE HYDROCHLORIDE | C21H22Cl2N2O8 | 501.32409 | antibacterial |  |
| DESIPRAMINE HYDROCHLORIDE | C18H23ClN2 | 302.85041 | antidepressant |  |
| DEXAMETHASONE | C22H29FO5 | 392.47183 | glucocorticoid |  |
| DEXAMETHASONE ACETATE | C24H31FO6 | 434.50947 | glucocorticoid, antiinflammatory |  |
| DEXAMETHASONE SODIUM PHOSPHATE | C22H28FNa2O8P | 516.41546 | glucocorticoid, antiinflammatory |  |
| DEXTROMETHORPHAN HYDROBROMIDE | C18H26BrNO | 352.31802 | antitussive |  |
| DIBENZOTHIOPHENE | C12H8S | 184.26156 | keratolytic |  |
| DIBUCAINE HYDROCHLORIDE | C20H30ClN3O2 | 379.934 | anesthetic (local) |  |
| DICLOFENAC SODIUM | C14H10Cl2NNaO2 | 318.1371 | antiinflammatory |  |
| DICLOXACILLIN SODIUM | C19H16Cl2N3NaO5S | 492.31627 | antibacterial |  |
| DICYCLOMINE HYDROCHLORIDE | C19H36ClNO2 | 345.95727 | anticholinergic |  |
| DIENESTROL | C18H18O2 | 266.34296 | estrogen |  |
| DIETHYLCARBAMAZINE CITRATE | C16H29N3O8 | 391.42483 | anthelmintic |  |
| DIETHYLSTILBESTROL | C18H20O2 | 268.3589 | estrogen |  |
| DIFLUNISAL | C13H8F2O3 | 250.20371 | analgesic, antiinflammatory |  |
| DIGITOXIN | C41H64O13 | 764.95943 | inotropic, cardiotonic |  |
| DIGOXIN | C41H64O14 | 780.95883 | cardiac stimulant |  |
| DIHYDROERGOTAMINE MESYLATE | C34H41N5O8S | 679.79857 | vasoconstrictor, antimigraine |  |
| DIMENHYDRINATE | C24H28ClN5O3 | 469.97546 | antiemetic |  |
| DIMERCAPROL | C3H8OS2 | 124.22461 | chelating agent (As, Au, Hg antidote) |  |
| DIMETHADIONE | C5H7NO3 | 129.11644 | anticonvulsant |  |
| DIOXYBENZONE | C14H12O4 | 244.24934 | ultraviolet screen |  |
| DIPHENHYDRAMINE HYDROCHLORIDE | C17H22ClNO | 291.82399 | antihistaminic |  |
| DIPHENYLPYRALINE HYDROCHLORIDE | C19H24ClNO | 317.86223 | antihistaminic |  |
| DIPYRIDAMOLE | C24H40N8O4 | 504.6376 | coronary vasodilator |  |
| PYRITHIONE ZINC | C10H8N2O2S2Zn | 317.68546 | antibacterial, antifungal, antiseborrheic |  |
| DISULFIRAM | C10H20N2S4 | 296.5403 | alcohol antagonist |  |
| DOPAMINE HYDROCHLORIDE | C8H12ClNO2 | 189.64334 | cardiotonic, antihypotensive |  |
| DOXEPIN HYDROCHLORIDE | C19H22ClNO | 315.84629 | antidepressant |  |
| DOXYCYCLINE HYDROCHLORIDE | C22H25ClN2O8 | 480.90615 | antibacterial |  |
| DOXYLAMINE SUCCINATE | C21H28N2O5 | 388.46771 | antihistaminic, hypnotic |  |
| DYCLONINE HYDROCHLORIDE | C18H28ClNO2 | 325.88236 | anesthetic (topical) |  |
| DYPHYLLINE | C10H14N4O4 | 254.24748 | PDE inhibitor, bronchodilator, vasodilator |  |
| ETHYLENEDIAMINE TETRACETIC ACID | C10H16N2O8 | 292.24762 | chelating agent, antioxidant |  |
| EMETINE DIHYDROCHLORIDE | C29H42Cl2N2O4 | 553.57509 | inhibits RNA, DNA and protein synthesis |  |
| EPHEDRINE (1R,2S) HYDROCHLORIDE | C10H16ClNO | 201.69812 | bronchodilator, cardiac stimulant |  |
| EPINEPHRINE BITARTRATE | C13H19NO9 | 333.29768 | adrenergic agonist, bronchodilator, antiglaucoma agent |  |
| DOMPERIDONE | C22H24ClN5O2 | 425.92188 | antiemetic, dopamine antagonist |  |
| ERGOCALCIFEROL | C28H44O | 396.66228 | antirachitic vitamin | **IC50 = 2.0 µM** |
| SULFANILAMIDE | C6H8N2O2S | 172.20686 | antibacterial |  |
| ERYTHROMYCIN ETHYLSUCCINATE | C43H75NO16 | 862.0743 | antibacterial |  |
| ERYTHROMYCIN | C37H67NO13 | 733.94544 | antibacterial |  |
| ESTRADIOL | C18H24O2 | 272.39078 | estrogen |  |
| ESTRADIOL CYPIONATE | C26H36O3 | 396.57502 | estrogen |  |
| ESTRADIOL VALERATE | C23H32O3 | 356.50969 | estrogen |  |
| ESTRIOL | C18H24O3 | 288.39018 | estrogen |  |
| ESTRONE | C18H22O2 | 270.37484 | estrogen |  |
| ETHACRYNIC ACID | C13H12Cl2O4 | 303.14419 | diuretic |  |
| ETHAMBUTOL HYDROCHLORIDE | C10H26Cl2N2O2 | 277.23692 | antibacterial (tuberculostatic) |  |
| ETHINYL ESTRADIOL | C20H24O2 | 296.41308 | estrogen, plus progestogen as oral contraceptive |  |
| ETHIONAMIDE | C8H10N2S | 166.2463 | antibacterial, tuberculostatic |  |
| ETHOPROPAZINE HYDROCHLORIDE | C19H25ClN2S | 348.9415 | antiparkinsonian, anticholinergic |  |
| EUCALYPTOL | C10H18O | 154.25436 | anthelmintic, antiseptic, expectorant |  |
| PROTAMINE SULFATE [10mg/ ml] |  |  | heparin antidote |  |
| EUGENOL | C10H12O2 | 164.20594 | analgesic (topical), antiseptic, antifungal |  |
| FLUDROCORTISONE ACETATE | C23H31FO6 | 422.49832 | mineralocorticoid |  |
| FLUMETHAZONE PIVALATE | C27H36F2O6 | 494.58117 | glucocorticoid, antiinflammatory |  |
| FLUOCINOLONE ACETONIDE | C24H30F2O6 | 452.4999 | glucocorticoid, antiinflammatory |  |
| FLUOCINONIDE | C26H32F2O7 | 494.53754 | antiinflammatory, glucocorticoid |  |
| DORAMECTIN | C50H74O14 | 899.13888 | endoparasitic |  |
| CARTEOLOL HYDROCHLORIDE | C16H25ClN2O3 | 328.84225 | beta-adrenergic blocker |  |
| FLURBIPROFEN | C15H13FO2 | 244.26806 | antiinflammatory, analgesic |  |
| FURAZOLIDONE | C8H7N3O5 | 225.16209 | antibacterial |  |
| FUROSEMIDE | C12H11ClN2O5S | 330.74887 | diuretic, antihypertensive |  |
| FUSIDIC ACID | C31H48O6 | 516.72461 | antibacterial |  |
| GALLAMINE TRIETHIODIDE | C30H60I3N3O3 | 891.5442 | muscle relaxant (skeletal) |  |
| GEMFIBROZIL | C15H22O3 | 250.34079 | antihyperlipoproteinemic |  |
| GENTIAN VIOLET | C25H30ClN3 | 407.99095 | antibacterial, anthelmintic |  |
| GLUCOSAMINE HYDROCHLORIDE | C6H14ClNO5 | 215.63518 | antiarthritic |  |
| GRAMICIDIN (gramicidin A shown) | C99H140N20O17 | 1882.34345 | antibacterial |  |
| GUAIFENESIN | C10H14O4 | 198.22068 | expectorant |  |
| GUANABENZ ACETATE | C10H12Cl2N4O2 | 291.13874 | antihypertensive |  |
| HALAZONE | C7H5Cl2NO4S | 270.0922 | antiinfectant |  |
| HALOPERIDOL | C21H23ClFNO2 | 375.87436 | antidyskinetic, antipsychotic |  |
| HETACILLIN POTASSIUM | C19H22KN3O4S | 427.57089 | antibacterial |  |
| HEXACHLOROPHENE | C13H6Cl6O2 | 406.90957 | antiinfective (topical) |  |
| HEXYLRESORCINOL | C12H18O2 | 194.27606 | anthelmintic, topical antiseptic |  |
| HISTAMINE DIHYDROCHLORIDE | C5H11Cl2N3 | 184.06952 | H1&2 agonist, edema induction, gastric secretion stimulant |  |
| HOMATROPINE HYDROBROMIDE | C16H22BrNO3 | 356.26264 | anticholinergic (opthalmic) |  |
| HOMATROPINE METHYLBROMIDE | C17H24BrNO3 | 370.28973 | anticholinergic (opthalmic) |  |
| HYDROCHLOROTHIAZIDE | C7H8ClN3O4S2 | 297.74051 | diuretic |  |
| HYDROCORTISONE ACETATE | C23H32O6 | 404.50789 | glucocorticoid, antiinflammatory |  |
| HYDROCORTISONE HEMISUCCINATE | C25H34O8 | 462.54493 | glucocorticoid |  |
| HYDROCORTISONE PHOSPHATE TRIETHYLAMINE | C33H61N2O8P | 644.83652 | glucocorticoid |  |
| HYDROFLUMETHIAZIDE | C8H8F3N3O4S2 | 331.29386 | antihypertensive, diuretic |  |
| HYDROXYPROGESTERONE CAPROATE | C27H40O4 | 428.61745 | progestogen |  |
| HYDROXYUREA | CH4N2O2 | 76.05523 | antineoplastic, inhibits ribonucleoside diphosphate reductase |  |
| HYDROXYZINE PAMOATE | C44H43ClN2O8 | 763.29491 | anxiolytic, antihistaminic |  |
| HYOSCYAMINE | C17H23NO3 | 289.37776 | anticholinergic, analgesic |  |
| IBUPROFEN | C13H18O2 | 206.28721 | antiinflammatory |  |
| IMIPRAMINE HYDROCHLORIDE | C19H25ClN2 | 316.8775 | antidepressant |  |
| INDAPAMIDE | C16H16ClN3O3S | 365.84122 | diuretic, antihypertensive |  |
| INDOMETHACIN | C19H16ClNO4 | 357.79667 | antiinflammatory, antipyretic, analgesic |  |
| CANAGLIFLOZIN | C24H25FO5S | 444.52625 | antidiabetic |  |
| VANILLIN | C8H8O3 | 152.15116 | flavoring agent |  |
| ROBENIDINE HYDROCHLORIDE | C15H14Cl3N5 | 370.67133 | coccidiostat |  |
| IPRATROPIUM BROMIDE | C20H30BrNO3 | 412.371 | bronchodilator, antiarrhythmic |  |
| ISONIAZID | C6H7N3O | 137.14219 | antibacterial, tuberculostatic |  |
| ISOPROPAMIDE IODIDE | C23H33IN2O | 480.43666 | anticholinergic |  |
| ISOPROTERENOL HYDROCHLORIDE | C11H18ClNO3 | 247.72401 | bronchodilator |  |
| ISOSORBIDE DINITRATE | C6H8N2O8 | 236.13926 | antianginal |  |
| ISOXSUPRINE HYDROCHLORIDE | C18H24ClNO3 | 337.84988 | vasodilator |  |
| KANAMYCIN A SULFATE | C18H38N4O15S | 582.58536 | antibacterial |  |
| KETOCONAZOLE | C26H28Cl2N4O4 | 531.44346 | antifungal, PXR/SRC1 & CAR/SRC1 inhibitor |  |
| LACTULOSE | C12H22O11 | 342.30254 | laxative |  |
| LEUCOVORIN CALCIUM | C20H21CaN7O7 | 511.51307 | antianemic, antidote to folic acid antagonists |  |
| LEVONORDEFRIN | C9H13NO3 | 183.20886 | vasoconstrictor |  |
| LINCOMYCIN HYDROCHLORIDE | C18H35ClN2O6S | 443.00645 | antibacterial |  |
| MAFENIDE HYDROCHLORIDE | C7H11ClN2O2S | 222.69492 | antibacterial |  |
| MAPROTILINE HYDROCHLORIDE | C20H24ClN | 313.87398 | antidepressant |  |
| MECAMYLAMINE HYDROCHLORIDE | C11H22ClN | 203.75769 | antihypertensive |  |
| MECHLORETHAMINE | C5H11Cl2N | 156.05612 | antineoplastic, alkylating agent |  |
| MECLIZINE HYDROCHLORIDE | C25H29Cl3N2 | 463.88228 | antiemetic |  |
| MECLOFENAMATE SODIUM | C14H10Cl2NNaO2 | 318.1371 | antiinflammatory, antipyretic |  |
| MEDROXYPROGESTERONE ACETATE | C24H34O4 | 386.53618 | contraceptive | IC50 = 20 µM |
| MEDRYSONE | C22H32O3 | 344.49854 | glucocorticoid |  |
| MEGESTROL ACETATE | C24H32O4 | 384.52024 | progestogen, antineoplastic |  |
| MELPHALAN | C13H18Cl2N2O2 | 305.20661 | antineoplastic, alkylating agent |  |
| MEPENZOLATE BROMIDE | C21H26BrNO3 | 420.35027 | anticholinergic |  |
| MERCAPTOPURINE | C5H4N4S | 152.17843 | antineoplastic, purine antimetabolite |  |
| MESTRANOL | C21H26O2 | 310.44017 | estrogen, with progesterone as oral contraceptive |  |
| METAPROTERENOL | C11H17NO3 | 211.26304 | bronchodilator |  |
| METHACHOLINE CHLORIDE | C8H18ClNO2 | 195.69116 | cholinergic, diagnostic aid |  |
| METHENAMINE | C6H12N4 | 140.18934 | antibacterial (urinary) |  |
| METHICILLIN SODIUM | C17H19N2NaO6S | 402.40458 | antibacterial |  |
| METHIMAZOLE | C4H6N2S | 114.16982 | antihyperthyroid |  |
| METHOCARBAMOL | C11H15NO5 | 241.2459 | muscle relaxant (skeletal) |  |
| METHOTREXATE HYDRATE | C20H24N8O6 | 472.46428 | antineoplastic, antirheumatic, folic acid antagonist |  |
| METHOXAMINE HYDROCHLORIDE | C11H18ClNO3 | 247.72401 | alpha1 adrenoreceptor agonist, vasoconstrictor |  |
| METHOXSALEN | C12H8O4 | 216.19516 | antipsoriatic, pigmentation agent |  |
| METHSCOPOLAMINE BROMIDE | C18H24BrNO4 | 398.30028 | anticholinergic |  |
| METHYLDOPA | C10H13NO4 | 211.21941 | antihypertensive |  |
| DEFERASIROX | C21H15N3O4 | 373.3714 | chelating agent, Fe chelator |  |
| METHYLPREDNISOLONE | C22H30O5 | 374.4814 | glucocorticoid |  |
| METHYLTHIOURACIL | C5H6N2OS | 142.18037 | anticonvulsant, antithyroid agent |  |
| METOCLOPRAMIDE HYDROCHLORIDE | C14H23Cl2N3O2 | 336.26431 | antiemetic |  |
| METOPROLOL TARTRATE | C19H31NO9 | 417.46022 | antihypertensive, antianginal |  |
| METRONIDAZOLE | C6H9N3O3 | 171.15693 | antiprotozoal |  |
| MICONAZOLE NITRATE | C18H15Cl4N3O4 | 479.14995 | antifungal (topical) |  |
| MINOCYCLINE HYDROCHLORIDE | C23H28ClN3O7 | 493.94851 | antibacterial |  |
| MOXALACTAM DISODIUM | C20H18N6Na2O9S | 564.44486 | antibacterial |  |
| NADIDE | C21H27N7O14P2 | 663.43544 | alcohol and narcotic antagonist |  |
| NAFCILLIN SODIUM | C21H21N2NaO5S | 436.46572 | antibacterial |  |
| PHENOXYBENZAMINE HYDROCHLORIDE | C18H23Cl2NO | 340.29611 | alpha adrenergic blocker |  |
| NAPHAZOLINE HYDROCHLORIDE | C14H15ClN2 | 246.74205 | adrenergic agonist, nasal decongestant |  |
| NAPROXEN | C14H14O3 | 230.26588 | antiinflammatory, analgesic, antipyretic |  |
| NEOMYCIN TRISULFATE | C23H52N6O25S3 | 908.88809 | antibacterial |  |
| NEOSTIGMINE BROMIDE | C12H19BrN2O2 | 303.20143 | cholinergic |  |
| NIACIN | C6H5NO2 | 123.11225 | antihyperlipidemic, vitamin (enzyme cofactor) |  |
| NIFEDIPINE | C17H18N2O6 | 346.34281 | antianginal, antihypertensive |  |
| NITROFURANTOIN | C8H6N4O5 | 238.16082 | antibacterial |  |
| NITROFURAZONE | C6H6N4O4 | 198.13912 | antiinfective (topical) |  |
| NITROMIDE | C7H5N3O5 | 211.135 | antibacterial, coccidiostat |  |
| NOREPINEPHRINE TARTRATE | C12H17NO9 | 319.27059 | adrenergic agonist, antihypotensive |  |
| NORETHINDRONE | C20H26O2 | 298.42902 | progestogen |  |
| NORETHINDRONE ACETATE | C22H28O3 | 340.46666 | Oral contraceptive (in combination with estrogen) |  |
| NORTRIPTYLINE HYDROCHLORIDE | C19H22ClN | 299.84689 | antidepressant |  |
| NORETHYNODREL | C20H26O2 | 298.42902 | progestogen, in combination with estrogen as oral contraceptive |  |
| NORFLOXACIN | C16H18FN3O3 | 319.33856 | antibacterial |  |
| NORGESTREL | C21H28O2 | 312.45611 | progestogen |  |
| NOSCAPINE HYDROCHLORIDE | C22H24ClNO7 | 449.89208 | antitussive |  |
| NOVOBIOCIN SODIUM | C31H35N2NaO11 | 634.6212 | antibacterial |  |
| NYLIDRIN HYDROCHLORIDE | C19H26ClNO2 | 335.87757 | vasodilator (peripheral) |  |
| NYSTATIN | C47H75NO17 | 926.1183 | antifungal, binds to membrane sterols |  |
| ORPHENADRINE CITRATE | C24H31NO8 | 461.51657 | muscle relaxant (skeletal), antihistaminic |  |
| OXACILLIN SODIUM | C19H18N3NaO5S | 423.42621 | antibacterial |  |
| OXIDOPAMINE HYDROCHLORIDE | C8H12ClNO3 | 205.64274 | adrenergic agonist (opthalmic) |  |
| OXYBENZONE | C14H12O3 | 228.24994 | ultraviolet screen |  |
| OXYMETAZOLINE HYDROCHLORIDE | C16H25ClN2O | 296.84345 | adrenergic agonist, nasal decongestant |  |
| OXYPHENBUTAZONE | C19H20N2O3 | 324.38285 | antiinflammatory |  |
| OXYQUINOLINE HEMISULFATE | C9H9NO5S | 243.23978 | antiinfectant, complexing agent |  |
| OXYTETRACYCLINE | C22H25ClN2O9 | 496.90555 | antibacterial |  |
| PAPAVERINE HYDROCHLORIDE | C20H22ClNO4 | 375.85564 | muscle relaxant (smooth), cerebral vasodilator |  |
| PARACHLOROPHENOL | C6H5ClO | 128.55915 | topical antibacterial (topical) |  |
| PARGYLINE HYDROCHLORIDE | C11H14ClN | 195.69393 | antihypertensive |  |
| PENICILLIN G POTASSIUM | C16H17KN2O4S | 372.49089 | antibacterial |  |
| PENICILLIN V POTASSIUM | C16H17KN2O5S | 388.49029 | antibacterial |  |
| PHENACEMIDE | C9H10N2O2 | 178.19225 | anticonvulsant |  |
| PHENAZOPYRIDINE HYDROCHLORIDE | C11H12ClN5 | 249.70479 | analgesic |  |
| PHENELZINE SULFATE | C8H14N2O4S | 234.27578 | antidepressant |  |
| PHENINDIONE | C15H10O2 | 222.24575 | anticoagulant |  |
| PHENIRAMINE MALEATE | C20H24N2O4 | 356.42528 | antihistaminic |  |
| PHENOLPHTHALEIN | C20H14O4 | 318.33218 | cathartic, laxative |  |
| PHENYLBUTAZONE | C19H20N2O2 | 308.38345 | antiinflammatory |  |
| L-PHENYLEPHRINE HYDROCHLORIDE | C9H14ClNO2 | 203.67043 | mydriatic, decongestant |  |
| 1S,2R-PHENYLPROPANOLAMINE HYDROCHLORIDE | C9H14ClNO | 187.67103 | vasoconstrictor, decongestant, anorexic |  |
| PHENYTOIN SODIUM | C15H11N2NaO2 | 274.25692 | anticonvulsant, antieleptic |  |
| APIXABAN | C25H25N5O4 | 459.5091 | factor Xa inhibitor, anticoagulant, antithrombotic |  |
| PILOCARPINE NITRATE | C11H17N3O5 | 271.27524 | antiglaucoma agent, miotic |  |
| PINDOLOL | C14H20N2O2 | 248.3277 | antihypertensive, antianginal, antiarrhythmic, antiglaucoma |  |
| PIPERACILLIN SODIUM | C23H26N5NaO7S | 539.54677 | antibacterial |  |
| PIPERAZINE | C4H10N2 | 86.1377 | anthelmintic |  |
| PIROXICAM | C15H13N3O4S | 331.35256 | antiinflammatory |  |
| POLYMYXIN B SULFATE | C56H100N16O17S | 1301.5824 | antibacterial |  |
| PRAZIQUANTEL | C19H24N2O2 | 312.41533 | anthelmintic |  |
| KITASAMYCINS [A1 shown] | C40H67NO14 | 785.97829 | antibacterial |  |
| PREDNISOLONE | C21H28O5 | 360.45431 | glucocorticoid |  |
| PREDNISOLONE ACETATE | C23H30O6 | 402.49195 | glucocorticoid |  |
| PREDNISONE | C21H26O5 | 358.43837 | glucocorticoid |  |
| PRIMAQUINE PHOSPHATE | C15H27N3O9P2 | 455.34474 | antimalarial |  |
| PRIMIDONE | C12H14N2O2 | 218.25758 | anticonvulsant |  |
| PROBENECID | C13H19NO4S | 285.36468 | uricosuric |  |
| PROCAINAMIDE HYDROCHLORIDE | C13H22ClN3O | 271.79279 | antiarrhythmic |  |
| PROCAINE HYDROCHLORIDE | C13H21ClN2O2 | 272.77752 | anesthetic (local) |  |
| PROCHLORPERAZINE EDISYLATE | C22H30ClN3O6S3 | 564.1459 | antiemetic, antipsychotic, treatment of vertigo |  |
| PROCYCLIDINE HYDROCHLORIDE | C19H30ClNO | 323.91005 | anticholinergic |  |
| PROGESTERONE | C21H30O2 | 314.47205 | progestogen | IC50 = 20 µM |
| PROMAZINE HYDROCHLORIDE | C17H21ClN2S | 320.88732 | antipsychotic |  |
| PROMETHAZINE HYDROCHLORIDE | C17H21ClN2S | 320.88732 | antihistaminic |  |
| PROPANTHELINE BROMIDE | C23H30BrNO3 | 448.40445 | anticholinergic |  |
| DEXPROPRANOLOL HYDROCHLORIDE [R(+)] | C16H22ClNO2 | 295.81224 | antihypertensive, antianginal, antiarrhythmic |  |
| PROPYLTHIOURACIL | C7H10N2OS | 170.23455 | antihyperthyroid |  |
| beta-CAROTENE [2mM] | C40H56 | 536.89232 | antioxidant; provitamin A |  |
| PYRANTEL PAMOATE | C34H30N2O6S | 594.692 | anthelmintic |  |
| PYRAZINAMIDE | C5H5N3O | 123.1151 | antibacterial, tuberculostatic |  |
| PYRILAMINE MALEATE | C21H27N3O5 | 401.46644 | antihistaminic |  |
| PYRIMETHAMINE | C12H13ClN4 | 248.71721 | antimalarial |  |
| PYRVINIUM PAMOATE | C49H43N3O6 | 769.90556 | anthelmintic, androgen receptor blocker |  |
| QUINACRINE HYDROCHLORIDE | C23H32Cl3N3O | 472.88999 | anthelmintic, antimalarial, intercalating agent |  |
| QUINIDINE GLUCONATE | C26H36N2O9 | 520.58482 | antiarrhythmic, antimalarial |  |
| QUININE | C20H24N2O2 | 324.42648 | antimalarial, skeletal muscle relaxant |  |
| GALLIC ACID | C7H6O5 | 170.12287 | antineoplastic, astringent, antibacterial |  |
| RESERPINE | C33H40N2O9 | 608.69475 | antihypertensive |  |
| RESORCINOL | C6H6O2 | 110.11352 | keratolytic, antiseborheic |  |
| RIFAMPIN | C43H58N4O12 | 822.96131 | antibacterial (tuberculostatic) |  |
| ROXARSONE | C6H6AsNO6 | 263.03942 | antibacterial |  |
| SALICYL ALCOHOL | C7H8O2 | 124.14061 | anesthetic (local), antiinflammatory |  |
| SALICYLAMIDE | C7H7NO2 | 137.13934 | analgesic |  |
| SODIUM SALICYLATE | C7H5NaO3 | 160.1059 | keratolytic |  |
| SCOPOLAMINE HYDROBROMIDE | C17H22BrNO4 | 384.27319 | anticholinergic, treatment of motion sickness |  |
| SALICYLIC ACID | C7H6O3 | 138.12407 | keratolytic |  |
| RIBAVIRIN | C8H12N4O5 | 244.20864 | antiviral |  |
| SPECTINOMYCIN HYDROCHLORIDE | C14H26Cl2N2O7 | 405.27852 | antibacterial |  |
| SPIRONOLACTONE | C24H32O4S | 416.58424 | diuretic, aldosterone antagonist |  |
| STREPTOMYCIN SULFATE [5mM; 10% aq DMSO + 5mM ethanolamine] | C21H41N7O16S | 679.66222 | antibacterial (tuberculostatic) |  |
| STREPTOZOSIN | C8H15N3O7 | 265.22465 | antineoplastic, alkylating agent |  |
| SULFABENZAMIDE | C13H12N2O3S | 276.31619 | antibacterial |  |
| SULFACETAMIDE | C8H10N2O3S | 214.2445 | antibacterial |  |
| SULFADIAZINE | C10H10N4O2S | 250.2808 | antibacterial |  |
| SULFAMERAZINE | C11H12N4O2S | 264.30789 | antibacterial |  |
| SULFAMETHAZINE | C12H14N4O2S | 278.33498 | antibacterial |  |
| SULFAMETHIZOLE | C9H10N4O2S2 | 270.33365 | antibacterial |  |
| SULFAMETHOXAZOLE | C10H11N3O3S | 253.28147 | antibacterial, antipneumocystis |  |
| SULFAPYRIDINE | C11H11N3O2S | 249.29322 | antibacterial, dermatitis herpetiformis therapy |  |
| SULFASALAZINE | C18H14N4O5S | 398.40008 | anticolitis and Crohn's disease |  |
| SULFATHIAZOLE | C9H9N3O2S2 | 255.31898 | antibacterial |  |
| SULFINPYRAZONE | C23H20N2O3S | 404.49145 | uricosuric |  |
| SULFISOXAZOLE | C11H13N3O3S | 267.30856 | antibacterial |  |
| SULINDAC | C20H17FO3S | 356.41909 | antiinflammatory |  |
| TAMOXIFEN CITRATE | C32H37NO8 | 563.65359 | estrogen antagonist, antineoplastic |  |
| TERBUTALINE HEMISULFATE | C12H21NO7S | 323.36767 | betaadrenergic agonist, bronchodilator |  |
| TETRACAINE HYDROCHLORIDE | C15H25ClN2O2 | 300.8317 | anesthetic (local) |  |
| TETRACYCLINE HYDROCHLORIDE | C22H25ClN2O8 | 480.90615 | antibacterial, antiamebic, antirickettsial |  |
| TETRAHYDROZOLINE HYDROCHLORIDE | C13H17ClN2 | 236.74684 | adrenergic agonist, nasal decongestant |  |
| THEOPHYLLINE | C7H8N4O2 | 180.16741 | bronchodilator |  |
| THIABENDAZOLE | C10H7N3S | 201.25139 | anthelmintic |  |
| THIMEROSAL | C9H9HgNaO2S | 404.81468 | antiinfective, preservative |  |
| THIOGUANINE | C5H5N5S | 167.1931 | antineoplastic, purine antimetabolite |  |
| THIORIDAZINE HYDROCHLORIDE | C21H27ClN2S2 | 407.04374 | antipsychotic |  |
| PROPAFENONE HYDROCHLORIDE | C21H28ClNO3 | 377.91521 | antiarrhythmic |  |
| TIMOLOL MALEATE | C17H28N4O7S | 432.49931 | betaadrenergic blocker |  |
| TOBRAMYCIN | C18H37N5O9 | 467.52369 | antibacterial, inhibits protein synthesis |  |
| TOLAZOLINE HYDROCHLORIDE | C10H13ClN2 | 196.68151 | adrenergic blocker |  |
| TOLBUTAMIDE | C12H18N2O3S | 270.35286 | antidiabetic |  |
| TOLMETIN SODIUM | C15H14NNaO3 | 279.27353 | antiinflammatory |  |
| TOLNAFTATE | C19H17NOS | 307.41744 | antifungal |  |
| TRANYLCYPROMINE SULFATE | C9H13NO4S | 231.27226 | antidepressant, MAO inhibitor |  |
| TRIACETIN | C9H14O6 | 218.20833 | antifungal (topical) |  |
| TRIAMCINOLONE | C21H27FO6 | 394.44414 | glucocorticoid |  |
| IODOFORM | CHI3 | 393.73232 | antisptic, disinfectant |  |
| RUTIN | C27H30O16 | 610.53055 | vascular protectant |  |
| TRIAMTERENE | C12H11N7 | 253.26837 | diuretic |  |
| TRICHLORMETHIAZIDE | C8H8Cl3N3O4S2 | 380.65766 | diuretic, antihypertensive |  |
| TRIFLUOPERAZINE HYDROCHLORIDE | C21H26Cl2F3N3S | 480.42667 | antipsychotic |  |
| TRIHEXYPHENIDYL HYDROCHLORIDE | C20H32ClNO | 337.93714 | anticholinergic, antiparkinsonian |  |
| TRIMEPRAZINE TARTRATE | C22H28N2O6S | 448.54226 | antipruritic |  |
| TRIMETHOBENZAMIDE HYDROCHLORIDE | C21H29ClN2O5 | 424.92868 | antiemetic |  |
| TRIMETHOPRIM | C14H18N4O3 | 290.32456 | antibacterial |  |
| TRIOXSALEN | C14H12O3 | 228.24994 | melanizing agent, antipsoriatic |  |
| TRIPELENNAMINE CITRATE | C22H29N3O7 | 447.49233 | antihistaminic |  |
| TRIPROLIDINE HYDROCHLORIDE | C19H23ClN2 | 314.86156 | antihistaminic |  |
| TROPICAMIDE | C17H20N2O2 | 284.36115 | anticholinergic (opthalmic) |  |
| TRYPTOPHAN (L) | C11H12N2O2 | 204.23049 | antidepressant, nutrient; LD50(rat) 1634 mg/kg ip |  |
| TUAMINOHEPTANE SULFATE | C7H19NO4S | 213.29778 | adrenergic agonist |  |
| TUBOCURARINE CHLORIDE PENTAHYDRATE | C37H52Cl2N2O11 | 771.73979 | muscle relaxant (skeletal) |  |
| UREA | CH4N2O | 60.05583 | diuretic, keratolytic |  |
| URSODIOL | C24H40O4 | 392.584 | anticholelithogenic; LD50(rat) 890 mg/kg ip |  |
| VALPROATE SODIUM | C8H15NaO2 | 166.19735 | anticonvulsant |  |
| VANCOMYCIN HYDROCHLORIDE | C66H76Cl3N9O24 | 1485.74652 | antibacterial |  |
| VIDARABINE | C10H13N5O4 | 267.24621 | antiviral |  |
| VINBLASTINE SULFATE | C46H60N4O13S | 909.0741 | antineoplastic, spindle poison |  |
| WARFARIN | C19H16O4 | 308.33697 | anticoagulant, rodenticide |  |
| XYLOMETAZOLINE HYDROCHLORIDE | C16H25ClN2 | 280.84405 | adrenergic agonist, nasal decongestant |  |
| ZOMEPIRAC SODIUM | C15H14ClNO3 | 291.73673 | analgesic, antiinflammatory |  |
| TIOTROPIUM BROMIDE | C19H22BrNO4S2 | 472.42349 | anticholinergic |  |
| PHENACETIN | C10H13NO2 | 179.22061 | analgesic, antipyretic |  |
| CHLOROQUINE DIPHOSPHATE | C18H32ClN3O8P2 | 515.87164 | antimalarial, antiamebic, antirheumatic, intercalating agent |  |
| PHENYLMERCURIC ACETATE | C8H8HgO2 | 336.74176 | antifungal, antimicrobial |  |
| AZELAIC ACID | C9H16O4 | 188.22547 | antiacne, antiproliferative agent |  |
| EFLORNITHINE HYDROCHLORIDE HYDRATE | C6H15ClF2N2O3 | 236.64785 | ornithine decarboxylase inhibitor, antineoplastic, antiprotozoal |  |
| DICUMAROL | C19H12O6 | 336.30389 | anticoagulant |  |
| YOHIMBINE HYDROCHLORIDE | C21H27ClN2O3 | 390.91394 | alpha adrenergic blocker, mydriatic, antidepressant |  |
| ACEBUTOLOL HYDROCHLORIDE | C18H29ClN2O4 | 372.89583 | antihypertensive, antianginal, antiarrhythmic |  |
| ADENOSINE 5-MONOPHOSPHATE | C10H14N5O7P | 347.22618 | vasodilator, neuromodulator |  |
| KETOTIFEN FUMARATE | C23H23NO5S | 425.50746 | antiasthmatic |  |
| BETAHISTINE DIHYDROCHLORIDE | C8H14Cl2N2 | 209.12018 | vasodilator |  |
| TAGATOSE | C6H12O6 | 180.15894 | non-nutritutive sweetener |  |
| MYCOPHENOLIC ACID | C17H20O6 | 320.34535 | immune suppressant, antineoplastic, antiviral |  |
| INDOPROFEN | C17H15NO3 | 281.314 | analgesic, antiinflammatory |  |
| ALBUTEROL | C13H21NO3 | 239.31722 | bronchodilator, tocolytic |  |
| CRYOFLURANE | C2Cl2F4 | 170.9219 | anesthetic (local) |  |
| CAPTOPRIL | C9H15NO3S | 217.2888 | antihypertensive |  |
| CIMETIDINE | C10H16N6S | 252.34322 | antiulcer |  |
| NEBIVOLOL HYDROCHLORIDE | C22H26ClF2NO4 | 441.90662 | antihypertensive, beta-adrenergic blocker, vasodilator |  |
| HYDRASTINE (1R, 9S) | C21H21NO6 | 383.40462 | antihypertensive, sedative, antibacterial |  |
| LIDOCAINE HYDROCHLORIDE | C14H23ClN2O | 270.80521 | anesthetic (local), antiarrhythmic |  |
| CINCHONINE | C19H22N2O | 294.39999 | antimalarial |  |
| NALIDIXIC ACID | C12H12N2O3 | 232.24104 | antibacterial |  |
| BUTAMBEN | C11H15NO2 | 193.2477 | anesthetic (local); inhibitor of DRG IA/Kv4.2 current |  |
| CEFACLOR | C15H14ClN3O4S | 367.81353 | antibacterial |  |
| IODIPAMIDE | C20H14I6N2O6 | 1139.77078 | radioopaque agent |  |
| LEVOTHYROXINE SODIUM | C15H10I4NNaO4 | 798.85865 | antihypercholesterimic, thyromimetic |  |
| LIOTHYRONINE | C15H12I3NO4 | 650.98039 | thyroid hormone blocker, antidepressant |  |
| ALLANTOIN | C4H6N4O3 | 158.11742 | wound healing agent |  |
| ALTHIAZIDE | C11H14ClN3O4S3 | 383.89693 | diuretic |  |
| ADENINE | C5H5N5 | 135.1291 | Vitamin B4 |  |
| AMINACRINE | C13H10N2 | 194.23805 | local antiseptic |  |
| BEKANAMYCIN SULFATE | C18H39N5O14S | 581.60063 | antibacterial |  |
| BUDESONIDE | C25H34O6 | 430.54613 | antiinflammatory |  |
| CANRENOIC ACID, POTASSIUM SALT | C22H29KO4 | 396.57603 | aldosterone antagonist, diuretic |  |
| CHENODIOL | C24H40O4 | 392.584 | anticholithogenic, antilipemic agent |  |
| CHOLECALCIFEROL | C27H44O | 384.65113 | vitamin D3 |  |
| CYANOCOBALAMIN | C63H88CoN14O14P | 1355.39621 | vitamin, coenzyme B12 |  |
| CHOLESTEROL | C27H46O | 386.66707 | emulsifying agent |  |
| PIPERINE | C17H19NO3 | 285.34588 | analeptic, antibacterial |  |
| ETOPOSIDE | C29H32O13 | 588.57059 | antineoplastic |  |
| DEHYDROCHOLIC ACID | C24H34O5 | 402.53558 | choleretic |  |
| AZATADINE MALEATE | C24H26N2O4 | 406.48582 | H1-antihistamine |  |
| FLUMEQUINE | C14H12FNO3 | 261.25504 | antibacterial |  |
| FLUNARIZINE HYDROCHLORIDE | C26H28Cl2F2N2 | 477.42926 | vasodilator |  |
| FLUPHENAZINE HYDROCHLORIDE | C22H28Cl2F3N3OS | 510.45316 | antipsychotic, H1 antihistamine |  |
| FLUTAMIDE | C11H11F3N2O3 | 276.21712 | antiandrogen, antineoplastic, Nuclear Hormone receptor antagonist |  |
| MONTELUKAST SODIUM | C35H35ClNNaO3S | 608.1809 | leucotriene antagonist, antiasthmatic |  |
| FAMOTIDINE | C8H15N7O2S3 | 337.44645 | H2 antihistamine |  |
| ETODOLAC | C17H21NO3 | 287.36182 | antiinflammatory |  |
| FENOTEROL HYDROBROMIDE | C17H22BrNO4 | 384.27319 | betaadrenergic agonist |  |
| FENBUFEN | C16H14O3 | 254.28818 | antiinflammatory |  |
| FENOFIBRATE | C20H21ClO4 | 360.84097 | antihyperlipidemic |  |
| FENOPROFEN | C15H14O3 | 242.27703 | antiinflammatory |  |
| FLUFENAMIC ACID | C14H10F3NO2 | 281.2365 | antiinflammatory, analgesic |  |
| FENBENDAZOLE | C15H13N3O2S | 299.35376 | anthelmintic |  |
| FENSPIRIDE HYDROCHLORIDE | C15H21ClN2O2 | 296.79982 | antiinflammatory, bronchodilator |  |
| MEFENAMIC ACID | C15H15NO2 | 241.2923 | antiinflammatory, analgesic |  |
| METHACYCLINE HYDROCHLORIDE | C22H23ClN2O8 | 478.89021 | antibacterial |  |
| PUROMYCIN DIHYDROCHLORIDE | C22H31Cl2N7O5 | 544.44227 | antineoplastic, antiprotozoal |  |
| MEFEXAMIDE HYDROCHLORIDE | C15H25ClN2O3 | 316.8311 | CNS stimulant |  |
| PROBUCOL | C31H48O2S2 | 516.85501 | antihyperlipidemic |  |
| MEBENDAZOLE | C16H13N3O3 | 295.30031 | anthelmintic |  |
| PROPOXYCAINE HYDROCHLORIDE | C16H27ClN2O3 | 330.85819 | local anesthetic |  |
| MEBEVERINE HYDROCHLORIDE | C25H36ClNO5 | 466.02237 | muscle relaxant (smooth) |  |
| MECLOCYCLINE SULFOSALICYLATE | C29H27ClN2O14S | 695.06054 | antibacterial |  |
| PROGLUMIDE | C18H26N2O4 | 334.41892 | anticholinergic |  |
| MINAPRINE HYDROCHLORIDE | C17H24Cl2N4O | 371.31303 | antidepressant, psychotropic |  |
| MEMANTINE HYDROCHLORIDE | C12H22ClN | 215.76884 | muscle relaxant (skeletal) |  |
| CINCHONIDINE | C19H22N2O | 294.39999 | antimalarial |  |
| TRAMIPROSATE | C3H9NO3S | 139.17408 | antibacterial; GABA agonist |  |
| ATENOLOL | C14H22N2O3 | 266.34304 | beta adrenergic blocker |  |
| CAPSAICIN | C18H27NO3 | 305.42079 | analgesic (topical), depletes Substance P, neurotoxic |  |
| FLUTICASONE FUROATE | C27H29F3O6S | 538.58778 | antiasthma, COPD therapy |  |
| ROSIGLITAZONE MALEATE | C22H23N3O7S | 473.50851 | antidiabetic |  |
| NICERGOLINE | C24H26BrN3O3 | 484.39712 | vasodilator |  |
| PIMOZIDE | C28H29F2N3O | 461.55963 | antipsychotic |  |
| NICARDIPINE HYDROCHLORIDE | C26H30ClN3O6 | 515.9985 | vasodilator |  |
| NEFOPAM | C17H19NO | 253.34708 | analgesic |  |
| PIRENZEPINE HYDROCHLORIDE | C19H23Cl2N5O2 | 424.33346 | antiulcer |  |
| PRAMOXINE HYDROCHLORIDE | C17H28ClNO3 | 329.87061 | anesthetic (topical) |  |
| QUINOXYFEN | C15H8Cl2FNO | 308.14151 | fungicide |  |
| SULFACHLORPYRIDAZINE | C10H9ClN4O2S | 284.72583 | antibacterial |  |
| SULFADIMETHOXINE | C12H14N4O4S | 310.33378 | antibacterial |  |
| SULFAQUINOXALINE SODIUM | C14H11N4NaO2S | 322.32317 | antibacterial, coccidiostat |  |
| SULFAMONOMETHOXINE | C11H12N4O3S | 280.30729 | antibacterial |  |
| SULCONAZOLE NITRATE | C18H16Cl3N3O3S | 460.76952 | antifungal |  |
| RITODRINE HYDROCHLORIDE | C17H22ClNO3 | 323.82279 | muscle relaxant (smooth) |  |
| SULPIRIDE (S [-]) | C15H23N3O4S | 341.43226 | dopamine receptor antagonist, antipsychotic |  |
| RANITIDINE HYDROCHLORIDE | C13H23ClN4O3S | 350.87026 | H2 antihistamine |  |
| SPIPERONE | C23H26FN3O2 | 395.48097 | antipsychotic |  |
| SULOCTIDIL | C20H35NOS | 337.57205 | peripheral vasodilator |  |
| RONIDAZOLE | C6H8N4O4 | 200.15506 | antiprotozoal |  |
| SULFAMETER | C11H12N4O3S | 280.30729 | antibacterial |  |
| SULFAMETHOXYPYRIDAZINE | C11H12N4O3S | 280.30729 | antibacterial |  |
| SACCHARIN | C7H5NO3S | 183.1868 | sweetener |  |
| OCTODRINE | C8H19N | 129.24733 | vasoconstrictor, anesthetic (local) |  |
| ERYTHROMYCIN ESTOLATE | C52H97NO18S | 1056.41279 | antibacterial |  |
| ESTRADIOL BENZOATE | C25H28O3 | 376.50011 | estrogen |  |
| ECONAZOLE NITRATE | C18H16Cl3N3O4 | 444.70492 | antifungal |  |
| FLUNISOLIDE | C24H31FO6 | 434.50947 | antiinflammatory |  |
| FLUMETHASONE | C22H28F2O5 | 410.46226 | antiinflammatory |  |
| XYLAZINE | C12H16N2S | 220.33872 | analgesic |  |
| TOLAZAMIDE | C14H21N3O3S | 311.40577 | antidiabetic |  |
| GALANTAMINE | C17H21NO3 | 287.36182 | anticholinesterase, analgesic, antiAlzheimer |  |
| RETINOL | C20H30O | 286.4615 | vitamin A |  |
| LANATOSIDE C | C49H76O20 | 985.14007 | cardiotonic |  |
| ENALAPRIL MALEATE | C24H32N2O9 | 492.53064 | ACE inhibitor, antihypertensive |  |
| KETOPROFEN | C16H14O3 | 254.28818 | antiinflammatory |  |
| LISINOPRIL | C21H31N3O5 | 405.49832 | ACE inhibitor |  |
| BUMETANIDE | C17H20N2O5S | 364.42335 | diuretic |  |
| CARBENOXOLONE SODIUM | C34H48Na2O7 | 614.73706 | antiinflammatory, antisecretory, antiulcer |  |
| ESOMEPRAZOLE POTASSIUM | C17H19MgN3O3S | 369.73528 | gastric acid secretion inhibitor |  |
| AVANAFIL | C23H26ClN7O3 | 483.96177 | erectile dysfunction |  |
| ISOTRETINON | C20H28O2 | 300.44496 | antiacne, antineoplastic |  |
| MESNA | C2H5NaO3S2 | 164.17815 | mucolytic |  |
| TRETINOIN | C20H28O2 | 300.44496 | keratolytic, antiacne, antineoplastic |  |
| OUABAIN OCTAHYDRATE | C29H60O20 | 728.78955 | antiarrhythmic, cardiotonic, hypertensive, Na/K ATPase inhibitor |  |
| FOSCARNET SODIUM | CNa3O5P | 191.95135 | antiviral |  |
| LENALIDOMIDE | C13H13N3O3 | 259.26686 | immunomodulator |  |
| PHTHALYLSULFATHIAZOLE | C17H13N3O5S2 | 403.43826 | antibacterial |  |
| SUCCINYLSULFATHIAZOLE | C13H13N3O5S2 | 355.39366 | antibacterial |  |
| CEPHALEXIN | C16H17N3O4S | 347.39559 | antibacterial |  |
| ACARBOSE | C25H43NO18 | 645.61736 | alpha-glucosidase & saccharase inhibitor, antidiabetes, antihyperlipidaemia, antiobesity |  |
| CEFOXITIN SODIUM | C16H16N3NaO7S2 | 449.43962 | antibacterial |  |
| SURAMIN HEXASODIUM | C51H34N6Na6O23S6 | 1429.18883 | antiprotozoal, trypanocidal, antiviral |  |
| CEFUROXIME SODIUM | C16H15N4NaO8S | 446.37375 | antibacterial |  |
| RALOXIFENE HYDROCHLORIDE | C28H28ClNO4S | 510.05666 | antiestrogen |  |
| CEFAMANDOLE SODIUM | C18H17N6NaO5S2 | 484.49119 | antibacterial |  |
| FOSFOMYCIN CALCIUM | C3H5CaO4P | 176.1247 | antibacterial |  |
| CEFMETAZOLE SODIUM | C15H16N7NaO5S3 | 493.52047 | antibacterial |  |
| CEFAMANDOLE NAFATE | C19H17N6NaO6S2 | 512.50174 | antibacterial |  |
| CEFOPERAZONE | C25H27N9O8S2 | 645.67744 | antibacterial |  |
| BEZAFIBRATE | C19H20ClNO4 | 361.82855 | antihyperlipidemic |  |
| DAPAGLIFLOZIN | C21H25ClO6 | 408.8828 | antidiabetic, SGLT2 inhibitor |  |
| ALRESTATIN | C14H9NO4 | 255.23213 | aldose reductase inhibitor |  |
| PROADIFEN HYDROCHLORIDE | C23H32ClNO2 | 389.96999 | cytochrome P450 inhibitor, Ca antagonist, anesthetic (local) |  |
| AMYLENE HYDRATE | C5H12O | 88.15079 | hypnotic, antidiabetic |  |
| CISPLATIN | H6Cl2N2Pt | 300.05722 | antineoplastic, convulsant |  |
| ZIDOVUDINE [AZT] | C10H13N5O4 | 267.24621 | RT transferase inhibitor, antiviral |  |
| AZACITIDINE | C8H12N4O5 | 244.20864 | antineoplastic, pyrimidine antimetabolite |  |
| CYCLOHEXIMIDE | C15H23NO4 | 281.35486 | antipsoriatic, protein synthesis inhibitor |  |
| CEFOTETAN | C17H17N7O8S4 | 575.62314 | antibacterial |  |
| TINIDAZOLE | C8H13N3O4S | 247.27451 | antiprotozoal |  |
| CARBIDOPA | C10H14N2O4 | 226.23408 | decarboxylase inhibitor, antiparkinsonism |  |
| PIRACETAM | C6H10N2O2 | 142.1588 | antinauseant |  |
| ETHOSUXIMIDE | C7H11NO2 | 141.17122 | anticonvulsant |  |
| PIPERIDOLATE HYDROCHLORIDE | C21H26ClNO2 | 359.89987 | antispasmodic |  |
| AMPYZINE SULFATE | C6H11N3O4S | 221.23627 | CNS stimulant |  |
| CYCLOSPORINE | C62H111N11O12 | 1202.64247 | immunosuppressant |  |
| FLEROXACIN | C17H18F3N3O3 | 369.34651 | antibacterial |  |
| ASCORBIC ACID | C6H8O6 | 176.12706 | antiscorbutic, antiviral |  |
| MENADIONE | C11H8O2 | 172.18521 | prothrombogenic agent |  |
| SALICIN | C13H18O7 | 286.28421 | analgesic, antipyretic |  |
| MONENSIN SODIUM | C36H61NaO11 | 692.87077 | antibacterial |  |
| ISOCONAZOLE NITRATE | C18H15Cl4N3O4 | 479.14995 | antibacterial, antifungal |  |
| LEVETIRACETAM | C8H14N2O2 | 170.21298 | antiepileptic |  |
| alpha-TOCHOPHEROL [4 mM] | C29H50O2 | 430.72065 | vitamin E, antioxidant |  |
| CAPECITABINE | C15H22FN3O6 | 359.35749 | antineoplastic |  |
| alpha-TOCHOPHERYL ACETATE [4mM] | C31H52O3 | 472.75829 | vitamin E |  |
| SISOMICIN SULFATE | C19H39N5O11S | 545.61358 | antibacterial, binds to ribosomes |  |
| FLUBENDAZOLE | C16H12FN3O3 | 313.29074 | anthelmitic |  |
| THIAMINE HYDROCHLORIDE | C12H18Cl2N4OS | 337.27346 | vitamin B1, enzyme cofactor |  |
| NALOXONE HYDROCHLORIDE | C19H22ClNO4 | 363.84449 | narcotic antagonist |  |
| DIAVERIDINE | C13H16N4O2 | 260.29807 | antibacterial |  |
| LOMUSTINE | C9H16ClN3O2 | 233.69977 | antineoplastic |  |
| LORNOXICAM | C13H10ClN3O4S2 | 371.82335 | analgesic, antiinflammatory |  |
| IOHEXOL | C17H22I3N3O8 | 777.09339 | radiopaque diagnostic aid |  |
| MEROPENEM | C17H25N3O5S | 383.4699 | antibacterial |  |
| NEFAZODONE HYDROCHLORIDE | C25H32ClN5O2 | 470.01909 | antidepressant |  |
| NIFURSOL | C12H7N5O9 | 365.21769 | antiprotozoal |  |
| MILRINONE | C12H9N3O | 211.22503 | cardiotonic |  |
| DROPERIDOL | C22H22FN3O2 | 379.43794 | neuroleptic |  |
| GATIFLOXACIN | C19H22FN3O4 | 375.40329 | antibacterial |  |
| PYRETHRINS | C21H28O3 | 328.45551 | insecticide, Na channel toxin |  |
| OXYPHENCYCLIMINE HYDROCHLORIDE | C20H29ClN2O3 | 380.91873 | anticholinergic |  |
| RACTOPAMINE HYDROCHLORIDE | C18H24ClNO3 | 337.84988 | beta-adrenergic agonist, growth stimulant |  |
| MORANTEL CITRATE | C18H24N2O7S | 412.46518 | anthelmintic |  |
| ROXATIDINE ACETATE HYDROCHLORIDE | C19H29ClN2O4 | 384.90698 | antiulcer |  |
| SERATRODAST | C22H26O4 | 354.45012 | Thx-antagonist, antiasthmatic, antiinflammatory |  |
| SPARFLOXACIN | C19H22F2N4O3 | 392.40899 | antibacterial |  |
| STAVUDINE | C10H12N2O4 | 224.21814 | antiviral |  |
| SULBACTAM | C8H11NO5S | 233.24457 | b-lactamase inhibitor |  |
| AZAPERONE | C19H22FN3O | 327.40509 | tranquilizer, neuroleptic, alpha adrenergic blocker |  |
| LOMEFLOXACIN HYDROCHLORIDE | C17H20ClF2N3O3 | 387.81705 | antibacterial |  |
| URACIL | C4H4N2O2 | 112.08868 | antineoplastic |  |
| ETHISTERONE | C21H28O2 | 312.45611 | progestogen |  |
| DICLAZURIL | C17H9Cl3N4O2 | 407.64588 | coccidiostat |  |
| IDOXURIDINE | C9H11IN2O5 | 354.10282 | antiviral |  |
| ABACAVIR SULFATE | C14H20N6O5S | 384.4167 | antiviral |  |
| LEVONORGESTREL | C21H28O2 | 312.45611 | progestin |  |
| LEVOSIMENDAN | C14H12N6O | 280.29134 | vasodilator |  |
| LOFEXIDINE HYDROCHLORIDE | C11H13Cl3N2O | 295.59806 | antihypertensive |  |
| DOXOFYLLINE | C11H14N4O4 | 266.25863 | bronchdilator |  |
| DIFLOXACIN HYDROCHLORIDE | C21H20ClF2N3O3 | 435.86165 | antibacterial, DNA gyrase inhibitor |  |
| ZALCITABINE | C9H13N3O3 | 211.22226 | antiviral |  |
| RITONAVIR | C37H48N6O5S2 | 720.96031 | antiviral |  |
| GANCICLOVIR HYDRATE | C9H15N5O5 | 273.2504 | antiviral |  |
| DEXIBUPROFEN | C13H18O2 | 206.28721 | analgesic, antiinflammatory |  |
| PIZOTYLINE MALATE | C23H27NO5S | 429.53934 | 5HT antagonist, antimigraine |  |
| MEPHENESIN | C10H14O3 | 182.22128 | muscle relaxant (skeletal) |  |
| NEPAFENAC | C15H14N2O2 | 254.29103 | antiinflammatory, analgesic |  |
| CANDESARTAN | C24H20N6O3 | 440.4654 | antihypertensive, angiotensin II inhibitor |  |
| ADAPALENE | C28H28O3 | 412.53356 | anti-acne |  |
| AMLEXANOX | C16H14N2O4 | 298.30098 | anti-allergic |  |
| AMPROLIUM | C14H19ClN4 | 278.78733 | coccidiostat |  |
| RONNEL | C8H8Cl3O3PS | 321.54796 | insecticide |  |
| HYDROXYZINE HYDROCHLORIDE | C21H29Cl3N2O2 | 447.83648 | anxiolytic, antihistaminic |  |
| EPRINOMECTIN | C50H75NO14 | 914.15355 | antiparasitic |  |
| FENCLONINE (+/-) | C9H11Cl2NO2 | 236.09952 | serotonin inhibitor; tryptophan hydroxylase inhibitor |  |
| LINEZOLID | C16H20FN3O4 | 337.3539 | antibacterial |  |
| LUMIRACOXIB | C15H13ClFNO2 | 293.72776 | antiinflammatory, analgesic, antiarthritic |  |
| DIPERODON HYDROCHLORIDE | C22H28ClN3O4 | 433.93916 | analgesic, anesthetic |  |
| OLSALAZINE SODIUM | C14H8N2Na2O6 | 346.20926 | antiinflammatory |  |
| LEVOMENTHOL | C10H20O | 156.2703 | analgesic (topical), antipruritic agent |  |
| TREHALOSE DIHYDRATE | C12H26O13 | 378.33322 | sweetener, stabilizer |  |
| DIHYDROSTREPTOMYCIN SESQUISULFATE [5mM/10% aq DMSO] | C21H43N7O16S | 681.67816 | antibacterial, tuberculostatic |  |
| ANTAZOLINE | C17H19N3 | 265.36108 | antihistaminic |  |
| OFLOXACIN | C18H20FN3O4 | 361.3762 | antibacterial |  |
| PERPHENAZINE | C21H26ClN3OS | 403.97787 | antipsychotic |  |
| AMINOPHYLLINE | C9H16N6O2 | 240.26687 | bronchodilator, smooth muscle relaxant |  |
| BALSALAZIDE DISODIUM | C17H13N3Na2O6 | 401.28926 | antiinflammatory |  |
| LOPINAVIR | C37H48N4O5 | 628.81891 | antiviral; HIV protease inhibitor |  |
| CEFEPIME HYDROCHLORIDE | C19H25ClN6O5S2 | 517.0293 | antibacterial |  |
| AZASERINE | C5H7N3O4 | 173.12924 | antineoplastic, amino acid antagonist |  |
| CEFORANIDE | C20H21N7O6S2 | 519.56167 | antibacterial |  |
| PROPARACAINE HYDROCHLORIDE | C16H27ClN2O3 | 330.85819 | local anesthetic |  |
| PREDNICARBATE | C27H36O8 | 488.58317 | antiinflammatory, glucocorticoid |  |
| ONDANSETRON HYDROCHLORIDE | C18H20ClN3O | 329.8326 | antiallergic, antiemetic, anti-schizophrenic |  |
| CINROMIDE | C11H12BrNO | 254.12839 | anticonvulsant |  |
| CINTRIAMIDE | C12H15NO4 | 237.25765 | antipsychotic |  |
| BENZOIC ACID | C7H6O2 | 122.12467 | antifungal |  |
| BENZYL BENZOATE | C14H12O2 | 212.25054 | scabicide |  |
| BENZOYL PEROXIDE | C14H10O4 | 242.2334 | keratolytic |  |
| BETAINE HYDROCHLORIDE | C5H12ClNO2 | 153.60989 | antiarteriosclerotic, hypolipaemic, hepatoprotectant |  |
| BIOTIN | C10H16N2O3S | 244.31462 | vitamin B complex |  |
| AKLOMIDE | C7H5ClN2O3 | 200.5825 | antiprotozoal, coccidiostat |  |
| MONOBENZONE | C13H12O2 | 200.23939 | depigmentor |  |
| NICOTINYL ALCOHOL TARTRATE | C10H13NO7 | 259.21761 | vasodilator |  |
| FLOXURIDINE | C9H11FN2O5 | 246.19682 | antineoplastic, antimetabolite |  |
| ALTRETAMINE | C9H18N6 | 210.28401 | antineoplastic |  |
| AMINOHIPPURIC ACID | C9H10N2O3 | 194.19165 | renal function diagnosis |  |
| MEFLOQUINE HYDROCHLORIDE | C17H17ClF6N2O | 414.78124 | antimalarial |  |
| ADIPHENINE HYDROCHLORIDE | C20H26ClNO2 | 347.88872 | muscle relaxant (smooth) |  |
| GADODIAMIDE | C16H26GdN5O8 | 573.66432 | diagnostic aid |  |
| METHYSERGIDE MALEATE | C25H31N3O6 | 469.54232 | vasoconstrictor, antimigraine |  |
| AMIFOSTINE | C5H15N2O3PS | 214.2247 | radioprotectant |  |
| INAMRINONE | C10H9N3O | 187.20273 | cardiac stimulant |  |
| TIAPRIDE HYDROCHLORIDE | C15H25ClN2O4S | 364.8945 | neuroleptic |  |
| GLUCONOLACTONE | C6H10O6 | 178.143 | chelating agent |  |
| AZLOCILLIN SODIUM | C20H22N5NaO6S | 483.48204 | antibacterial |  |
| ERYTHRITOL | C4H10O4 | 122.1219 | sweetener |  |
| BENDROFLUMETHIAZIDE | C15H14F3N3O4S2 | 421.41973 | diuretic, antihypertensive |  |
| OLMESARTAN | C24H26N6O3 | 446.51322 | Angiotensin II inhibitor, antihypertensive |  |
| BROMHEXINE HYDROCHLORIDE | C14H21Br2ClN2 | 412.59787 | expectorant |  |
| TYROSINE HYDROCHLORIDE | C9H12ClNO3 | 217.65389 | amino acid, nutrient |  |
| CEFTRIAXONE SODIUM TRIHYDRATE | C18H22N8Na2O10S3 | 652.59524 | antibacterial |  |
| VINPOCETINE | C22H26N2O2 | 350.46472 | cerebral vasodilator, antimotion |  |
| TRIMIPRAMINE MALEATE | C24H30N2O4 | 410.5177 | antidepressant |  |
| RITANSERIN | C27H25F2N3OS | 477.5806 | antiserotonin |  |
| TRAZODONE HYDROCHLORIDE | C19H23Cl2N5O | 408.33406 | antidepressant |  |
| THONZYLAMINE HYDROCHLORIDE | C16H23ClN4O | 322.84091 | antihistamine |  |
| THIAMPHENICOL | C12H15Cl2NO5S | 356.22705 | antibacterial |  |
| TENOXICAM | C13H11N3O4S2 | 337.37832 | antiinflammatory |  |
| CHLOROXINE | C9H5Cl2NO | 214.0523 | chelating agent, antiseborrheic |  |
| CHLORPROTHIXENE HYDROCHLORIDE | C18H19Cl2NS | 352.32883 | antipsychotic |  |
| CINNARAZINE | C26H28N2 | 368.52646 | H1 antihistamine |  |
| CYCLOBENZAPRINE HYDROCHLORIDE | C20H22ClN | 311.85804 | muscle relaxant (skeletal) |  |
| DANTROLENE SODIUM | C14H9N4NaO5 | 336.24143 | muscle relaxant (skeletal) |  |
| BETAMETHASONE 17,21-DIPROPIONATE | C28H37FO7 | 504.60129 | glucocorticoid, antiinflammatory |  |
| IMIQUIMOD HYDROCHLORIDE | C14H17ClN4 | 276.77139 | immunomodulator |  |
| EDOXUDINE | C11H16N2O5 | 256.26057 | antiviral |  |
| ENOXACIN | C15H17FN4O3 | 320.32614 | antibacterial |  |
| GRANISETRON HYDROCHLORIDE | C18H25ClN4O | 348.87915 | antiemetic |  |
| PERHEXILINE MALEATE | C23H39NO4 | 393.57158 | coronary vasodilator |  |
| METHAPYRILENE HYDROCHLORIDE | C14H20ClN3S | 297.8526 | H1 antihistamine |  |
| MEPRYLCAINE HYDROCHLORIDE | C14H22ClNO2 | 271.78994 | local anesthetic, 5HT2 agonist |  |
| HALCINONIDE | C24H32ClFO5 | 454.97104 | glucocorticoid, antiinflammatory |  |
| LAPATINIB | C29H26ClFN4O4S | 581.07037 | antineoplastc |  |
| DOXAPRAM HYDROCHLORIDE | C24H31ClN2O2 | 414.97987 | respiratory stimulant |  |
| ISOXICAM | C14H13N3O5S | 335.34081 | antiinflammatory |  |
| LABETALOL HYDROCHLORIDE | C19H25ClN2O3 | 364.8757 | adrenergic blocker |  |
| FLUOROMETHOLONE | C22H29FO4 | 376.47243 | glucocorticoid, antiinflammatory |  |
| LEVAMISOLE HYDROCHLORIDE | C11H13ClN2S | 240.75666 | immunomodulator |  |
| METARAMINOL BITARTRATE | C13H19NO8 | 317.29828 | antihypotensive |  |
| METHAZOLAMIDE | C5H8N4O3S2 | 236.27251 | carbonic anhydrase inhibitor |  |
| METHYLBENZETHONIUM CHLORIDE | C28H44ClNO2 | 462.12138 | antiinfective |  |
| METHYLPREDNISOLONE SODIUM SUCCINATE | C26H33NaO8 | 496.53791 | glucocorticoid, antiinflammatory |  |
| AMSACRINE | C21H19N3O3S | 393.46788 | antineoplastic, immune suppressive |  |
| MIDODRINE HYDROCHLORIDE | C12H19ClN2O4 | 290.74923 | antihypertensive, vasoconstrictor |  |
| CETALKONIUM CHLORIDE | C25H46ClN | 396.10507 | antinfective |  |
| NALTREXONE HYDROCHLORIDE | C20H24ClNO4 | 377.87158 | morphine antagonist |  |
| CYCLOTHIAZIDE | C14H16ClN3O4S2 | 389.88232 | diuretic |  |
| NICLOSAMIDE | C13H8Cl2N2O4 | 327.12571 | anthelmintic, teniacide |  |
| PAROMOMYCIN SULFATE | C23H47N5O18S | 713.71774 | antibacterial, antiamebic |  |
| TRANILAST | C18H17NO5 | 327.33989 | antiallergic, mast cell degranulation inhibitor, angiogenesis blocker |  |
| PRILOCAINE HYDROCHLORIDE | C13H21ClN2O | 256.77812 | anesthetic (local) |  |
| HYDROCORTISONE BUTYRATE | C25H36O6 | 432.56207 | glucocorticoid, antiinflammatory |  |
| ROXITHROMYCIN | C41H76N2O15 | 837.06727 | antibacterial |  |
| MITOXANTRONE HYDROCHLORIDE | C22H30Cl2N4O6 | 517.4136 | antineoplastic |  |
| OXETHAZAINE | C28H41N3O3 | 467.65727 | anesthetic (local) |  |
| DIPYRONE | C13H16N3NaO4S | 333.34397 | analgesic, antipyretic |  |
| SULFANILATE ZINC | C12H12N2O6S2Zn | 409.73724 | antibacterial |  |
| BEXAROTENE | C23H26O3 | 350.46187 | antineoplastic, antidiabetic |  |
| THIRAM | C6H12N2S4 | 240.43194 | antifungal |  |
| THIOTEPA | C6H12N3PS | 189.22044 | antineoplastic, alkylating agent |  |
| TETROQUINONE | C6H4O6 | 172.09518 | keratolytic |  |
| SULFANITRAN | C14H13N3O5S | 335.34081 | antibacterial, coccidiostat |  |
| OXIBENDAZOLE | C12H15N3O3 | 249.27165 | anthelmintic |  |
| PIPOBROMAN | C10H16Br2N2O2 | 356.05922 | antineoplastic, alkylating agent |  |
| NAFRONYL OXALATE | C26H35NO7 | 473.57135 | vasodilator |  |
| QUIPAZINE MALEATE | C17H19N3O4 | 329.35868 | antidepressant, oxytocic |  |
| FOLIC ACID | C19H19N7O6 | 441.40658 | hematopoietic vitamin |  |
| HOMOSALATE | C16H22O3 | 262.35194 | UV screen, analgesic |  |
| SPIRAMYCIN | C43H74N2O14 | 843.07423 | antibacterial |  |
| ESZOPICLONE | C17H17ClN6O3 | 388.81644 | hypnotic, sedative |  |
| CHOLINE CHLORIDE | C5H14ClNO | 139.62643 | choleretic, lipotropic, hepatoprotectant |  |
| CLOFIBRATE | C12H15ClO3 | 242.70455 | antihyperlipidemic |  |
| RESORCINOL MONOACETATE | C8H8O3 | 152.15116 | antiseborrheic, antipruritic |  |
| NIMODIPINE | C21H26N2O7 | 418.45057 | vasodilator |  |
| ACYCLOVIR | C8H11N5O3 | 225.20857 | antiviral |  |
| PENFLURIDOL | C28H27ClF5NO | 523.97849 | antipsychotic |  |
| THALIDOMIDE | C13H10N2O4 | 258.23565 | hypnotic |  |
| IOXILAN | C18H24I3N3O8 | 791.12048 | radiopaque agent |  |
| BENZALKONIUM CHLORIDE HYDRATE | C22H42ClNO | 372.03914 | antiinfective (topical) |  |
| PENTOXIFYLLINE | C13H18N4O3 | 278.31341 | PDE inhibitor, bronchodilator, vasodilator |  |
| CIPROFLOXACIN | C17H18FN3O3 | 331.34971 | antibacterial, fungicide |  |
| BACLOFEN HYDROCHLORIDE (+/-) | C10H13Cl2NO2 | 250.12661 | muscle relaxant (skeletal) |  |
| NABUMETONE | C15H16O2 | 228.29357 | antiinflammatory |  |
| CELECOXIB | C17H14F3N3O2S | 381.37923 | antiarthritic, cyclooxygenase2 inhibitor |  |
| ALENDRONATE SODIUM TRIHYDRATE | C4H19NNaO10P2 | 326.13413 | bone resorption inhibitor, farnesyldiphosphate synthetase inhibitor, antimetastatic |  |
| BLEOMYCIN (bleomycin B2 shown) | C58H94N20O26S4 | 1615.77028 | antineoplastic |  |
| ANETHOLE | C10H12O | 148.20654 | expectorant, gastric stimulant, insecticide |  |
| TERFENADINE | C32H41NO2 | 471.68907 | H1 antihistamine, nonsedating |  |
| MEPHENYTOIN | C12H14N2O2 | 218.25758 | anticonvulsant |  |
| CLOPIDOGREL SULFATE | C16H18ClNO6S2 | 419.90596 | platelet aggregation inhibitor |  |
| LORATADINE | C22H23ClN2O2 | 382.89381 | H1 antihistamine |  |
| SELAMECTIN | C43H63NO11 | 769.98166 | anthelmintic, antiparasitic, antimite |  |
| ATORVASTATIN CALCIUM | C33H34CaFN2O5 | 597.72773 | antihyperlipidemic, HMGCoA reductase inhibitor |  |
| SODIUM GLUCONATE | C6H11NaO7 | 218.14017 | electrolyte replenisher |  |
| CLOBETASOL PROPIONATE | C25H32ClFO5 | 466.98219 | glucocorticoid, antiinflammatory |  |
| CEFTAZIDIME | C22H22N6O7S2 | 546.58464 | antibacterial |  |
| COLFORSIN | C22H34O7 | 410.51208 | adenylate cyclase activator, antiglaucoma, hypotensive, vasodilator |  |
| ISOSORBIDE MONONITRATE | C6H9NO6 | 191.14173 | antianginal |  |
| AMCINONIDE | C28H35FO7 | 502.58535 | glucocorticoid, antiinflammatory |  |
| BUPIVACAINE HYDROCHLORIDE | C18H29ClN2O | 324.89763 | anesthetic (local) |  |
| DROSPIRENONE | C24H30O3 | 366.5049 | aldosterone antagonist |  |
| BEXAROTENE | C24H28O2 | 348.48956 | antineoplastic |  |
| QUININE HDROCHLORIDE | C20H25ClN2O2 | 360.88745 | antimalarial, skeletal muscle relaxant |  |
| GADOTERIDOL | C17H29GdN4O7 | 558.69328 | paramagnetic agent |  |
| SEVOFLURANE | C4H3F7O | 200.05671 | anesthetic |  |
| PHYSOSTIGMINE SALICYLATE | C22H27N3O5 | 413.47759 | cholinergic, anticholinesterase, miotic |  |
| PHENFORMIN HYDROCHLORIDE | C10H16ClN5 | 241.72552 | antidiabetic |  |
| TRIFLUPROMAZINE HYDROCHLORIDE | C18H20ClF3N2S | 388.8857 | antipsychotic |  |
| LAMIVUDINE | C8H11N3O3S | 229.25917 | antiviral |  |
| BETAZOLE HYDROCHLORIDE | C5H11Cl2N3 | 184.06952 | gastric secretion stimulant |  |
| BUTACAINE SULFATE | C18H32N2O6S | 404.52954 | anesthetic (local) |  |
| NITAZOXANIDE | C12H9N3O5S | 307.28663 | antiparasitic |  |
| COLESEVALAM HYDROCHLORIDE (high mol wt copolymer @ 5mg/ml) | C31H72ClN4O | 552.39869 | antihyperlipidemic, cholesterol sequestrant |  |
| BACAMPICILLIN HYDROCHLORIDE | C21H28ClN3O7S | 501.99021 | antibacterial |  |
| VORINOSTAT | C14H20N2O3 | 264.3271 | antineoplastic, histone deacetylase inhibitor |  |
| PACLITAXEL | C47H51NO14 | 853.92882 | antineoplastic |  |
| ACETOPHENAZINE MALEATE | C27H33N3O6S | 527.64456 | antipsychotic |  |
| TOPOTECAN HYDROCHLORIDE | C23H24ClN3O5 | 457.91783 | antineoplastic; topoisomerase I inhibitor |  |
| PHYTONADIONE [5mM] | C31H46O2 | 450.71107 | vitamin K1 |  |
| PREGABALIN | C8H17NO2 | 159.23019 | anticonvulsant |  |
| CARBARSONE | C7H9AsN2O4 | 260.08238 | antiamebic, antiprotozoal |  |
| PIMOBENDAN | C19H18N4O2 | 334.38091 | PDE3 inhibitor, vasodilator, cardiotonic |  |
| VALGANCICLOVIR HYDROCHLORIDE | C14H23ClN6O5 | 390.82961 | antiviral |  |
| TAURINE | C2H7NO3S | 125.14699 | neuroprotectant, inhibitory neurotransmitter, GABA agonist |  |
| SUCRALFATE [5mM] | C12H54Al16O75S8 | 2086.73518 | antiulcer |  |
| CINACALCET HYDROCHLORIDE | C22H23ClF3N | 393.88351 | antihyperparathyroid |  |
| PIPERACETAZINE | C24H30N2O2S | 410.5829 | antipsychotic |  |
| PIROCTONE OLAMINE | C16H30N2O3 | 298.4291 | antiseborrheic |  |
| DOCUSATE SODIUM | C20H37NaO7S | 444.56749 | stool softener |  |
| RISEDRONATE SODIUM | C7H10NNaO7P2 | 305.09765 | calcium regulator |  |
| DABIGATRAN ETEXILATE MESYLATE | C35H45N7O8S | 723.855 | antithrombotic, serine protease inhibitor |  |
| ALBENDAZOLE | C12H15N3O2S | 265.33625 | anthelmintic |  |
| COTININE | C10H12N2O | 176.21994 | antidepressant |  |
| TYLOXAPOL | C70H124O13 | 1173.76098 | polymeric nonionic detergent |  |
| FLUROTHYL | C4H4F6O | 182.06628 | central stimulant, convulsant |  |
| BETAMETHASONE VALERATE | C27H37FO6 | 476.59074 | glucocorticoid |  |
| EMTRICITABINE | C8H10FN3O3S | 247.2496 | antiviraLreverse transcriptase inhibitor |  |
| CABAZITAXEL | C45H57NO14 | 835.95434 | antineoplastic |  |
| ENTECAVIR MONOHYDRATE | C12H17N5O4 | 295.30039 |  |  |
| DAPOXETINE HYDROCHLORIDE | C21H24ClNO | 341.88453 | antidepressant |  |
| BENZONATATE | C30H53NO11 | 603.75701 | antitussive |  |
| ROLIPRAM | C16H21NO3 | 275.35067 | tranquilizer |  |
| OXALIPLATIN | C8H14N2O4Pt | 397.30178 | antineoplastic |  |
| PHTHALYLSULFACETAMIDE | C16H14N2O6S | 362.36378 | antibacterial |  |
| ENILCONAZOLE SULFATE | C14H16Cl2N2O5S | 395.26402 | antifungal |  |
| SEMUSTINE | C10H18ClN3O2 | 247.72686 | antineoplastic |  |
| AMINOGLUTETHIMIDE | C13H16N2O2 | 232.28467 | aromatase inhibitor, antineoplastic, testosterone suppressant |  |
| PALIPERIDONE | C23H27FN4O3 | 426.49504 | schizopheria therapy |  |
| ALOGLIPTIN BENZOATE | C25H27N5O4 | 461.52504 | anti-diabetic, DPP-4 inhibitor |  |
| TACROLIMUS | C44H69NO12 | 804.04003 | immune suppressant, antifungal |  |
| FLUCONAZOLE | C13H12F2N6O | 306.27699 | antifungal |  |
| RAMELTEON | C16H21NO2 | 259.35127 | melatonin receptor agonist |  |
| BROMPHENIRAMINE MALEATE | C20H23BrN2O4 | 435.32131 | H1 antihistamine |  |
| SIROLIMUS | C51H79NO13 | 914.19718 | immunosuppressant, antineoplastic |  |
| METHYLPREDNISOLONE SODIUM SUCCINATE | C26H33NaO8 | 496.53791 | glucocorticoid, antiinflammatory |  |
| ETHYLNOREPINEPHRINE HYDROCHLORIDE | C10H16ClNO3 | 233.69692 | bronchodilator |  |
| TENIPOSIDE | C32H32O13S | 656.66804 | antineoplastic |  |
| CANDICIDIN | C59H84N2O18 | 1109.32993 | antifungal |  |
| TANNIC ACID | C76H52O46 | 1701.23424 | nonspecific enzyme/receptor blocker |  |
| TEPOXALIN | C20H20ClN3O3 | 385.8537 | antipsoratic |  |
| AZILSARTAN MEDOXOMIL | C30H23KN4O8 | 606.64181 | antihypertensive, AT1 angiotensin II antagonist |  |
| BUTOCONAZOLE | C19H17Cl3N2S | 411.78374 | antifungal |  |
| ACETRIAZOIC ACID | C9H6I3NO3 | 556.86627 | X-ray contrast medium |  |
| DIRITHROMYCIN | C42H78N2O14 | 835.09496 | antibacterial |  |
| MEPIVACAINE HYDROCHLORIDE | C15H23ClN2O | 282.81636 | anesthetic (local) |  |
| MELOXICAM | C14H13N3O4S2 | 351.40541 | antiinflammatory |  |
| NILUTAMIDE | C12H10F3N3O4 | 317.2264 | antiandrogen |  |
| VENLAFAXINE HYDROCHLORIDE | C17H28ClNO2 | 313.87121 | antidepressant |  |
| CITALOPRAM HYDROBROMIDE | C20H22BrFN2O | 405.31354 | antidepressant, 5HT reuptake inhibitor |  |
| FLUOXETINE HYDROCHLORIDE | C17H19ClF3NO | 345.79528 | antidepressant |  |
| BUPROPION | C13H19Cl2NO | 276.20848 | antidepressant |  |
| CEFUROXIME AXETIL | C20H22N4O10S | 510.48314 | antibacterial |  |
| FEXOFENADINE HYDROCHLORIDE | C32H40ClNO4 | 538.1329 | nonsedating H1-antihistamine |  |
| TRIFLURIDINE | C10H11F3N2O5 | 296.20477 | antiviral (opthalmic) |  |
| AMINOLEVULINIC ACID HYDROCHLORIDE | C5H10ClNO3 | 167.59335 | antineoplastic |  |
| DAPTOMYCIN (5 millimolar/DMSO) | C72H101N17O26 | 1620.70607 | antibacterial |  |
| AVOBENZONE | C20H22O3 | 310.39654 | sunscreen |  |
| ATOVAQUONE | C22H19ClO3 | 366.84793 | antipneumocystic, antimalarial |  |
| CHLOROGUANIDE HYDROCHLORIDE | C11H17Cl2N5 | 290.19764 | antimalarial |  |
| TRIMETOZINE | C14H19NO5 | 281.31123 | sedative, neurosedative |  |
| ACRISORCIN | C25H28N2O2 | 388.51411 | antifungal |  |
| CYSTEAMINE HYDROCHLORIDE | C2H8ClNS | 113.60976 | antiurolithic, depigmentation, radiation protectant |  |
| METAXALONE | C12H15NO3 | 221.25825 | muscle relaxant (skeletal) |  |
| CLARITHROMYCIN | C38H69NO13 | 747.97253 | antibacterial |  |
| DOBUTAMINE HYDROCHLORIDE | C18H24ClNO3 | 337.84988 | cardiotonic |  |
| SIMVASTATIN | C25H38O5 | 418.57861 | antihyperlipidemic, HMGCoA reductase inhibitor |  |
| HYDROQUINONE | C6H6O2 | 110.11352 | depigmentor, antioxidant |  |
| OXCARBAZEPINE | C15H12N2O2 | 252.27509 | antipsychotic |  |
| CARVEDILOL | C24H26N2O4 | 406.48582 | beta-adrenergic blocker |  |
| NATEGLINIDE | C19H27NO3 | 317.43194 | antidiabetic |  |
| IRBESARTAN | C25H28N6O | 428.54151 | angiotensin 2 receptor antagonist |  |
| LEVOFLOXACIN | C18H20FN3O4 | 361.3762 | antibacterial |  |
| CANDESARTAN CILEXTIL | C33H34N6O6 | 610.67553 | angiotensin 1 receptor antagonist |  |
| EPLERENONE | C24H30O6 | 414.5031 | antihypertensive, aldosterone antagonist |  |
| LOSARTAN | C22H23ClN6O | 422.92121 | antihypertensive, AT1 angiotensin II antagonist |  |
| LITHIUM CITRATE HYDRATE | C6H7Li3O8 | 227.93489 | antidepressant |  |
| MIGLITOL | C8H17NO5 | 207.22839 | alpha-glucosidase inhibitor, antidiabetic |  |
| DESVENLAFAXINE SUCCINATE | C20H31NO6 | 381.47317 | antidepressant |  |
| DEXLANSOPRAZOLE | C16H14F3N3O2S | 369.36808 | antiulcer, proton pump inhibitor |  |
| ARMODAFINIL | C15H15NO2S | 273.3563 | analeptic |  |
| NAFTIFINE HYDROCHLORIDE | C21H22ClN | 323.86919 | antifungal |  |
| ORLISTAT | C29H53NO5 | 495.74946 | reversible lipase inhibitor, antiobesity |  |
| MOXIFLOXACIN HYDROCHLORIDE | C21H25ClFN3O4 | 437.9025 | antibacterial |  |
| ENROFLOXACIN | C19H22FN3O3 | 359.40389 | antibacterial |  |
| HEXYLENE GLYCOL | C6H14O2 | 118.17728 | humectant |  |
| PIOGLITAZONE HYDROCHLORIDE | C19H21ClN2O3S | 392.90782 | antidiabetic |  |
| DONEPEZIL HYDROCHLORIDE | C24H30ClNO3 | 415.9646 | acetylcholinesterase inhibitor (reversible), cognitive enhancer |  |
| CARPROFEN | C15H12ClNO2 | 273.72139 | antiinflammatory, analgesic |  |
| FENOFIBRIC ACID | C17H15ClO4 | 318.7597 | antihyperlipidemic |  |
| CARVEDILOL PHOSPHATE | C24H29N2O8P | 504.48113 | antianginal, antihypertensive |  |
| VILAZODONE HYDROCHLORIDE | C26H28ClN5O2 | 477.99836 | antidepressant, serotonin reuptake inhibitor, 5HT1A agonist |  |
| TRILOSTANE | C20H27NO3 | 329.44309 | adrenocortical suppressant, antineoplastic, steroid biosynthesis inhibitor |  |
| MYCOPHENOLATE MOFETIL | C23H31NO7 | 433.50602 | immune suppressant, antineoplastic, antiviral |  |
| MILNACIPRAN HYDROCHLORIDE | C15H23ClN2O | 282.81636 | inhibitor of norepinephrine and seritonin uptake, treatment of fibromyalgia |  |
| IBANDRONATE SODIUM | C9H24NNaO8P2 | 359.23093 | bone resorption inhibitor, anthypercalcemic |  |
| AZITHROMYCIN | C38H72N2O12 | 749.00374 | antibacterial |  |
| MEPHENTERMINE SULFATE | C11H19NO4S | 261.34238 | vasoconstrictor |  |
| CEFTIOFUR HYDROCHLORIDE | C19H18ClN5O7S3 | 560.02961 | antibacterial |  |
| LETROZOLE | C17H11N5 | 285.31072 | antineoplastic |  |
| CLOSANTEL | C22H14Cl2I2N2O2 | 663.08388 | anthelmintic |  |
| ARGININE HYDROCHLORIDE | C6H15ClN4O2 | 210.66505 | ammonia detoxicant, diagnostic aid |  |
| OSELTAMIVIR PHOSPHATE | C16H31N2O8P | 410.40787 | antiviral, neraminidase inhibitor |  |
| LEVOCETIRIZINE DIHYDROCHLORIDE | C21H27Cl3N2O3 | 461.81994 | H1 antihistamine, antiallergic |  |
| GLIMEPIRIDE | C24H34N4O5S | 490.62638 | hypoglycemic, antidiabetic |  |
| OCTINOXATE | C18H26O3 | 290.40612 | sunscreen, UV-B absorber |  |
| CEPHALOTHIN SODIUM | C16H15N2NaO6S2 | 418.42555 | antibacterial |  |
| FLUCYTOSINE | C4H4FN3O | 129.09438 | antifungal |  |
| LEVALBUTEROL HYDROCHLORIDE | C13H22ClNO3 | 275.77819 | bronchodilator, tocolytic |  |
| PROPOFOL | C12H18O | 178.27666 | anesthetic |  |
| ETIDRONATE DISODIUM | C2H6Na2O7P2 | 249.99312 | bone resorption inhibitor |  |
| ARTEMETHER | C16H26O5 | 298.38262 | antimalarial |  |
| LOXAPINE SUCCINATE | C22H24ClN3O5 | 445.90668 | antipsychotic |  |
| CALCIUM CHLORIDE | CaCl2 | 110.986 | calcium replenisher |  |
| THIOSTREPTON | C72H85N19O18S5 | 1664.91675 | antibacterial |  |
| TILMICOSIN | C46H80N2O13 | 869.1561 | antibacterial |  |
| DESLORATADINE HYDROCHLORIDE | C19H20Cl2N2 | 347.29065 | H1-antihistamine |  |
| CLIOQUINOL | C9H5ClINO | 305.5037 | antiseptic, antiamebic |  |
| SALICYLANILIDE | C13H11NO2 | 213.23812 | antipyretic, fungicide |  |
| ESTROPIPATE | C22H32N2O5S | 436.57474 | estrogen |  |
| AZTREONAM | C13H17N5O8S2 | 435.43714 | antibiotic |  |
| CLAVULANATE LITHIUM | C8H10LiNO5 | 207.1116 | beta-lactamase inhibitor; antibiotic |  |
| TIZANIDINE HYDROCHLORIDE | C9H9Cl2N5S | 290.17558 | muscle relaxant, a-2 adrenergic agonist |  |
| FLUVASTATIN SODIUM | C24H25FNNaO4 | 433.45935 | antihyperlipidemic, HMGCoA reductase inhibitor |  |
| PANTOPRAZOLE | C16H15F2N3O4S | 383.37645 | proton pump inhibitor, gastric acid release inhibitor, antiulcer |  |
| QUETIAPINE FUMARATE | C25H29N3O6S | 499.59038 | antipsychotic: 5HT antagonist, dopamine antagonist, H1-antihistamine, alpha adrenergic blocker |  |
| BEMOTRIZINOL | C38H49N3O5 | 627.83133 | sunscreen |  |
| DRONEDARONE HYDROCHLORIDE | C31H45ClN2O5S | 593.2317 | antiarrhythmic |  |
| FAMCICLOVIR | C14H19N5O4 | 321.33863 | antiviral |  |
| AMLODIPINE BESYLATE | C26H31ClN2O8S | 567.06257 | Ca channel blocker |  |
| EZETIMIBE | C24H21F2NO3 | 409.43667 | sterol absorption inhibitor |  |
| NITRENDIPINE | C18H20N2O6 | 360.3699 | antihypertensive |  |
| OLMESARTAN MEDOXOMIL | C29H30N6O6 | 558.59905 | Angiotensin II inhibitor prodrug, antihypertensive |  |
| DICLORALUREA | C5H6Cl6N2O3 | 354.83317 | antibacterial |  |
| CEFTIBUTEN | C15H14N4O6S2 | 410.43003 | antibacterial |  |
| CEFDINIR | C14H13N5O5S2 | 395.41821 | antibacterial |  |
| VALSARTAN | C24H29N5O3 | 435.53043 | Angiotensin II inhibitor, antihypertensive |  |
| ISOFLUPREDONE ACETATE | C23H29FO6 | 420.48238 | antiinflammatory |  |
| TORSEMIDE | C16H20N4O3S | 348.4268 | diuretic, inhibits Na/K/2Cl carrier system |  |
| PERINDOPRIL ERBUMINE | C23H43N3O5 | 441.61626 | antihypertensive, ACE inhibitor |  |
| ROSUVASTATIN CALCIUM | C22H27CaFN3O6S | 520.61939 | antihyperlipidemic |  |
| RAMIPRIL | C23H32N2O5 | 416.52189 | antihypertensive, ACE inhibitor |  |
| TEGASEROD MALEATE | C20H27N5O5 | 417.46869 | 5HT4 receptor agonist, peristaltic stimulant |  |
| ESCITALOPRAM OXALATE | C22H23FN2O5 | 414.43741 | antidepressant, 5HT reuptake inhibitor |  |
| DERACOXIB | C17H14F3N3O3S | 397.37863 | antiinflammatory, antiarthritic, COX-2 inhibitor |  |
| CILOSTAZOL | C20H27N5O2 | 369.47049 | phosphodiesterase inhibitor |  |
| CANRENONE | C22H28O3 | 340.46666 | aldosterone antagonist; antifibrogenic |  |
| APRAMYCIN SULFATE | C21H43N5O15S | 637.66536 | antibacterial; LD50(iv) 280mg/kg(mouse) |  |
| HYDROXYCHLOROQUINE SULFATE | C18H28ClN3O5S | 433.95796 | antimalarial, lupus suppressant |  |
| TELMISARTAN | C33H30N4O2 | 514.63265 | antihypertensive, angiotensin II blocker |  |
| SERTRALINE HYDROCHLORIDE | C17H18Cl3N | 342.69871 | antidepressant, 5HT uptake inhibitor |  |
| ALFUZOSIN HYDROCHLORIDE | C19H28ClN5O4 | 425.91911 | alpha(1)-adrenergic blocker |  |
| TRANDOLAPRIL | C24H34N2O5 | 430.54898 | antihypertensive, ACE inhibitor |  |
| TELITHROMYCIN | C43H65N5O10 | 812.025 | antibacterial |  |
| OXAPROZIN | C18H15NO3 | 293.32515 | antiinflammatory |  |
| PROPRANOLOL HYDROCHLORIDE (+/-) | C16H22ClNO2 | 295.81224 | antihypertensive, antianginal, antiarrhythmic |  |
| CROTAMITON | C13H17NO | 203.28654 | antipruritic, scabicide |  |
| NONOXYNOL-9 | C33H60O10 | 616.84015 | spermatocide, contraceptive |  |
| CARBADOX | C11H10N4O4 | 262.22675 | antibacterial |  |
| OXFENDAZOLE | C15H13N3O3S | 315.35316 | anthelmintic |  |
| AMITRAZ | C19H23N3 | 293.41526 | scabicide |  |
| ASPARTAME | C14H18N2O5 | 294.30996 | sweetener |  |
| BIFONAZOLE | C22H18N2 | 310.40216 | antifungal, calmodulin antagonist |  |
| TYLOSIN TARTRATE | C50H83NO23 | 1066.21191 | antibacterial |  |
| SARAFLOXACIN HYDROCHLORIDE | C20H18ClF2N3O3 | 421.83456 | antibacterial |  |
| CLOPIDOL | C7H7Cl2NO | 192.04594 | coccidiostat, antiplatelet |  |
| RIFAXIMIN | C43H51N3O11 | 785.89942 | antibacterial, RNA synthesis inhibitor |  |
| CHLORMADINONE ACETATE | C23H29ClO4 | 404.93818 | progestin, antiandrogen |  |
| BENZTROPINE MESYLATE | C22H29NO4S | 403.54473 | anticholinergic, antiparkinsonian |  |
| LINAGLIPTIN | C25H30N8O2 | 474.57025 | DPP-4 inhibitor, antidiabetic |  |
| PAROXETINE HYDROCHLORIDE | C19H21ClFNO3 | 365.83552 | antidepressant |  |
| AZELASTINE HYDROCHLORIDE | C22H25Cl2N3O | 418.37005 | H1 antihistamine (nonsedating); leucotriene synthesis blocker |  |
| KETANSERIN | C22H22FN3O3 | 395.43734 | 5HT2/5HT1C seritonin antagonist |  |
| RIBOFLAVIN | C17H20N4O6 | 376.37215 | Vitamin B2; Vitamin cofactor; LD50(rat) 560 mg/kg ip |  |
| SUVOREXANT | C23H23ClN6O2 | 450.93176 | CNS deppresant; orexin receptor antagonist; treatment of insomnia |  |
| CEFDITORIN PIVOXIL | C25H28N6O7S3 | 620.72991 | antibacterial |  |
| MODAFINIL | C15H15NO2S | 273.3563 | analeptic |  |
| CEFPROZIL | C18H19N3O5S | 389.43323 | antibacterial |  |
| RANOLAZINE DIHYDROCHLORIDE | C24H35Cl2N3O4 | 500.47025 | antianginal, antiischemic |  |
| MOMETASONE FUROATE | C27H30Cl2O6 | 521.44255 | antiinflammatory, glucocorticoid |  |
| VALACYCLOVIR HYDROCHLORIDE | C13H21ClN6O4 | 360.80312 | antiviral |  |
| ZOLPIDEM | C19H21N3O | 307.39872 | sedative, hypnotic |  |
| CETIRIZINE HYDROCHLORIDE | C21H27Cl3N2O3 | 461.81994 | H1 antihistamine |  |
| SUMATRIPTAN | C14H21N3O2S | 295.40637 | 5HT agonist, antimigraine |  |
| VARDENAFIL HYDROCHLORIDE | C23H33ClN6O4S | 525.07426 | erectile dysfunction, PD5 inhibitor |  |
| ACEDAPSONE | C16H16N2O4S | 332.38092 | antimalarial, leprostatic |  |
| ATOMOXETINE HYDROCHLORIDE | C17H22ClNO | 291.82399 | norepinephrine reuptake inhibitor |  |
| PRAMIPEXOLE DIHYDROCHLORIDE | C10H19Cl2N3S | 284.25303 | antidepressant, dopamine antagonist |  |
| PEMIROLAST POTASSIUM | C10H7KN6O | 266.30889 | antiallergic |  |
| TRAMADOL HYDROCHLORIDE | C16H26ClNO2 | 299.84412 | analgesic |  |
| NISOLDIPINE | C20H24N2O6 | 388.42408 | vasodilator (coronary) |  |
| PARAMETHADIONE | C7H11NO3 | 157.17062 | anticonvulsant |  |
| TERBINAFINE HYDROCHLORIDE | C21H26ClN | 327.90107 | antifungal |  |
| DESLORATIDINE | C19H19ClN2 | 310.82968 | H1-antihistamine |  |
| MOXIDECTIN | C37H53NO8 | 639.83686 | anthelmintic, antiparasitic |  |
| FLUORESCEIN | C20H12O5 | 332.31564 | corneal trama indicator |  |
| NIACINAMIDE | C6H6N2O | 122.12752 | Vitamin B3; enzyme cofactor; anti-pellagra |  |
| PHENYLETHYL ALCOHOL | C8H10O | 122.1683 | antimicrobial |  |
| OXYBUTYNIN CHLORIDE | C22H32ClNO3 | 393.95824 | anticholinergic |  |
| BENZOXIQUINE | C16H11NO2 | 249.27157 | antiinfective |  |
| BENURESTAT | C9H9ClN2O3 | 228.63668 | urease inhibitor |  |
| DECOQUINATE [5mM] | C24H35NO5 | 417.55025 | coccidiostat |  |
| BISMUTH SUBSALICYLATE | C7H5BiO4 | 362.0955 | antidiarrheal, antacid, antiulcer |  |
| BENZOYLPAS | C14H11NO4 | 257.24807 | antibacterial, tuberculostatic |  |
| BROMINDIONE | C15H9BrO2 | 301.14178 | anticoagulant |  |
| BURAMATE | C10H13NO3 | 195.22001 | anticonvulsant, antipsychotic |  |
| CAPOBENIC ACID | C16H23NO6 | 325.36481 | antiarrhythmic |  |
| DEXPANTHENOL | C9H19NO4 | 205.25608 | cholinergic |  |
| ETHOPABATE | C12H15NO4 | 237.25765 | folate metabolic inhibitor, coccidiostat |  |
| BENDAZAC | C16H14N2O3 | 282.30158 | antiinflammatory |  |
| ETHOXZOLAMIDE | C9H10N2O3S2 | 258.31965 | carbonic anhydrase inhibitor, antiulcer, antiglaucoma |  |
| BISOCTRIZOLE | C41H50N6O2 | 658.89465 | sunscreen |  |
| FOSINOPRIL SODIUM | C30H45NNaO7P | 585.65925 | ACE inhibitor, antihypertensive |  |
| DEFLAZACORT | C25H31NO6 | 441.52892 | antiinflammatory |  |
| FOMEPIZOLE HYDROCHLORIDE | C4H7ClN2 | 118.56679 | alcohol dehydrogenase inhibitor, antidote |  |
| GLIPIZIDE | C21H27N5O4S | 445.54444 | antidiabetic |  |
| RAFOXANIDE | C19H11Cl2I2NO3 | 626.01922 | anthelmintic |  |
| GUANFACINE HYDROCHLORIDE | C9H10Cl3N3O | 282.55855 | antihypertensive |  |
| D-LACTITOL MONOHYDRATE | C12H26O12 | 362.33382 | sweetener, treatment of portoencephalopathy |  |
| ESTRAMUSTINE | C23H31Cl2NO3 | 440.41442 | antineoplastic |  |
| HYDROCORTISONE VALERATE | C26H38O6 | 446.58916 | antiinflammatory, glucocorticoid |  |
| LOBENDAZOLE | C10H11N3O2 | 205.21807 | anthelmintic |  |
| METHSUXIMIDE | C12H13NO2 | 203.24291 | anticonvulsant |  |
| METHYLENE BLUE | C16H20ClN3OS | 337.8743 | antimethemoglobinemic, cyanide antidote |  |
| METHYLATROPINE NITRATE | C18H26N2O6 | 366.41772 | anticholinergic |  |
| LYNESTRENOL | C20H28O | 284.44556 | progestin |  |
| MINOXIDIL | C9H15N5O | 209.2528 | antihypertensive, antialopecia agent |  |
| NITHIAMIDE | C5H5N3O3S | 187.1779 | antibacterial |  |
| PRALIDOXIME CHLORIDE | C7H9ClN2O | 172.61558 | cholesterase agonist |  |
| PREDNISOLONE HEMISUCCINATE | C25H32O8 | 460.52899 | antiinflammatory, glucocorticoid |  |
| CAPECITABINE | C15H22FN3O6 | 359.35749 | antineoplastic |  |
| PYRIDOXINE HYDROCHLORIDE | C8H12ClNO3 | 205.64274 | vitamin B6, enzyme cofactor |  |
| PHENSUCCIMIDE | C11H11NO2 | 189.21582 | anticonvulsant |  |
| RIMANTADINE HYDROCHLORIDE | C12H22ClN | 215.76884 | antiviral |  |
| SULFISOXAZOLE ACETYL | C13H15N3O4S | 309.3462 | antibacterial |  |
| SULISOBENZONE | C14H12O6S | 308.31214 | ultraviolet screen |  |
| AMMONIUM LACTATE | C3H9NO3 | 107.11008 | antipruritic |  |
| TRICLOSAN | C12H7Cl3O2 | 289.54739 | antiinfective |  |
| TRIMETHADIONE | C6H9NO3 | 143.14353 | anticonvulsant |  |
| UNDECYLENIC ACID | C11H20O2 | 184.28085 | antifungal |  |
| DISOPYRAMIDE PHOSPHATE | C21H32N3O5P | 437.48009 | antiarrhythmic |  |
| CEFONICID SODIUM | C18H16N6Na2O8S3 | 586.53522 | antibacterial |  |
| IFOSFAMIDE | C7H15Cl2N2O2P | 261.0896 | antineoplastic |  |
| DOXORUBICIN | C27H29NO11 | 543.53228 | antineoplastic |  |
| CLOZAPINE | C18H19ClN4 | 326.83193 | antipsychotic |  |
| MOEXIPRIL HYDROCHLORIDE | C27H35ClN2O7 | 535.0422 | antihypertensive, ACE inhibitor |  |
| HYDRALAZINE HYDROCHLORIDE | C8H9ClN4 | 196.64073 | antihypertensive |  |
| SOLIFENACIN SUCCINATE | C27H32N2O6 | 480.56589 | muscarinic M3 antagonist |  |
| PHENOBARBITAL | C12H12N2O3 | 232.24104 | sedative, hypnotic, central depressant |  |
| OLANZAPINE | C17H20N4S | 312.43975 | antipsychotic |  |
| BENOXINATE HYDROCHLORIDE | C17H29ClN2O3 | 344.88528 | anesthetic (local) |  |
| CEFIXIME | C16H15N5O7S2 | 453.45525 | antibacterial |  |
| GLYBURIDE | C23H28ClN3O5S | 494.01371 | antihyperglycemic |  |
| NEOSTIGMINE METHYLSULFATE | C13H22N2O6S | 334.39409 | cholinergic |  |
| CITICOLINE | C14H26N4O11P2 | 488.33112 | cognition enhancer, phosphocholine cytidyltransferase activator |  |
| DILOXANIDE FUROATE | C14H11Cl2NO4 | 328.15407 | amoebicide |  |
| NADOLOL | C17H27NO4 | 309.40904 | betaadrenergic blocker |  |
| DYDROGESTERONE | C21H28O2 | 312.45611 | progestin | IC50 = 10 µM |
| FENOLDOPAM MESYLATE | C17H20ClNO6S | 401.86905 | antihypertensive, dopamine agonist |  |
| FESOTERODINE FUMARATE | C30H41NO7 | 527.66377 | muscarinic antagonist |  |
| CEFPIRAMIDE | C25H24N8O7S2 | 612.64743 | antibacterial |  |
| TERAZOSIN HYDROCHLORIDE | C19H26ClN5O4 | 423.90317 | antihypertensive |  |
| QUINAPRIL | C25H30N2O5 | 438.52825 | antihypertensive, ACE inhibitor |  |
| CHLOROTHALONIL | C8Cl4N2 | 265.9146 | antifungal, pesticide, acaricide |  |
| GENTAMICIN SULFATE | C21H45N5O11S | 575.6837 | antibacterial |  |
| DROXYDOPA | C9H11NO5 | 213.19172 | neurogenic hypotension, antiparkinsonian |  |
| GUANETHIDINE MONOSULFATE | C10H24N4O4S | 296.39118 | antihypertensive, mitotic agent |  |
| GLYCERIN | C3H8O3 | 92.09541 | pharmaceutical aid |  |
| ANAGRELIDE HYDROCHLORIDE | C10H8Cl3N3O | 292.55376 | antithrombotic |  |
| FLUOROURACIL | C4H3FN2O2 | 130.07911 | antineoplastic, pyrimidine antimetabolite |  |
| ETOMIDATE | C14H16N2O2 | 244.29582 | sedative |  |
| FLORFENICOL | C12H14Cl2FNO4S | 358.21808 | antibacterial |  |
| FLUVOXAMINE MALEATE | C19H25F3N2O6 | 434.4161 | antidepressant, antiobsessional agent |  |
| LAMOTRIGINE | C9H7Cl2N5 | 256.09564 | anticonvulsant |  |
| ROCURONIUM BROMIDE | C32H53BrN2O4 | 609.69421 | neuromuscular blocker |  |
| TILETAMINE HYDROCHLORIDE | C12H18ClNOS | 259.80036 | anesthetic, anticonvulsant |  |
| CEFPODOXIME PROXETIL | C21H27N5O9S2 | 557.60544 | antibacterial |  |
| TADALAFIL | C22H19N3O4 | 389.41443 | erectile dysfunction therapy |  |
| ETHYL VANILLIN | C9H10O3 | 166.17825 | flavoring agent |  |
| ARSANILIC ACID | C6H8AsNO3 | 217.05716 | antibacterial |  |
| PANTHENOL (dl) | C9H19NO4 | 205.25608 | vitamin B5 precursor, radioprotectant, antimalarial |  |
| ANIRACETAM | C12H13NO3 | 219.24231 | cognitive enhancer |  |
| VINCRISTINE SULFATE | C46H58N4O14S | 923.05756 | antineoplastic |  |
| TRIENTINE HYDROCHLORIDE | C6H20Cl2N4 | 219.1591 | chelating agent |  |
| TICLOPIDINE HYDROCHLORIDE | C14H15Cl2NS | 300.25235 | PAF inhibitor |  |
| TICARCILLIN DISODIUM | C15H14N2Na2O6S2 | 428.39623 | antibacterial |  |
| TETRAMIZOLE HYDROCHLORIDE | C11H13ClN2S | 240.75666 | anthelmintic |  |
| TOLTRAZURIL | C18H14F3N3O4S | 425.38918 | coccidiostat |  |
| TIBOLONE | C21H28O2 | 312.45611 | menopausal syndrome therapy |  |
| ANTIMONY POTASSIUM TARTRATE TRIHYDRATE | C8H10K2O15Sb2 | 667.8639 | antischistosomal |  |
| QUINAPRILAT | C23H26N2O5 | 410.47407 | antihypertensive, ACE inhiibitor |  |
| DORZOLAMIDE | C10H17ClN2O4S3 | 360.90299 | carbonic anhydrase inhibitor |  |
| PIPAMPERONE | C21H30FN3O2 | 375.49055 | antipsychotic |  |
| PEFLOXACIN MESYLATE | C18H24FN3O6S | 429.47088 | antibacterial |  |
| NETILMICIN SULFATE | C21H45N5O15S2 | 671.7453 | antibacterial |  |
| OMEPRAZOLE | C17H19N3O3S | 345.42328 | gastric acid depressant |  |
| FlUMAZENIL | C15H14FN3O3 | 303.29553 | benzodiazepine antagonist |  |
| ALTRENOGEST | C21H26O2 | 310.44017 | progestin, antineoplastic |  |
| FINASTERIDE | C23H36N2O2 | 372.55557 | anti-androgen, alpha-reductase inhibitor |  |
| FLUDARABINE PHOSPHATE | C10H13FN5O7P | 365.21661 | antineoplastic |  |
| MUPIROCIN | C26H44O9 | 500.63518 | antibacterial, antimycoplasmal, isoleucyl-tRNA synthetase inhibitor |  |
| TEICOPLANIN [A(2-1) shown] | C88H95Cl2N9O33 | 1877.68485 | antibacterial |  |
| EPIRUBICIN HYDROCHLORIDE | C27H30ClNO11 | 579.99325 | antineoplastic |  |
| VECURONIUM BROMIDE | C34H57BrN2O4 | 637.74839 | neuromuscular blocker |  |
| ACAMPROSATE CALCIUM | C10H20CaN2O8S2 | 400.4875 | alcohol antagonist |  |
| PREDNISOLONE SODIUM PHOSPHATE | C21H27Na2O8P | 484.39794 | antiinflammatory, glucocorticoid |  |
| PREGNENOLONE SUCCINATE | C25H36O5 | 416.56267 | glucocortcoid, antiinflammatory |  |
| EMEDASTINE DIFUMARATE | C25H34N4O9 | 534.57113 | antihistamine |  |
| MEGLUMINE | C7H17NO5 | 195.21724 | diagnostic aid |  |
| RETINYL PALMITATE | C36H60O2 | 524.8784 | provitamin, antixerophthalamic |  |
| CHLOROPHYLLIDE Cu COMPLEX Na SALT | C34H33CuN4Na3O6 | 726.18071 | antineoplastic |  |
| DESOXYMETASONE | C22H29FO4 | 376.47243 | antiinflammatory |  |
| BETAMETHASONE ACETATE | C24H31FO6 | 434.50947 | antiinflammatory |  |
| BETAMETHASONE SODIUM PHOSPHATE | C22H28FNa2O8P | 516.41546 | antiinflammatory, glucocorticoid |  |
| NATAMYCIN | C33H47NO13 | 665.74144 | antibacterial |  |
| DESONIDE | C24H32O6 | 416.51904 | antiinflammatory, glucocorticoid |  |
| MELENGESTROL ACETATE | C25H32O4 | 396.53139 | antineoplastic, progestin |  |
| ENTACAPONE | C14H15N3O5 | 305.29275 | antidyskinetic |  |
| MIRTAZAPINE | C17H19N3 | 265.36108 | serotonergic antidepressant, anxiolytic, antiemetic |  |
| ASCORBYL PALMITATE | C22H38O7 | 414.54396 | antioxidant |  |
| ERYTHROSINE SODIUM | C20H6I4Na2O5 | 879.86502 | color additive |  |
| GLYCOPYRROLATE | C19H28BrNO3 | 398.34391 | anticholinergic |  |
| BEPRIDIL HYDROCHLORIDE | C24H35ClN2O | 403.01235 | antiarrhythmic |  |
| OCTISALATE | C15H22O3 | 250.34079 | sunscreen |  |
| TRICHLORFON | C4H8Cl3O4P | 257.43876 | anthelmintic |  |
| DIATRIZOIC ACID | C11H9I3N2O4 | 613.91858 | radiopaque agent |  |
| BEPHENIUM HYDROXYNAPTHOATE | C28H29NO4 | 443.54763 | anthelmintic |  |
| CITRIC ACID | C6H8O7 | 192.12646 |  |  |
| DEXRAZOXANE | C11H16N4O4 | 268.27457 | cardioprotectant |  |
| TOPIRAMATE | C12H21NO8S | 339.36707 | anticonvulsant, antimigraine, GABA-A agonist, AMP/kinate glutamate receptor antagonist, carbonic anhydrase inhibitor |  |
| GEMIFLOXACIN MESYLATE | C19H24FN5O7S | 485.49483 | antibacterial |  |
| PRAVASTATIN SODIUM | C23H35NaO7 | 446.521 | antihyperlipidemic, HMGCoA reductase inhibitor |  |
| GABAPENTIN | C9H17NO2 | 171.24134 | anticonvulsant |  |
| ALISKIREN HEMIFUMARATE | C34H57N3O10 | 667.84749 | renin inhibitor |  |
| METFORMIN HYDROCHLORIDE | C4H12ClN5 | 165.62674 | antidiabetic |  |
| CEFSULODIN SODIUM | C22H19N4NaO8S2 | 554.53653 | antibacterial |  |
| CREATININE | C4H7N3O | 113.11989 | metabolic enhancer |  |
| LOVASTATIN | C24H36O5 | 404.55152 | antihyperlipidemic, HMGCoA reductase inhibitor |  |
| PROPIOLACTONE | C3H4O2 | 72.06413 | antiinfective |  |
| FEBUXOSTAT | C16H16N2O3S | 316.38152 | xanthine oxidase/dehydrogenase inhibitor |  |
| SITAGLIPTIN PHOSPHATE | C16H20F6N5O6P | 523.3319 | DPP4 inhibitor, antidiabetic |  |
| ZINC UNDECYLENATE [4mM] | C22H38O4Zn | 431.91576 | antifungal |  |
| PIRENPERONE | C23H24FN3O2 | 393.46503 | 5HT2 receptor antagonist |  |
| ATAZANAVIR SULFATE | C38H54N6O11S | 802.95168 | antiviral, HIV protease inhibitor |  |
| METHYCLOTHIAZIDE | C9H11Cl2N3O4S2 | 360.23972 | antihypertensive |  |
| ATRACURIUM BESYLATE | C65H82N2O18S2 | 1243.50889 | neuromuscular blocker |  |
| EXEMESTANE | C20H24O2 | 296.41308 | antineoplastic, aromatase inhibitor | IC50 = 10 µM |
| D-ERYTHOBIC ACID | C6H8O6 | 176.12706 | antioxidant |  |
| ATROPINE | C17H23NO3 | 289.37776 | anticholinergic |  |
| ZILEUTON | C11H12N2O2S | 236.29449 | 5-lipoxygenase inhibitor |  |
| METHYLPHENIDATE HYDROCHLORIDE | C14H20ClNO2 | 269.774 | CNS stimulant |  |
| ZALEPLON | C17H15N5O | 305.342 | sedative, hypnotic |  |
| RABEPRAZOLE SODIUM | C18H20N3NaO3S | 381.4322 | gastric acid secretion inhibitor |  |
| PAMABROM | C7H7BrN4O2 | 259.06344 | diuretic |  |
| COLISTIN SULFATE | C52H102N16O21S2 | 1351.61534 | antibacterial |  |
| ARSENIC TRIOXIDE DIETHANOLAMINE SALT | C4H18As2N2O5 | 324.04166 | antineoplastic, antileukemia |  |
| BENZBROMARONE | C17H12Br2O3 | 424.09139 | uricosuric |  |
| BROMPERIDOL | C21H23BrFNO2 | 420.32536 | antipsychotic |  |
| CYPROHEPTADINE HYDROCHLORIDE | C21H22ClN | 323.86919 | H1-antihistamine, antipruritic |  |
| CLOFAZIMINE | C27H22Cl2N4 | 473.40919 | antibacterial, antilepretic, antituberculosis |  |
| BENZYDAMINE HYDROCHLORIDE | C19H24ClN3O | 345.87563 | analgesic, antipyretic, antiinflammatory |  |
| DOXAZOSIN MESYLATE | C24H29N5O8S | 547.59143 | antihypertensive |  |
| ISOETHARINE MESYLATE | C14H25NO6S | 335.42245 | bronchodilator |  |
| FAMPRIDINE | C5H6N2 | 94.11697 | K channel blocker; multiple sclerosis therapy |  |
| ETHYNODIOL DIACETATE | C24H32O4 | 384.52024 | progestin |  |
| ORNIDAZOLE | C7H10ClN3O3 | 219.62905 | antiinfective |  |
| OXANTEL PAMOATE | C36H32N2O7 | 604.66564 | anthelmintic |  |
| DIBUTYL PHTHALATE | C16H22O4 | 278.35134 | plasticiser, suspect endocrine disruptor |  |
| PROTRYPTYLINE HYDROCHLORIDE | C19H22ClN | 299.84689 | antidepressant |  |
| NIZATIDINE | C12H21N5O2S2 | 331.46147 | antiulcer, H2 antagonist |  |
| DENATONIUM BENZOATE | C28H34N2O3 | 446.59478 | denaturing agent, bitter principle |  |
| DECAMETHONIUM BROMIDE | C16H38Br2N2 | 418.30266 | neuromuscular blocker |  |
| MESALAMINE | C7H7NO3 | 153.13874 | antiinflammatory |  |
| ETHAMIVAN | C12H17NO3 | 223.27419 | CNS & respiratory stimulant |  |
| BUTYL PARABEN | C11H14O3 | 194.23243 | antifungal, preservative |  |
| ACETANILIDE | C8H9NO | 135.16703 | analgesic, antipyretic |  |
| METYRAPONE | C14H14N2O | 226.28048 | diagnostic aid |  |
| MOLINDONE HYDROCHLORIDE | C16H25ClN2O2 | 312.84285 | antipsychotic |  |
| NICORANDIL | C8H9N3O4 | 211.17863 | vasodilator |  |
| REPAGLINIDE | C27H36N2O4 | 452.59897 | antidiabetic |  |
| RISPERIDONE | C23H27FN4O2 | 410.49564 | neuroleptic |  |
| SOTALOL HYDROCHLORIDE | C12H21ClN2O3S | 308.82977 | beta-adrenergic agonist |  |
| CYCLOSERINE (L) | C3H6N2O2 | 102.09347 | 3-ketodihydrosphingosine synthetase inhibitor, antiviral |  |
| BENAZEPRIL HYDROCHLORIDE | C24H29ClN2O5 | 460.96213 | ACE inhibitor, antihypertensive |  |
| IODOQUINOL | C9H5I2NO | 396.9551 | antiamebic |  |
| DARIFENACIN HYDROBROMIDE | C28H31BrN2O2 | 507.47547 | M3 muscarinic antagonist, bladder suppressant |  |
| BISOPROLOL FUMARATE | C22H35NO8 | 441.52615 | beta-blocker, antihypertensive |  |
| PENTETIC ACID | C14H23N3O10 | 393.35351 | chelating agent, diagnostic aid |  |
| SULFADOXINE | C12H14N4O4S | 310.33378 | antibacterial |  |
| PENTAGASTRIN | C37H49N7O9S | 767.90858 | gastric secretion indicator |  |
| PROTIRELIN | C16H22N6O4 | 362.39154 | prothyrotropin |  |
| ALLYLISOTHIOCYANATE | C4H5NS | 99.15515 | counterirritant |  |
| PHENFORMIN HYDROCHLORIDE | C10H16ClN5 | 241.72552 | antidiabetic |  |
| PANTOTHENIC ACID(d) Na salt | C9H16NNaO5 | 241.22137 | vitamin B5 |  |
| MODALINE SULFATE | C10H17N3O4S | 275.32869 | antidepressant, antisecretory |  |
| ADRENALONE HYDROCHLORIDE | C9H12ClNO3 | 217.65389 | adrenergic (opthalmic) |  |
| DIMETHYL FUMARATE | C6H8O4 | 144.12826 | immunomodulator |  |
| AMINOPENTAMIDE SULFATE | C19H26N2O5S | 394.49347 | antispasmodic, antiemetic |  |
| TEMOZOLOMIDE | C6H6N6O2 | 194.15372 | antineoplastic |  |
| FELBINAC | C14H12O2 | 212.25054 | antiinflammatory |  |
| VALETHAMATE BROMIDE | C19H32BrNO2 | 386.37639 | antispasmodic |  |
| ETHANOLAMINE OLEATE | C20H41NO3 | 343.55467 | sclerosing agent |  |
| MOLSIDOMINE | C9H14N4O4 | 242.23633 | antianginal |  |
| HYMECHROME | C10H8O3 | 176.17346 | choloretic, spasmolytic, sunscreen |  |
| PIMAGEDINE HYDROCHLORIDE | CH7ClN4 | 110.54674 | glycosylation inhibitor |  |
| ISOVALERAMIDE | C5H11NO | 101.14952 | anticonvulsant, antimigraine, analgesic |  |
| DETOMIDINE HYDROCHLORIDE | C12H15ClN2 | 222.71975 | analgesic, sedative |  |
| TIGECYCLINE | C29H39N5O8 | 585.66288 | antibacterial |  |
| EPRODISATE DISODIUM | C3H6Na2O6S2 | 248.18527 | amyloidosis therapy |  |
| IMEXON | C4H5N3O | 111.10395 | antineoplastic |  |
| PHYSOSTIGMINE SULFATE | C15H23N3O6S | 373.43106 | cholinergic, anticholinesterase, miotic |  |
| MEGLUTOL | C6H10O5 | 162.1436 | antihyperlipoproteinemia |  |
| SYMCLOSENE | C3Cl3N3O3 | 232.41075 | anti-infective |  |
| OCTOCRYLENE | C24H27NO2 | 361.48829 | sunscreen |  |
| TROCLOSENE SODIUM | C3Cl2N3NaO3 | 219.94755 | antiinfective |  |
| FLUNIXIN MEGLUMINE | C21H28F3N3O7 | 491.46841 | analgesic, antiinflammatory |  |
| ELETRIPTAN HYDROBROMIDE | C22H27BrN2O2S | 463.44069 | 5-HT agonist, anti-migrane |  |
| DIETHYLTOLUAMIDE | C12H17NO | 191.27539 | insect repellant |  |
| THIAMYLAL SODIUM | C12H17N2NaO2S | 276.33529 | anesthetic |  |
| THIOPENTAL SODIUM | C11H17N2NaO2S | 264.32414 | anesthetic |  |
| TEMAZEPAM | C16H13ClN2O2 | 300.74721 | sedative, minor tranquilizer |  |
| TAPENTADOL HYDROCHLORIDE | C14H24ClNO | 257.80648 | analgesic, norepinephrine uptake blocker, mu-opiod receptor agonist |  |
| TILORONE | C25H34N2O3 | 410.56133 | antiviral |  |
| CLOMIPRAMINE HYDROCHLORIDE | C19H24Cl2N2 | 351.32253 | antidepressant |  |
| CHLORMEZANONE | C11H12ClNO3S | 273.74019 | anxiolytic, muscle relaxant |  |
| AMOXAPINE | C17H16ClN3O | 313.78957 | antidepressant, inhibits norepinephrine uptake |  |
| TACRINE HYDROCHLORIDE | C13H15ClN2 | 234.7309 | anticholinesterase, cognitive adjuvant, K channel blocker |  |
| ACECAINIDE HYDROCHLORIDE | C15H24ClN3O2 | 313.83043 | antiarrhythmic |  |
| TOREMIFENE CITRATE | C32H36ClNO8 | 598.09862 | antineoplastic, anti-estrogen |  |
| PYRIDOSTIGMINE BROMIDE | C9H13BrN2O2 | 261.12016 | cholinergic |  |
| BUSPIRONE HYDROCHLORIDE | C21H32ClN5O2 | 421.97449 | 5HT1a receptor agonist, anxiolytic |  |
| VORTIOXETINE HYDROBROMIDE | C18H23BrN2S | 379.36541 | antidepressant |  |
| LEVODOPA | C9H11NO4 | 197.19232 | antiparkinsonian |  |
| DIAZOXIDE | C8H7ClN2O2S | 230.67419 | antihypertensive, diuretic, activates K channels and AMPA receptors |  |
| ITRACONAZOLE HYDROCHLORIDE | C35H40Cl4N8O4 | 778.57225 | antifungal |  |
| DILTIAZEM HYDROCHLORIDE | C22H27ClN2O4S | 450.98849 | Ca channel blocker, coronary vasodilator |  |
| EDROPHONIUM CHLORIDE | C10H16ClNO | 201.69812 | acetylcholinesterase inhibitor |  |
| OXOLINIC ACID | C13H10KNO5 | 299.33035 | antibacterial |  |
| DACTINOMYCIN | C62H86N12O16 | 1255.44752 | antineoplastic, intercalating agent |  |
| DEBRISOQUIN SULFATE | C10H15N3O4S | 273.31275 | anti-hypertensive |  |
| NICOTINE BITARTRATE | C18H26N2O12 | 462.41412 | nicotinyl acetylchloline receptor agonist, ectoparasiticide |  |
| VERAPAMIL HYDROCHLORIDE | C27H39ClN2O4 | 491.07588 | adrenegic blocker, Ca channel blocker, coronary vasodilator, antiarrhythmic |  |
| METOLAZONE | C16H16ClN3O3S | 365.84122 | diuretic, antihypertensive |  |
| PINACIDIL | C13H19N5 | 245.32988 | K channel agonist, antihypertensive |  |
| MIANSERIN HYDROCHLORIDE | C18H21ClN2 | 300.83447 | 5HT antagonist |  |
| DIDANOSINE | C10H12N4O3 | 236.23214 | antiviral |  |
| TRANEXAMIC ACID | C8H15NO2 | 157.21425 | hemostatic |  |
| QUINETHAZONE | C10H12ClN3O3S | 289.74244 | diuretic, antihypertensive |  |
| CLORSULON | C8H8Cl3N3O4S2 | 380.65766 | antiparasitic, fasciolicide |  |
| CAPTAMINE | C4H11NS | 105.20297 | depigmentor |  |
| TERPENE HYDRATE | C10H22O3 | 190.28504 | expectorant |  |
| SODIUM MONOFLUOROPHOSPHATE | FNa2O3P | 143.95 | dental carries prophylactic |  |
| TROSPIUM CHLORIDE | C25H30ClNO3 | 427.97575 | anticholinergic, urinary incontenance therapy |  |
| AMIKACIN HYDRATE | C22H45N5O14 | 603.62905 | antibacterial |  |
| PENICILLAMINE ETHANOLAMINE SALT | C7H18N2O3S | 210.29711 | chelating agent (Cu), antirheumatic |  |
| LINDANE | C6H6Cl6 | 290.83272 | pediculicide, insecticide |  |
| ACETOHYDROXAMIC ACID | C2H5NO2 | 75.06765 | urease inhibitor, antiurolithic, antbacterial |  |
| IOTHALAMIC ACID | C11H9I3N2O4 | 613.91858 | radiopaque agent |  |
| LUMEFANTRINE | C30H32Cl3NO | 528.95464 | antimalarial |  |
| ALPRENOLOL HYDROCHLORIDE | C15H24ClNO2 | 285.81703 | betaadrenergic blocker |  |
| ETHYL PARABEN | C9H10O3 | 166.17825 | antifungal |  |
| EDETATE DISODIUM | C10H16N2Na2O8 | 338.22722 | chelating agent |  |
| FLUTICASONE PROPIONATE | C25H31F3O5S | 500.58202 | antiinflammatory |  |
| AMIODARONE HYDROCHLORIDE | C25H30ClI2NO3 | 681.78455 | adrenergic agonist, coronary vasodilator, Ca channel blocker |  |
| NEVIRAPINE | C15H14N4O | 266.30503 | antiviral, RT inhibitor |  |
| EVANS BLUE | C34H24N6Na4O14S4 | 960.81738 | glutamate uptake inhibitor, AMPA blocker |  |
| LOPERAMIDE HYDROCHLORIDE | C29H34Cl2N2O2 | 513.51253 | Ca channel blocker |  |
| TRIAMCINOLONE ACETONIDE | C24H31FO6 | 434.50947 | antiinflammatory |  |
| PHENTOLAMINE HYDROCHLORIDE | C17H20ClN3O | 317.82145 | antihypertensive |  |
| ZOLMITRIPTAN | C16H21N3O2 | 287.36467 | antimigraine, 5HT[1B/1D] agonist |  |
| RIFAPENTINE | C47H64N4O12 | 877.05373 | antibacterial |  |
| IVERMECTIN | C48H74O14 | 875.11658 | antiparasitic |  |
| TROMETHAMINE | C4H11NO3 | 121.13717 | osmotic diuretic, alkalizer |  |
| OXTRIPHYLLINE | C12H21N5O3 | 283.33287 | bronchodilator |  |
| CALCIUM GLUCEPTATE | C14H26CaO16 | 490.43372 | calcium replenisher |  |
| ZANAMIVIR | C12H20N4O7 | 332.3158 | antiviral, neuramidase inhibitor |  |
| ACTINOQUINOL SODIUM | C11H10NNaO4S | 275.26045 | ultaviolet screen |  |
| DULOXETINE HYDROCHLORIDE | C18H20ClNOS | 333.8832 | antidepressant |  |
| TERCONAZOLE | C26H31Cl2N5O3 | 532.47467 | antifungal |  |
| MITOTANE | C14H10Cl4 | 320.0478 | insecticide, antineoplastic |  |
| TEMEFOS | C16H20O6P2S3 | 466.4738 | insecticide |  |
| PERMETHRIN | C21H20Cl2O3 | 391.29775 | ectoparasiticide, CNS stimuant, mutagen |  |
| EDITOL | C14H32N2O4 | 292.42214 | alkylyzing agent |  |
| PIRFENIDONE | C12H11NO | 185.22757 | antiinflammatory, analgesic, antipyretic |  |
| MALATHION | C10H19O6PS2 | 330.36113 | pediculicide, insecticide, cholinesterase inhibitor |  |
| SELEGILINE HYDROCHLORIDE | C13H18ClN | 223.74811 | antidepressant, MAO inhibitor, antiparkinsonian |  |
| VIOMYCIN SULFATE | C25H45N13O14S | 783.7801 | antibacterial |  |
| CLINDAMYCIN PALMITATE HYDROCHLORIDE | C34H64Cl2N2O6S | 699.86898 | antibacterial, inhibits protein synthesis |  |
| KETOROLAC TROMETHAMINE | C19H24N2O6 | 376.41293 | antiinflammatory |  |
| IOPANIC ACID | C11H12I3NO2 | 570.93699 | radioopaque agent |  |
| DOCETAXEL | C43H59NO17 | 861.94618 | antineoplastic |  |
| PODOFILOX | C22H22O8 | 414.41584 | antineoplastic, inhibits microtubule assembly, and human DNA topoisomerase II; antimitotic agent |  |
| OMEGA-3-ACID ESTERS (EPA shown) | C22H34O2 | 330.51508 | hypolipidemic |  |
| RIZATRIPTAN BENZOATE | C22H25N5O2 | 391.47685 | 5HT-1B/1D agonist, antimigraine |  |
| GLUTAMINE (L) HYDROCHLORIDE | C5H11ClN2O3 | 182.60802 | dietary supplement |  |
| SENNOSIDE A | C42H38O20 | 862.75916 | cathartic |  |
| DICHLORVOS | C4H7Cl2O4P | 220.97779 | insecticide, cholinesterase inhibitor |  |
| EQUILIN | C18H20O2 | 268.3589 | estrogen |  |
| PRAZOSIN HYDROCHLORIDE [10mg] | C19H22ClN5O4 | 419.87129 | antihypertensive |  |
| ACITRETIN | C19H22O3 | 298.38539 | antipsoriatic |  |
| LIOTHYRONINE (L- isomer) SODIUM | C15H11I3NNaO4 | 672.96222 | thyroid hormone |  |
| PENBUTOLOL SULFATE | C18H31NO6S | 389.51487 | beta-adrenergic blocker |  |
| HALOTHANE | C2HBrClF3 | 197.38247 | anesthetic |  |
| SUCRALOSE | C12H19Cl3O8 | 397.63943 | sweetener |  |
| CYCLAMIC ACID | C6H13NO3S | 179.23941 | sweetener |  |
| RIBOFLAVIN 5-PHOSPHATE SODIUM | C17H20N4NaO9P | 478.33395 | vitamin, enzyme cofactor |  |
| PRALIDOXIME MESYLATE | C8H12N2O4S | 232.25984 | cholinesterase reactivator |  |
| LEVOCARNITINE | C7H15NO3 | 161.2025 | cofactor for fatty acid metabolism, replenisher in arterial disease |  |
| SILDENAFIL CITRATE | C28H38N6O11S | 666.71266 | erectile dysfunction therapy |  |
| SODIUM PHENYLBUTYRATE | C10H11NaO2 | 186.18777 | antihyperammonemic,antineoplastic |  |
| ASENAPINE MALEATE | C21H20ClNO5 | 401.85025 | antpsychotic |  |
| BRINZOLAMIDE | C12H21N3O5S3 | 383.51027 | antiglaucoma |  |
| FLUPIRTINE MALEATE | C19H21FN4O6 | 420.40082 | analgesic |  |
| INOSINE | C10H12N4O5 | 268.23094 | cell function activator, cardiotonic |  |
| CASANTHRANOL [cascaroside A shown] | C27H32O15 | 596.54709 | laxative, antineoplastic |  |
| ADEFOVIR DIPIVOXYL | C20H32N5O8P | 501.48054 | antiviral |  |
| ELTROMBOPAG | C25H22N4O4 | 442.47849 | thrombocytopenia therapy |  |
| PERGOLIDE MESYLATE | C20H30N2O3S2 | 410.6017 | dopamine receptor agonist |  |
| CARMUSTINE | C5H9Cl2N3O2 | 214.05238 | antineoplastic, alkylating agent |  |
| TOLVAPTAN | C26H25ClN2O3 | 448.95375 | vasopressin V2 receptor antagonist |  |
| ACETYLSALICYLANILIDE | C15H13NO3 | 255.27576 | antipyretic, fungicide; prodrug to SALYCYLANILIDE |  |
| QUINESTROL | C25H32O2 | 364.53259 | estrogen |  |
| DIMETHOATE | C5H12NO3PS2 | 229.25809 | insecticide, cholinesterase inhibitor |  |
| INOSITOL | C6H12O6 | 180.15894 | growth factor |  |
| GESTODENE | C21H26O2 | 310.44017 | progestin |  |
| DESOGESTREL | C22H30O | 310.4838 | prgestin |  |
| SAXAGLIPTIN | C18H25N3O2 | 315.41885 | antidiabetic, dipeptidyl peptidase–4 inhibitor |  |
| PHYSOSTIGMINE | C15H21N3O2 | 275.35352 | cholinergic, anticholinesterase, miotic |  |
| NILVADIPINE | C19H19N3O6 | 385.37978 | antihypertensive, antianginal |  |
| TRIAMCINOLONE DIACETATE | C25H31FO8 | 478.51942 | antiinflammatory |  |
| ROFLUMILAST | C17H14Cl2F2N2O3 | 403.21553 | anti-asthmatic |  |
| STRYCHNINE SULFATE | C21H24N2O6S | 432.49923 | central stimulant |  |
| PENCICLOVIR | C10H15N5O3 | 253.26275 | antiviral |  |
| EBASTINE | C32H39NO2 | 469.67313 | antihistamine |  |
| BRIMONIDINE | C11H10BrN5 | 292.13985 | alpha-adrenergic agonist |  |
| FOMEPIZOLE | C4H6N2 | 82.10582 | alcohol dehydrogenase, inhibitor, antidote |  |
| PEMETREXED | C20H19N5Na2O6 | 471.38393 | antineoplastic, thymidylate synthase inhibitor |  |
| LINACLOTIDE (1 mg/ml) | C59H79N15O21S6 | 1526.75938 | irritable bowel therapy, guanylate cyclase 2C agonist |  |
| EMPAGLIFLOZIN | C22H25ClO7 | 436.89335 | antidiabetic, SGLT2 inhibitor |  |
| ABAMECTIN (avermectin B1a shown) | C48H72O14 | 873.10064 | antiparisitic |  |
| EDOXABAN TOSYLATE HYDRATE | C31H40ClN7O8S2 | 738.28755 | Factor Xa inhibitor, prophiaxis of stroke |  |
| FELODIPINE | C18H19Cl2NO4 | 384.26243 | vasodilator, Ca channel blocker |  |
| CLINAFOXACIN HYDROCHLORIDE | C17H18Cl2FN3O3 | 402.25571 | antibacterial |  |
| CLONIXIN | C13H11ClN2O2 | 262.69782 | antiinflammatory, analgesic |  |
| ANAZOLENE SODIUM | C26H16N3Na3O10S3 | 695.59292 | diagnostic aid |  |
| BENDAMUSTINE HYDROCHLORIDE | C16H22Cl3N3O2 | 394.73164 | antineoplastic |  |
| FROVATRIPTAN SUCCINATE | C18H25N3O5 | 363.41705 | 5HT[1B/1D] agonist |  |
| SODIUM PHENYLACETATE | C8H7NaO2 | 158.13359 | antihyperammonemic |  |
| ARECOLINE HYDROBROMIDE | C8H14BrNO2 | 236.11028 | anthelmintic (Cestodes), hypotensive, cathartic |  |
| RIVASTIGMINE TARTRATE | C18H28N2O8 | 400.43246 | acetylcholinesterase inhibitor |  |
| TAZOBACTAM | C10H12N4O5S | 300.29494 | b-lactamase inhibitor |  |
| ACEPROMAZINE MALEATE | C23H26N2O5S | 442.53807 | sedative |  |
| PHENYLALANINE (L) HYDROCHLORIDE | C9H12ClNO2 | 201.65449 | essential amino acid, use in vitiligo treatment |  |
| CETILISAT | C25H39NO3 | 401.59448 | anti-obesity; pancreatic lipase inhibitor |  |
| RAMOPLANIN [A2 shown; 2mM] | C119H154ClN21O40 | 2554.12393 | antibacterial |  |
| ZOLEDRONIC ACID | C5H10N2O7P2 | 272.09225 | bone resorption inhibitor |  |
| RILUZOLE | C8H5F3N2OS | 234.20105 | anticonvulsant, glutamate release inhibitor, anti-ALS |  |
| BICALUTAMIDE | C18H14F4N2O4S | 430.38088 | antineoplastic |  |
| PROCARBAZINE HYDROCHLORIDE | C12H19N3O | 221.30473 | antineoplastic |  |
| OLOPATADINE HYDROCHLORIDE | C21H24ClNO3 | 373.88333 | antihistamine, antiallergic |  |
| POSACONAZOLE | C37H42F2N8O4 | 700.79529 | antifungal |  |
| BIAPENEM | C15H18N4O4S | 350.39911 | antibacterial |  |
| ZONISAMIDE | C8H8N2O3S | 212.22856 | anticonvulsant |  |
| TOSUFLOXACIN TOLUENESULFONATE HYDRATE | C26H25F3N4O7S | 594.57095 | antibacterial |  |
| VOGLIBOSE | C10H21NO7 | 267.28137 | a-glucosidase inhibitor; antidiabetic |  |
| GEMCITABINE HYDROCHLORIDE | C9H12ClF2N3O4 | 299.66349 | antineoplastic |  |
| BUTENAFINE HYDROCHLORIDE | C23H28ClN | 353.93931 | antifungal |  |
| ANASTROZOLE | C17H19N5 | 293.37448 | antineoplastic |  |
| ACETOHEXAMIDE | C15H20N2O4S | 324.40165 | antidiabetic |  |
| MIFEPRISTONE | C29H35NO2 | 429.6078 | progesterone antagonist, abortion inducer |  |
| BENZYL ALCOHOL | C7H8O | 108.14121 | antimicrobial, antipruritic |  |
| BUTOPYRONOXYL | C12H18O4 | 226.27486 | insect repellant |  |
| ARTESUNATE | C19H28O8 | 384.43021 | antimalarial |  |
| METACRESOL | C7H8O | 108.14121 | antiseptic, antifungal |  |
| LEFLUNOMIDE | C12H9F3N2O2 | 270.21293 | antineoplastic, PDGF receptor blocker, immunomodulator |  |
| MEXILETINE HYDROCHLORIDE | C11H18ClNO | 215.72521 | antiarrhythmic |  |
| LANSOPRAZOLE | C16H14F3N3O2S | 369.36808 | antiulcer |  |
| LEVOCARNITINE PROPIONATE HYDROCHLORIDE | C10H20ClNO4 | 253.7282 | carnitine replenisher in peripheral arterial disease |  |
| XYLOSE | C5H10O5 | 150.13245 | diagnostic aid, diabetic food |  |
| INDINAVIR SULFATE | C36H47N5O4 | 613.80709 | antiviral, HIV protease inhibitor |  |
| CIPROFIBRATE | C13H14Cl2O3 | 289.16073 | antihyperlipidemic |  |
| TENOFOVIR | C9H14N5O4P | 287.21683 | antiviral, RT inhibitor |  |
| ATROPINE OXIDE | C17H23NO4 | 305.37716 | anticholinergic |  |
| CARBETAPENTANE CITRATE | C26H39NO10 | 525.60143 | antitussive |  |
| RUFINAMIDE | C10H8F2N4O | 238.19826 | anticonvulsant |  |
| TAMSULOSIN HYDROCHLORIDE | C20H29ClN2O5S | 444.98153 | alpha-1 adrenergic blocker |  |
| CHLORPHENIRAMINE MALEATE | C20H23ClN2O4 | 390.87031 | antihistamine |  |
| DOCOSANOL | C22H46O | 326.61132 | antiviral |  |
| TIOCONAZOLE | C16H13Cl3N2OS | 387.71781 | antifungal |  |

**Table 2. Demographics of 11 patients with sepsis or septic shock.**

| **Patient #** | **Sex** | **Age** | **Sampling Time (h)** | **SOFA** | **pCTS-L (ng/ml)** | **PRO (ng/ml)** |
| --- | --- | --- | --- | --- | --- | --- |
|  | Female | 85 | 0 | 10 | 180.10 | 0.98 |
| 1 |  |  | 24 | 4 | 21.30 | 0.50 |
|  |  |  | 72 | 2 | 2.10 | 0.36 |
|  | Female | 71 | 0 | 11 | 143.60 | 3.45 |
| 2 |  |  | 24 | 3 | 24.10 | 1.76 |
|  |  |  | 72 | 3 | 0.90 | 1.12 |
|  | Female | 67 | 0 | 7 | 1.19 | 6.93 |
| 3 |  |  | 24 | 13 | 2.12 | 3.56 |
|  |  |  | 72 | 9 | 1.58 | 1.25 |
|  | Female | 80 | 0 | 16 | 614.50 | 1.84 |
| 4 |  |  | 24 | ND # |  |  |
|  |  |  | 72 | ND # |  |  |
|  | Female | 62 | 0 | 8 | 32.10 | 0.65 |
| 5 |  |  | 24 | 6 | 14.20 | 0.50 |
|  |  |  | 72 | 2 | 2.50 | 0.40 |
|  | Female | 71 | 0 | 15 | 63.00 | 1.94 |
| 6 |  |  | 24 | 13 | 15.60 | 3.38 |
|  |  |  | 72 | 6 | 9.50 | 0.94 |
|  | Male | 91 | 0 | 7 | 0.98 | 0.75 |
| 7 |  |  | 24 | 5 | 3.45 | 0.32 |
|  |  |  | 72 | 2 | 6.93 | 0.17 |
|  | Male | 92 | 0 | 7 | 1.84 | 0.43 |
| 8 |  |  | 24 | 1 | 0.65 | 0.40 |
|  |  |  | 72 | 1 | 1.94 | 0.36 |
|  | Male | 71 | 0 | 10 | 445.50 | 1.80 |
| 9 |  |  | 24 | 10 | 19.00 | 0.69 |
|  |  |  | 72 | 3 | 4.10 | 0.44 |
|  | Male | 94 | 0 | 12 | 34.10 | 0.49 |
| 10 |  |  | 24 | 11 | 14.20 | 0.39 |
|  |  |  | 72 | 9 | 2.50 | 0.47 |
|  | Male | 72 | 0 | 14 | 45.8 | 1.81 |
| 11 |  |  | 24 | 13 | 1.3 | 0.98 |
|  |  |  | 72 | 9 | 0 | 0.83 |

**Note**s: Sampling time: 0 h – within 24 hours of diagnosis of sepsis or septic shock; 24 h – 24 hours after initial diagnosis of sepsis or septic shock; 74 h – 72 h after initial diagnosis of sepsis or septic shock.

#, ND, not determined because this patient died of septic shock shortly after the diagnosis at time 0.
